# Synthesis, characterization and computational evaluation of bicyclooctadienes towards molecular solar thermal energy storage†

**DOI:** 10.1039/d1sc05791j

**Published:** 2021-12-21

**Authors:** Maria Quant, Andreas Erbs Hillers-Bendtsen, Shima Ghasemi, Mate Erdelyi, Zhihang Wang, Lidiya M. Muhammad, Nina Kann, Kurt V. Mikkelsen, Kasper Moth-Poulsen

**Affiliations:** Department of Chemistry and Chemical Engineering, Chalmers University of Technology Kemigården 4 412 96 Gothenburg Sweden mkasper@chalmers.se; Department of Chemistry, University of Copenhagen, Universitetsparken 5 2100 Copenhagen Denmark; Department of Chemistry – BMC, Uppsala University Husargatan 3 752 37 Uppsala Sweden; The Institute of Materials Science of Barcelona, ICMAB-CSIC 08193, Bellaterra Barcelona Spain; Catalan Institution for Research & Advanced Studies, ICREA Pg. Lluís Companys 23 Barcelona Spain

## Abstract

Molecular solar-thermal energy storage (MOST) systems are based on photoswitches that reversibly convert solar energy into chemical energy. In this context, bicyclooctadienes (BODs) undergo a photoinduced transformation to the corresponding higher energy tetracyclooctanes (TCOs), but the photoswitch system has not until now been evaluated for MOST application, due to the short half-life of the TCO form and limited available synthetic methods. The BOD system degrades at higher temperature *via* a retro-Diels–Alder reaction, which complicates the synthesis of the compounds. We here report a cross-coupling reaction strategy that enables an efficient synthesis of a series of 4 new BOD compounds. We show that the BODs were able to switch to the corresponding tetracyclooctanes (TCOs) in a reversible way and can be cycled 645 times with only 0.01% degradation. Half-lives of the TCOs were measured, and we illustrate how the half-life could be engineered from seconds to minutes by molecular structure design. A density functional theory (DFT) based modelling framework was developed to access absorption spectra, thermal half-lives, and storage energies which were calculated to be 143–153 kJ mol^−1^ (0.47–0.51 MJ kg^−1^), up to 76% higher than for the corresponding norbornadiene. The combined computational and experimental findings provide a reliable way of designing future BOD/TCO systems with tailored properties.

## Abstract 2

Molecular solar-thermal energy storage (MOST) systems are based on photoswitches that reversibly convert solar energy into chemical energy.

## Graphical abstract

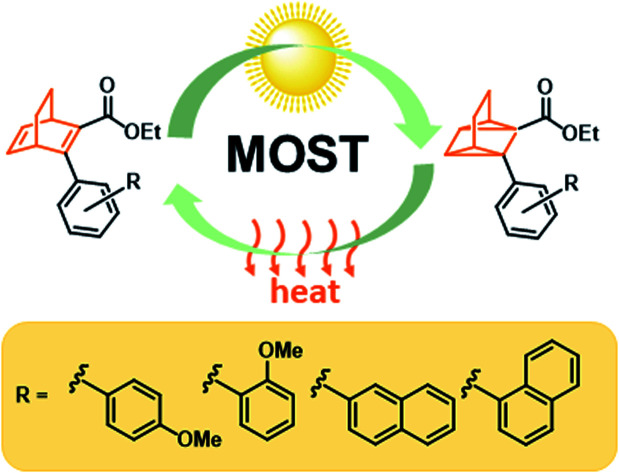

## Introduction

**Fig. 1:**
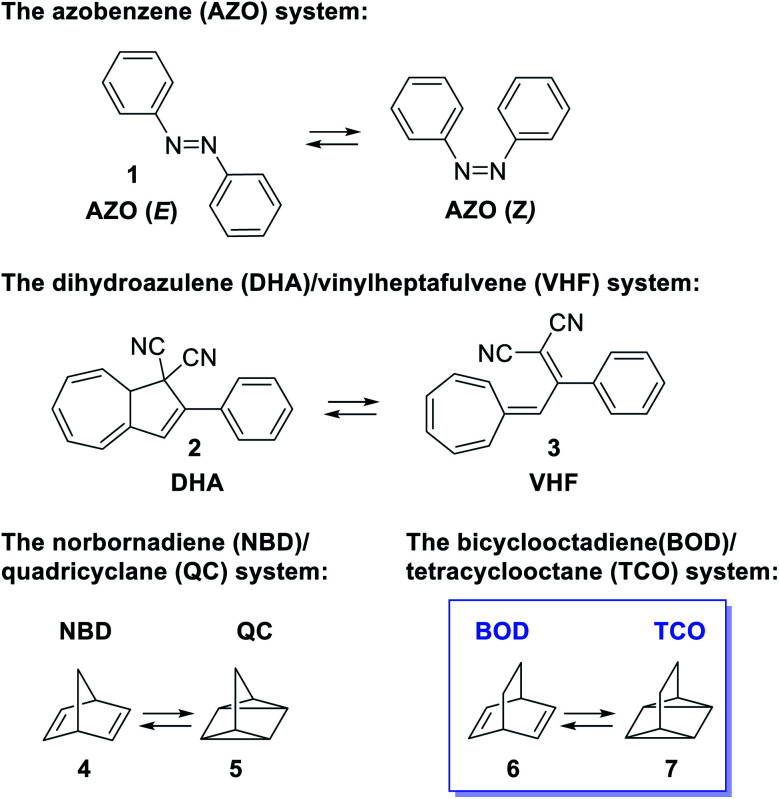
Examples of photoswitches for molecular solar thermal energy storage. The azobenzene (AZO) system, the dihydroazulene (DHA)/vinylheptafulvene (VHF) system, the norbornadiene (NBD)/quadricyclane (QC) system and the bicyclooctadiene (BOD)/tetracyclooctane (TCO) system.

Developing renewable energy sources is one of the great challenges the world is facing. One possible way is to store the energy from the sun using a concept called molecular solar thermal energy storage (MOST).^1,2^ MOST is based on molecular photoswitches that can absorb light and store part of the energy in a metastable photoisomer. The photoisomer is storing the energy and when triggered, the energy is released and the photoisomer is converted back to the parent compound. Unlike traditional photochemical energy storage in plants, the MOST systems operate in a closed cycle with light as the input and heat on demand as the output. Examples of promising molecular systems (Fig. 1) are the azobenzene^3–5^ system (AZO) (1) that upon irradiation undergoes a *E*–*Z* isomerization, the dihydroazulene^6–9^ (DHA) system (2) that is converted to the photoisomer vinylheptafulvene (VHF) (3) and the norbornadiene^10–14^ (NBD) system (4) that undergoes a [2 + 2] cycloaddition to the photoisomer quadricyclane (QC) (5). A structurally very similar compound to norbornadiene is bicyclooctadiene (BOD) (6), also a cyclic compound that contains two double bonds in a ring structure. The difference is the additional carbon at the bridgehead position (Fig. 1). Upon irradiation, BODs can be converted to the corresponding photoisomer tetracyclooctane (TCO) (7).^15–18^

**Scheme 1:**
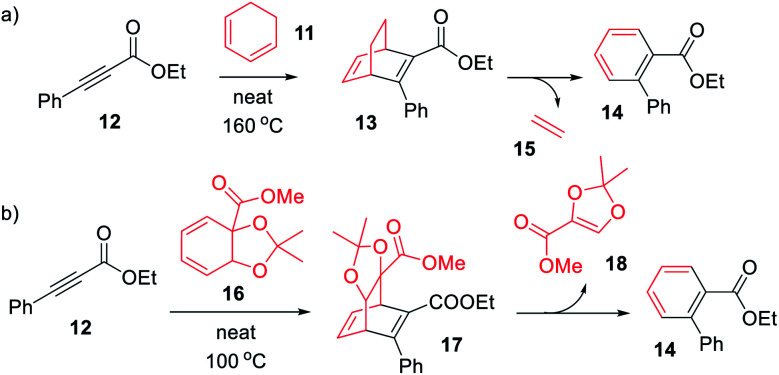
Synthesis of (a) BOD (13) and (b) BOD (17) *via* a Diels–Alder reactions, with the concomitant formation of byproduct (14).

**Fig. 2:**
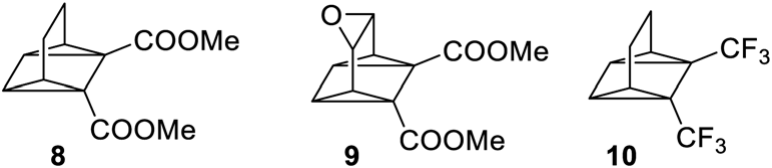
Some selected examples of tetracyclooctadienes reported by Gleiter *et al.*^19^

The photophysical properties of BODs have been much less explored than other systems, one reason being that the photoisomer, TCO, is rapidly converted to BOD through thermal activation. Also, upon heating, BODs can degrade into an aromatic byproduct *via* a retro-Diels–Alder process and since the gaseous ethene is lost during the process, the reaction is irreversible (Scheme 1). This degradation has also been seen under photochemical conditions.^15^ However, several examples of BODs and the corresponding TCOs have been isolated and reported in the literature. ^15–18^ For example, Gleiter *et al.*^19^ described the formation of TCOs (Fig. 2). The methyl ester-substituted (8) could not be isolated but proven by trapping with HCl, while 9 containing an epoxide functionality, could be isolated at 0 °C. TCO (10), with trifluoromethyl groups, was found to be more stable, with a half-life of 5.5 minutes at 80 °C.^19^

More recent work has focused on the synthesis of chiral BODs for applications such as acting as chiral ligands for asymmetric synthesis.^20–22^ Various BODs have also been synthesised through asymmetric gold-catalysed Diels–Alder reactions between alkynes and cyclohexadienes.^23^

Recently, we have performed a computational study where the BOD systems showed very promising properties for energy storage. The storage energy for the unsubstituted NBD/QC system is 0.97 MJ kg^−1^ while the corresponding value for the unsubstituted BOD/TCO is 1.77 MJ kg^−1^.^24^ Therefore, we became interested in evaluating BODs as candidates for MOST. Known BOD systems absorb in the UV range of the solar spectrum,^25^ and molecular engineering is needed to red-shift the absorption spectrum towards more intense parts of the solar spectrum. For structurally similar NBD/QC systems, the introduction of donors and acceptor groups at the 2 and 3 positions have been shown to be an effective strategy for modifying the optical properties.^1^ Since BODs and NBDs operate through the same photoisomerization process, one interesting question here is how this translates to the BOD/TCO system and if molecular engineering of other properties, such as thermal stability of the photoisomers, can be implemented. We here report the synthesis of a series of donor/acceptor-substituted BODs and a study their photophysical behavior. For the first time, BODs are evaluated as MOST candidates from both an experimental and computational perspective.

## Result and discussion

### Synthesis

We here present the initial attempts aimed at synthesizing BODs in a one-step procedure, starting from cyclohexadiene (11, Scheme 1a) and alkynes containing electron donating and electron accepting groups. Ethyl-3-phenylpropiolate (12) and cyclohexadiene were used as a test system and a series of small-scale reactions to optimize the reaction conditions were carried out (ESI, Table S2.1†). The conclusion was that the conversion to the desired bicyclooctadiene (13) reached a maximum of 30% yield. The high temperatures required for the reaction to proceed favored the formation of the aromatic retro-Diels–Alder byproduct, ethyl [1,1′-biphenyl]-2-carboxylate (14), that was formed in 48% yield. Attempts to catalyze the reaction with AlCl_3_ and (2-bromophenyl)boronic acid were not successful. Since the formation of ethene (15) is a driving force for the byproduct formation, we speculated that using a more substituted cyclohexadiene could prevent the retro-Diels–Alder reaction. Methyl 2,2-dimethylbenzo[*d*][1,3]dioxole-3a(7a*H*)-carboxylate (16, Scheme 1b) had previously been prepared by Paterson *et al.*^26^ and was provided to us for testing in the Diels–Alder reaction with 12. In this case the outcome was the same and 14 was again the favored product, indicating that the leaving group, methyl 2,2-dimethyl-1,3-dioxole-4-carboxylate (18), was stable enough to drive the reaction towards byproduct formation.

**Scheme 2:**
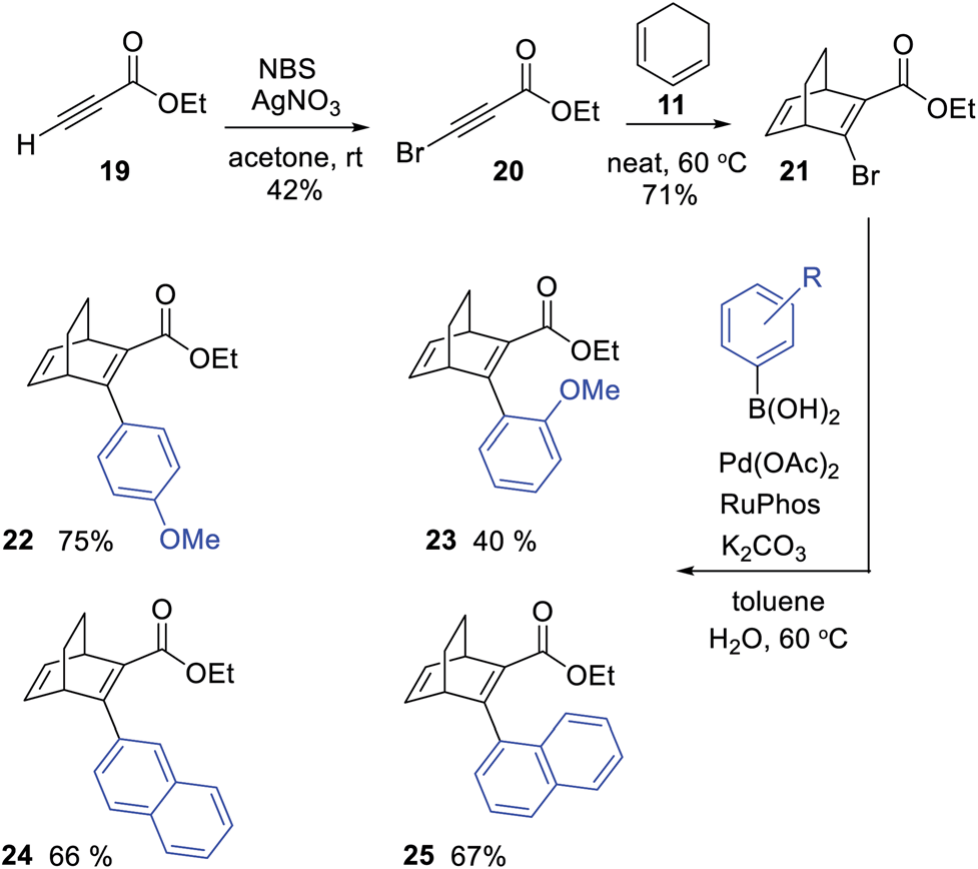
Synthesis of BODs 22 to 25*via* a three-step route, involving a Diels–Alder reaction followed by Suzuki cross-coupling.

A new strategy for obtaining the BODs was thus necessary, and our idea was to instead perform the Diels–Alder reaction with an alkyne containing an acceptor group and a halogen, and thereafter to employ a cross-coupling reaction to introduce the donor group.^27^ Inspired by Bosse *et al.*,^28^ ethyl propiolate (19) was brominated with NBS to produce ethyl 3-bromopropiolate (20) (Scheme 2). Thereafter, 20 was reacted in a Diels–Alder reaction at 60 °C with cyclohexadiene to afford ethyl-3-bromobicyclo [2.2.2]octa-2,5-diene-2-carboxylate (21) in a yield of 71%. Compound 21 was used directly in a Suzuki cross-coupling reaction to obtain BODs 22–25. The reaction temperatures were kept under 60 °C to avoid a retro-Diels–Alder reaction and a series of BODs were successfully synthesized in 40 to 75% yields (Scheme 2). The choice of donor groups was based on finding suitable substituents to increase the stability of the corresponding TCOs (26–29) (ESI Fig. S2.1†). Previous studies on NBDs have shown that steric repulsion hindered the rotational motion of the side groups along the back-conversion path to prolong the half-lives of QCs. Placing the methoxy group in the *ortho* position instead of in the *para* position, resulted in an increase in the half-life of 75 times for the NBD system.^29^ One objective here was to explore whether and how these concepts could be translated to the BOD/TCO system.

**Fig. 3:**
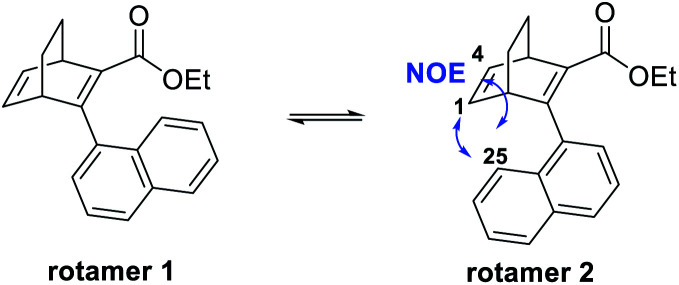
Two different rotamers of 25.

Furthermore, we noted that 25 was isolated as two rotamers at a ratio of 1 : 2.5 at room temperature, due to hindered rotation around the single bond that connects the naphthyl group with the BOD core. 2D-NMR was performed to distinguish the rotamers and the NOE between H-25 and H-1 as well as H-25 and H-4 for the major species revealed it to be rotamer 2 (Fig. 3 and ESI, Fig. S3.10–S3.16†). Also, a variable temperature NMR study was performed in DMSO at 65 °C, 95 °C and 130 °C (ESI, Fig. S3.8†). As the temperature was increased, peaks from the rotamers started to coalesce and at 130 °C, only single peaks were observed. However, quickly after heating the sample up to 130 °C, compound 25 started to degrade and new peaks in the aromatic region of the spectra and a new triplet at around 0–1 ppm were formed. These peaks correspond to the byproduct formed through a retro-Diels–Alder reaction. Upon cooling the sample, the new peaks from the byproduct remained and the signals of the different rotamers reappeared, confirming the interpretation of the high temperature NMR data.

### Photophysical evaluation

#### Absorption profiles

**Fig. 4:**
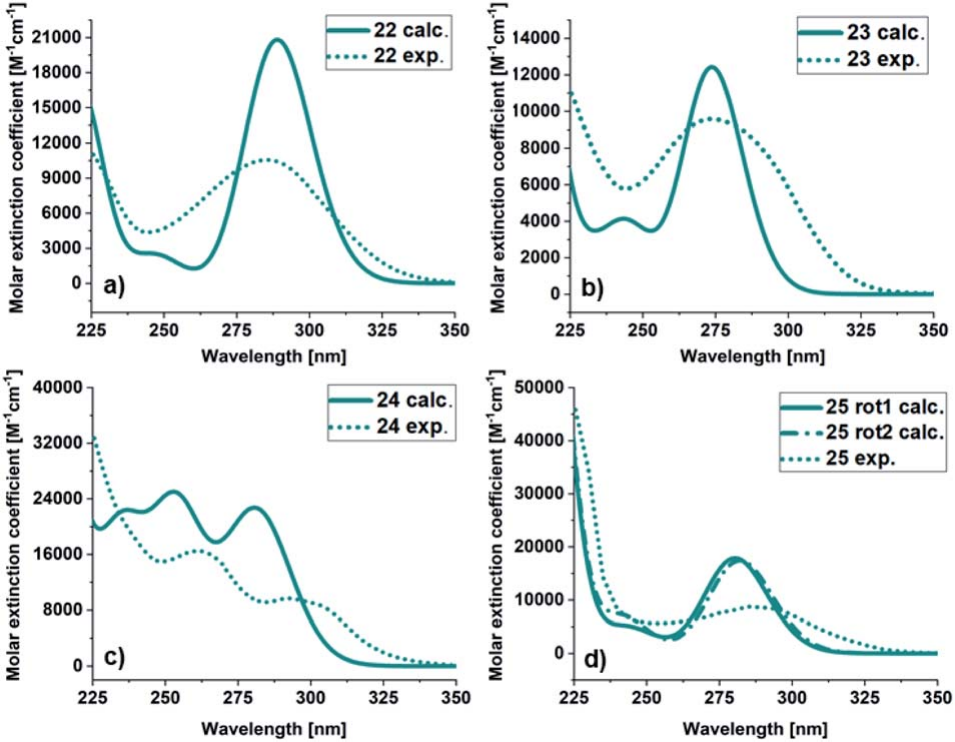
UV/Vis spectra of BOD (a) 22, (b) 23, (c) 24 and (d) 25. Calculated (green line), experimental in MeCN (dashed green line).

The UV/Vis spectra of 22–25 were recorded in acetonitrile and calculated using time dependent DFT (TD-DFT) based modelling at the M06-2X/6–311++G (d,p) level of theory with solvent effects included *via* the polarizable continuum model (PCM) (Fig. 4, Table 1). In general, the experimental and calculated spectra are in good agreement. For 22, the experimental onset is at 350 nm and the calculated value is 332 nm, but the shape of the spectra is similar. The molar extinction coefficient at the absorption maxima is higher for the calculated (20 813 M^−1^ cm^−1^) than for the experimental (10 568 M^−1^ cm^−1^) spectrum. For 23, the calculated and experimental molar extinction coefficient are similar (12 425 M^−1^ cm^−1^ and 9954 M^−1^ cm^−1^ respectively), and the maximum the same (274 nm). The onsets are at 338 nm for the experimental and 310 nm for the calculated spectra. The spectra of 24 has a different shape compared to the other BODs. It has two bands and therefore two local maxima both in the experimental (292 nm and 262 nm) and calculated (281 nm and 253 nm) case. Otherwise, the trend is similar, with a slightly more redshifted onset for the experimental spectrum compared to the calculated spectrum. The molar extinction coefficient is lower for the predicted values (22 729 M^−1^ cm^−1^ and 25 013 M^−1^ cm^−1^) compared to the experimental values (9673 M^−1^ cm^−1^ and 16 493 M^−1^ cm^−1^).

**Table 1:** Absorption profiles. Experimental (exp.) and calculated (calc.) absorption onset (*λ*_onset_), absorption maxima (*λ*_max_), and molar extinction coefficient at the absorption maxima (*ε*_max_) for BODs 22–25

BOD	Experimental			Calculated		
	*λ*_onset_ a [nm]	*λ*_max_ b [nm]	*ε*_max_ b [M^−1^ cm^−1^]	*λ*_onset_ a [nm]	*λ*_max_ b [nm]	*ε*_max_ b [M^−1^ cm^−1^]
22	350	285	10 568	332	289	20 813
23	338	274	9594	310	274	12 425
24	350	292	9673	323	281	22 729
		262	16 492		253	25 013
25	348	285	8757	320^c^	280^c^	17 855^c^
				322^d^	282^d^	17 141^d^

a *λ*_onset_ defined as log(*ε*) = 2.

b *ε*_max_ and *ε*_max_ refers to all maxima above 225 nm. For 24 two peaks were obtained.

c Data for rotamer 1.

d Data for rotamer 2.

For 25, obtained as two different rotamers at room temperatures, the spectra for both rotamers were calculated (Table 1). Also in this case the trend is the same, the onset and the maxima being slightly shifted toward higher wavelengths compared to the experimental spectra. The UV/Vis spectra for the corresponding TCOs and aromatic byproducts (30–33) were also obtained experimentally and computationally (ESI, Fig. S5.3 and S5.4†) and are in good agreement. Additionally, the UV/Vis spectra for the corresponding NBDs (34–37) were calculated (ESI, Fig. S5.5,† see also ESI, Fig. S2.1†).

#### Photoisomerization

**Fig. 5:**
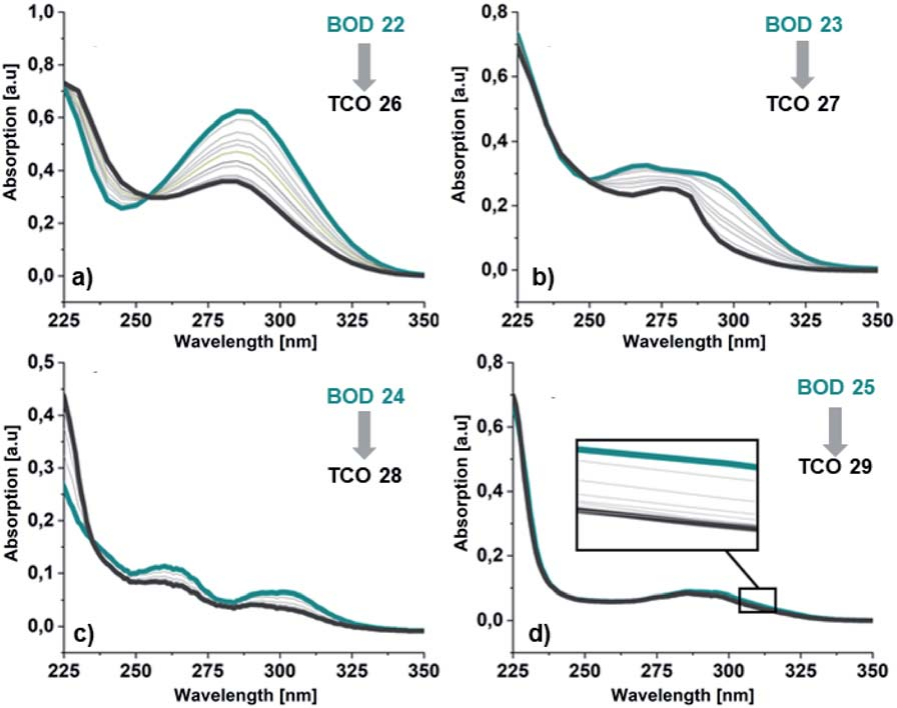
Absorption spectra in MeCN for the irradiation of BODs (green line) to the corresponding TCOs (dark grey line) (a) 22 and 26, (b) 23 and 27, (c) 24 and 28 and (d) 25 and 29.

To convert the BODs to the corresponding TCOs, they were dissolved in acetonitrile and irradiated stepwise with a 310 nm LED. The temperature was kept between 0–2 °C, to minimize back conversion reactions (Fig. 5). For all BODs, the stepwise irradiation resulted in a stepwise decrease in absorption around the first absorption maximum. Isosbestic points were also observed in all spectra. The spectral change was less than what is commonly observed for NBDs. An explanation may be that overlapping spectra for the BODs and the TCOs leads to a photostationary state instead of full conversion. For 24 and 25, the overall decrease in absorption upon irradiation was less than for 22 and 23. An explanation can be that there is a greater overlap between the spectra of the corresponding TCOs and the BODs, which can also be seen in the calculated spectra (ESI, Fig. S5.1†). The stepwise irradiation was also performed in toluene with near identical results, indicating negligible solvent effects (ESI, Fig. S5.6†). To confirm the switching and to see that the BODs were not degrading to the corresponding byproducts during irradiation, samples of all BODs were irradiated for 30 min and proton NMR spectra were then directly recorded at −20 °C. For all BOD/TCO systems, peaks corresponding to the TCOs appeared after irradiation and after keeping the sample at room temperature, the BODs were recovered (ESI, Fig. S3.22–S3.29†).

Additionally, a thin layer chromatography (TLC) analysis was performed on the byproducts and the BODs before and after irradiation. Despite the retention factor of the byproducts and the corresponding BODs being similar in all tested eluent systems, the analysis showed clean spots that clearly indicated that the byproducts were not present in the BOD samples, either before or after irradiation (ESI, Fig. S6.1†). The quantum yields for the photoisomerization process in toluene were measured at 5 °C to 14% and 19% for 23 and 25, respectively (ESI, Fig. S6.1†). The fast back-conversion for 22 and 24 due to their short half-lives provided an uncertainty to the measurements and therefore quantum yields for these two compounds are not reported.

#### Back-conversion

To obtain the half-lives of the TCOs (26–29), the rates of the back-conversion were measured at several different temperatures (ESI, Fig. S4.1–4.4†). The half-lives were measured in both toluene and acetonitrile, since previous studies in our group have shown that going from a non-polar to a polar solvent can change the half-lives for NBDs with a factor of up to 2.^30^ The results are presented in Table 2. The effect on the half-lives from a bulky substituent in the *ortho* position compared to the *para* position is large. For the BODs substituted with methoxy groups, the half-life is around 10 times longer with this substituent in the *ortho* position as compared to the *para* position. Regarding the naphthyl group, the difference is even larger, the half-life of 29 being ∼60 times longer than for 28. Thus, the half-lives of the TCOs follow the same trend as for similar QCs. For NBDs, substitution in the ortho position causes a dramatic change in the QC landscape, but not in the landscape of the transition state. The bulky substituents affect the rotational motion of the side group relative to the parent compound, both in terms of the vibrational and configurational degrees of freedom and give rise to a larger entropy of back-conversion.^29^ Since the isomerization process and back-conversion of BODs and NBDs works through the same general mechanism, it is reasonable to believe that this is also the explanation in the BOD case.

**Table 2:** Experimentally obtained half-lives (*t*_1/2_) of the corresponding photoisomers TCOs (26–29) in toluene and in acetonitrile, determined from Eyring parameters at 25 °C

TCO	*t*_1/2_ [s] toluene	*t*_1/2_ [s] MeCN
26	10.1	7.6
27	99.6	79.8
28	5.6	4.1
29	276	282

However, for the BODs, the half-lives are in general much shorter than for similar NBDs.^30^ Also, in these systems the differences in the half-lives between the different solvents are small and could be neglected. This may indicate that the back-conversion occurs *via* a non-polar mechanism.

#### Cyclability

**Fig. 6:**
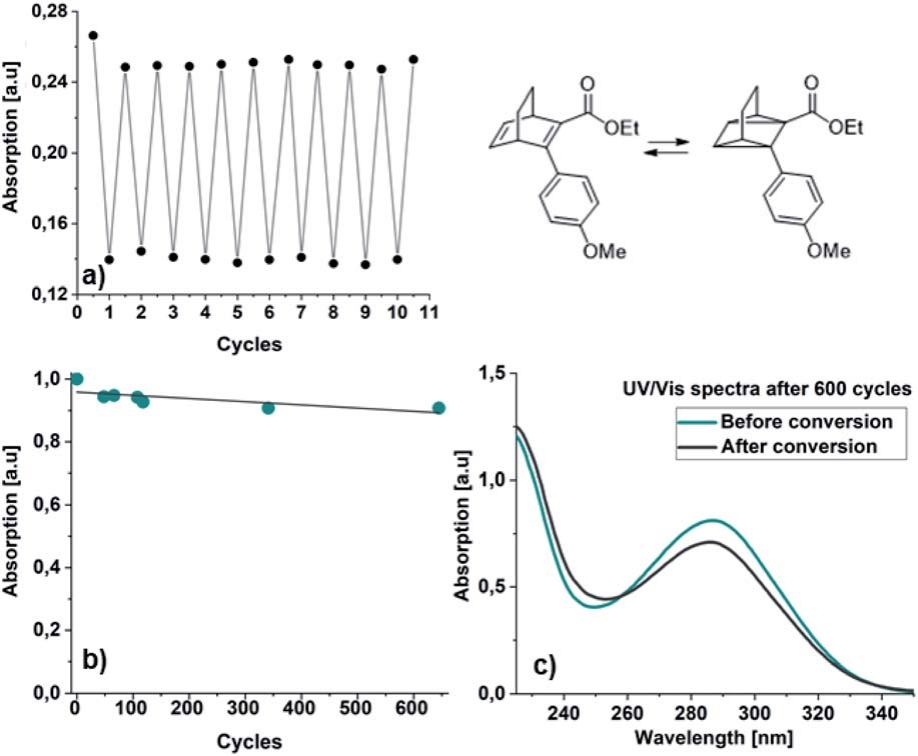
Cycling study for 22: (a) manual cycling study showing the absorption at 310 nm before and after irradiation, (b) automated cycling study showing the absorption of back converted 22 after a number of cycles (green dot) and (c) the shape of the UV/Vis spectra after 600 cycles before (green line) and after (grey line) irradiation.

A qualitative switching study of all BODs in acetonitrile was carried out. All compounds were irradiated at low temperatures to form the corresponding TCOs, then heated to 40 °C for 5 or 10 min to ensure complete back-conversion (ESI, Fig. S8.1†). The process was repeated 4–5 times and all BODs could be switched. The shapes of the BODs absorption spectra were consistent after all cycles, and no byproducts were observed. In case the corresponding aromatic byproduct would have been formed, we synthesized the corresponding aromatic compounds and recorded their UV-Vis spectra (ESI, Fig. S5.2†), illustrating how different these are and thus verifying that retro-Diels–Alder is not occurring in the photochemical conversions at these temperatures. The cyclability for 22 was further tested manually in 10 cycles (Fig. 6a). Interestingly, in the first cycle there is some decrease in absorption, while the switching occurs without any significant degradation for the next 9 cycles. While the system here is operated in diluted solution, we cannot rule out that similar processes are at play here. Other explanations can be that residual oxygen and traces of water effected the first cycle. To further test the cyclability in a more precise way, 22 was cycled 645 times in an automatic set up under nitrogen atmosphere with irradiation at 5 °C for 4 minutes, then allowing 20 min for complete conversion back. During the switching study, the UV/Vis spectra was recorded 7 times to calculate the overall degradation to 0.01% (Fig. 6b and c, ESI, Fig. S8.2†). This indicates that the system is very robust and can operate for a long time with almost no degradation. Also, the UV/Vis spectra were recorded after 645 cycles and remained consistent after the cycling experiment. By comparing the spectra after irradiation for 22 in Fig. 5 and 6, it appears as if the maximum conversion was not obtained in the cycling study. This was not the case since irradiation for 4 min is enough for maximum conversion and this is instead a consequence of the fast back-conversion.

#### Light to chemical energy conversion efficiencies

The very high energy storage densities of 143–153 kJ mol^−1^ (0.47–0.50 MJ kg^−1^) made us interested in how efficient these systems are in the storage of solar energy. Following the method by Herges *et al.*,^31^ we calculated the efficiency of the light to chemical energy for 23 and 25 to 4.8% and 6.8%, respectively. These values are higher than for the bare azobenzene system, which has an efficiency of 1.4%, but not in the range of diindanodiazocine, which has an efficiency of 18.1%.^31^ The main loss factors in the system is the low quantum yield, *i.e.* 14% and 19%.

#### Computed energy barriers, storage densities and storage energies

**Fig. 7:**
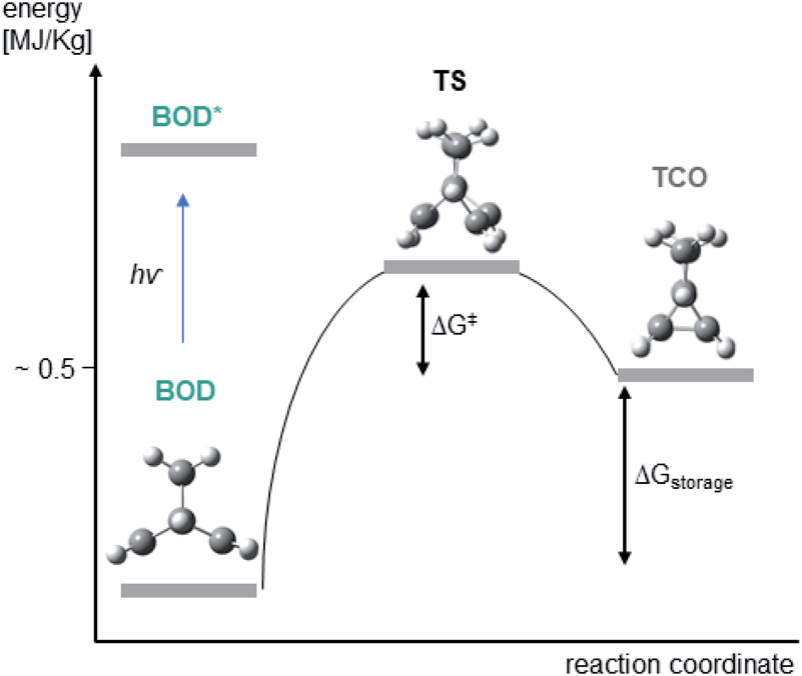
Energy diagram illustrating the conversion of BOD to TCO *via* a transition state (TS) along with the computed structures of BOD, TCO and TS.

The storage densities, storage energies and reaction barriers for the thermal back-conversion reaction and for the retro-Diels–Alder reaction to the byproduct were further assessed using DFT-based modelling for the BOD and also for the corresponding NBD systems (Table 3). For all BOD/TCO systems, the observed storage energies are in the range of 143–153 kJ mol^−1^ which is an increase of 49–76%. Storage densities are in the range of 0.47–0.51 MJ kg^−1^, which is an increase of 38–61%, compared to the corresponding NBD/QC systems. This indicates that utilizing BOD/TCO systems for storing solar energy has an enormous benefit. The larger potential storage energy in the BOD system compared to NBD, can be explained by the structural difference of BOD and TCO (Fig. 7, ESI, Table S8.2†). The BODs are in general eclipsed, while the transition state (TS) and TCO feature a larger degree of staggered conformation, which in turn leads to a higher degree of strain as compared to NBD/QC.

**Table 3:** Calculated storage energies (Δ*G*_storage_) at 298.15 K, storage densities (Δ*G*_storage density_) at 298.15 K, barrier for the byproduct formation (BP_barrier_), barrier for the back reaction (Δ*G*^‡^) for BODs 22–25 and the corresponding NBDs 34–37

BOD/TCO or NBD/QC	Δ*G*_storage_ [kJ mol^−1^]	Δ*G*_storage density_ [MJ kg^−1^]	Δ*G*^‡^ [kJ mol^−1^]	BP_barrier_ [kJ mol^−1^]
22/26	143.03	0.50	118.39	127.11
23/27	146.27	0.51	149.22	120.86
24/28	152.9	0.50	120.53	128.28
25/29	142.48^a^	0.47^a^	131.26^a^	126.21^a^
	147.89^b^	0.49^b^	122.77^b^	123.21^b^
34/38	57.41	0.21	176.78	—
35/39	54.51	0.20	176.37	—
36/40	62.60	0.22	169.96	—
37/41	51.61^a^	0.18^a^	184.03^a^	—
	54.75^b^	0.19^b^	171.22^b^	

a Data for rotamer 1.

b Data for rotamer 2.

Turning our attention to the thermal back reaction barriers, these are predicted to lie within the 118–150 kJ mol^−1^ range. This is relatively large and corresponds to half-lives that are at least 10^7^ s at 298.15 K using the Eyring equation indicating that BOD/TCO systems can, in fact, store solar energy for quite significant periods of time. However, compared to the experimentally observed half-lives, there is a discrepancy, given that the experimental half-lives are a few seconds to a few minutes.

We attribute some differences to arise from the multireference character of the singlet biradical transition states. The significant multireference character of these singlet biradical transition states have been shown to have a large effect on the predicted barriers of similar NBD/QC systems in a previous study by Kuisma *et al.*,^14^ Their calculations show that standard DFT methods, as utilized here, on average give thermal back-reaction barriers that are ∼28% larger than those obtained utilizing (16,16)-complete active space second order perturbation theory (CASPT2) calculations. If we assume that the calculated back reaction barriers of this study are 28% too large and correct them by that amount, then we obtain barriers which correspond to half-lives that range from a minute to hours for the four systems. This is in much better agreement with the experiments, since the exponential form of the Eyring equation means that small deviations in the barriers translate into large differences in the half-lives. The inability of the M062X functional in modelling the singlet biradical character of the transition states is the main deficiency of our current computational modelling. It is clear that performing multireference calculations would be preferable, yet, these calculations are rather tedious and time consuming and cannot be applied routinely. Another solution would be to utilize DFT functionals that are better at describing singlet biradical transition states. This has been done to great success using spin-flip methods in previous investigations and these strategies could be beneficial for future work on BOD/TCO systems.^32–39^ However, we have to emphasize that the predicted back reaction barriers still qualitatively match the experimentally observed half-lives. Furthermore, the problem of large multireference character is restricted to situations in which two or more electronic energy levels are degenerate. In this case and for the NBD/QC systems, such a situation is only encountered during bond breaking in the transition state structures.^14^ Consequently, we do not expect that these effects influence the computed storage energies (Fig. 7).

A critical challenge with the BOD/TCO system is the degradation *via* retro-Diels–Alder reactions (Scheme 1). The computations predict energy barriers that are quite large for retro-Diels–Alder reactions, indicating that the BOD compounds are stable at ambient temperatures. To test the heat tolerance experimentally, all BODs were dissolved in toluene and heated to 75 °C for 1 hour. NMR analysis showed no degradation and aromatic byproducts from retro-Diels–Alder reactions were not detected (ESI, Fig. S3.17†).

## Conclusions

We have developed a series of novel BODs, established efficient synthetic routes to them and evaluated their photophysical properties both computationally and experimentally. Hence, our two-step synthetic approach that makes use of a Diels–Alder reaction followed by a Suzuki coupling provided a series of new donor/acceptor BODs with redshifted optical absorption. All BODs were successfully photoswitched to the corresponding photoisomers for several cycles without significant degradation. However, the rather small spectral changes upon irradiation are probably a due to a combination of short storage times and a photostationary state between the BODs and TCOs, as a consequence of overlapping spectra. BOD 22 could be cycled for 645 cycles with almost no degradation. By introducing bulky substituents in the *ortho* position, the half-lives of the TCOs increased ∼60 times, from seconds to minutes. The BOD/TCO system is demonstrated to possess several of the functions necessary for MOST applications, in terms of reversible photo-thermal conversions and high computed energy densities (143–153 kJ mol^−1^, 0.47–0.50 MJ kg^−1^). Yet we note that the spectral properties and storage time of the system must be improved. Future work will focus on improvement of the storage times of the current model systems, which were used to provide a proof of principle. For our next series of donor/acceptor BODs, we want to introduce bulky substituents on the bridge with the aim of improving the storage times, which previously has been a successful strategy for prolonging the half-lives of QCs.^40,41^ We envision that the established computational framework and the molecular engineering concepts presented here will be helpful in developing future functional MOST systems, based on the BOD/TCO based photoswitch system.

## Experimental

Details on the synthesis and characterizations of BODs and any associated references are given in the ESI.†

## Author contributions

M. Q. main author and responsible for writing original draft, performed optimization of reactions conditions, synthesis of BODs and precursors. UV/Vis experiments, cyclization study, kinetic study of the back-conversion and NMR experiments. A. E. H.-B. second author, performed all computations and assisted MQ with writing the draft. S. G. performed synthesis of BP1-4 and measured their UV/Vis absorption. Z. W. performed quantum yield measurements of BODs and long-term cyclization study. L. M. M. performed quantum yield measurements of BODs. M. E. performed 2D-NMR, high temperature NMR, to analyse rotamers of BOD4 and low-temperature NMR to confirm TCO structures with M. Q. N. K. supervised M. Q giving advice during synthesis and the experimental part of the work. K. V. M. corresponding author, supervised. A. E. H.-B. K. M.-P. corresponding author, designed and supervised the project and was responsible for funding acquisition.

## Conflicts of interest

There are no conflicts to declare.

## Supplementary Material

SC-013-D1SC05791J-s001
