# Supplementary material for: Synthesis, characterization and computational evaluation of bicyclooctadienes towards molecular solar thermal energy storage
Source: Chem Sci. 2021 Dec 21;13(3):834–41. doi: 10.1039/d1sc05791j (PMC8768882; doi:10.1039/d1sc05791j)
Supplement: SC-013-D1SC05791J-s001 [file SC-013-D1SC05791J-s001.pdf]

Electronic Supplementary Material (ESI) for Chemical Science.
This journal is © The Royal Society of Chemistry 2021

SUPPORTING INFORMATION

Synthesis, Characterization and Computational Evaluation of Bicyclooctadienes Towards Molecular Solar Thermal Energy Storage

Maria Quant, Andreas Erbs Hillers-Bendtsen, Shima Ghasemi, Mate Erdelyi, Zhihang Wang, Lidiya M. Muhammad, Nina Kann, Kurt V. Mikkelsen,* Kasper Moth-Poulsen,*

Table of Contents

1.Synthesis 2
2. Numbering and structures of compounds 5
3. NMR spectroscopy 6
4. Kinetic study of back-conversion reaction 27
5. UV/Vis spectroscopy 30
6. TLC experiments 34
7. Quantum yield measurements 35
8. Cyclization studies 37
9. Computations 38
10. References 79

1.Synthesis

General Information
All commercial chemicals were used as received. Toluene was dried on an MBraun MB SPS-800 solvent purification system. Column chromatography was performed on a Biotage Isolera One instrument using pre-packed silica columns (25g or 50g Biotage® SNAP Cartridge). All spectrophotometric analyses were performed using a Cary 50 Bio - or a Cary 100 -UV-Visible spectrophotometer. ^{1}H-NMR and ^{13}C-NMR spectra were obtained at 400 and 100 MHz respectively, using a Varian MR-400 spectrometer. High and low temperature and 2D NMR was performed at NMR Uppsala infrastructure. Low temperature NMR spectra were recorded at Uppsala University on a 500 MHz Bruker Avance Neo NMR spectrometer equipped with a TXO cryogenic probe, the system being precooled for rapid acquisition after the photoswitching. High temperature NMR spectra for identification of coalescence of conformers as well as of the retro-Diels-Alder reaction byproducts were run at a Varian 400 MR system equipped with a SmartProbe at Uppsala University. Chemical shifts are reported in ppm with solvent residual as internal standard ($CHCl_3$ δ_H 7.26, $CHCl_3$ δ_C 77.16). IR-analysis were carried out on a PerkinElmer FT-IR. Elemental-analysis were performed twice for each compound on a Elementar vario MICRO cube, and the data is presented as an average of the two measurements. Ethyl-3-bromopropilate (20) was synthesized according to published procedure.[1]

General procedure A
A nitrogen or argon degassed solution of ethyl-3-bromobicyclo[2.2.2]octa-2,5-diene-2-carboxylate (1 equiv) or ethyl 2-bromobenzoate in toluene was added to a degassed flask containing boronic acid (1.2 equiv), K_2CO_3 (4.6 equiv), RuPhos (10 mol%,) and $Pd(OAc)_2$ (4.8 mol%,). Nitrogen purged H_2O (6 ml) was added and the biphasic mixture was stirred at 60 °C or 80 °C. The reaction was monitored by TLC and after completion cooled to room temperature, quenched with saturated aqueous NH_4Cl (20 mL) and diluted with water (20 mL). The mixture was extracted with toluene (3 x 20 mL), the combined organic phases were dried over Mg_2SO_4, filtered and the solvent removed under reduced pressure. The crude was subjected to automated flash column chromatography.

Ethyl-3-bromobicyclo[2.2.2]octa-2,5-diene-2-carboxylate (21). In a sealed microwave vial ethyl-3-bromopropiolate (4.7 g, 0.026 mol, 1 equiv) was added and heated to melt at 60 °C. 1,3-cyclohexadiene (1,2 equiv, 0.03186 mol, 2.55 g) was added. The reaction mixture was heated for 4 days at 60 °C. The reaction was cooled to rt and subjected to automated flash column chromatography (gradient 0-4% EtOAc in petroleum spirits. Ethyl-3-bromobicyclo[2.2.2]octa-2,5-diene-2-carboxylate was isolated to a yield of 71%. ^{1}H NMR (400 MHz, $CDCl_3$ with TMS): *δ*=6.36 (ddd, *J* = 7.4, 6.0, 1.6 Hz, 1H; HRC=CR*H*), 6.30 (ddd, *J* = 7.3, 5.9, 1.9 Hz, 1H; *H*RC=CRH), 4.32 (dq, *J* = 5.6, 1.7, 1.1 Hz, 1H; *H*R_3C), 4.28 – 4.17 (m, 2H; C*H*$_2$), 3.90 (dh, *J* = 5.8, 1.5 Hz, 1H; *H*R_3C), 1.63 – 1.56 (m, 1H; RH_2C-C*H*$_2$R), 1.51 – 1.43 (m, 1H; RH_2C-C*H*$_2$R), 1.41 – 1.27 (m, 5H; R*H*$_2$C-CH_2R and RC*H*$_3$), ^{13}C NMR (100 MHz, $CDCl_3$ with TMS)): *δ*=164.1, 135.1, 134.9, 134.3, 132.4, 60.9, 50.9, 40.3, 24.9, 24.9,14.4.

Ethyl-3-(4-methoxyphenyl)bicyclo[2.2.2]octa-2,5-diene-2-carboxylate (22) Ethyl-3-bromobicyclo[2.2.2]octa-2,5-diene-2-carboxylate (1 equiv, 0.0021 mol, 0.55g) and 4-methoxyphenylboronic acid (1.2 equiv, 0.00252 mol, 0.38 g) were reacted at 60 °C for 2 days, according to general procedure A. After column chromatography (gradient 0-10% EtOAc in petroleum spirits) **22** (0.45g, 75%) was obtained as a clear oil. R_f=0.58 (EtOAc/Hexane, 1:4);^{1}H NMR (400 MHz, $CDCl_3$ with TMS): *δ*=7.21 – 7.17 (m, 2H; Ar-*H*), 6.89 – 6.83 (m, 2H; Ar-*H*), 6.47 (ddd, *J* = 7.6, 6.1, 1.6 Hz, 1H; *H*RC=CRH), 6.39 (ddd, *J* = 7.4, 5.9, 1.6 Hz, 1H; HRC=CR*H*), 4.28 – 4.24 (m, 1H; *H*R_3C), 4.05 (qq, *J* = 7.2, 3.7 Hz, 2H; COOC*H*$_2$$CH_3$), 3.87 – 3.83 (m, 1H; *H*R_3C), 3.81 (s, 3H; ROC*H*$_3$), 1.59 – 1.37 (m, 4H; R*H*$_2$C-C*H*$_2$R), 1.09 (t, *J* = 7.1 Hz, 3H; COOCH_2C*H*$_3$). ^{13}C NMR (100 MHz, $CDCl_3$ with TMS): *δ*=166.7,159.3, 156.2, 135.5, 133.1, 131.9, 131.5, 129.3, 113.2, 60.2, 55.4, 46.2, 39.0, 25.4, 24.8, 14.1. IR (cm^{-1}): 3051, 2940, 2873, 2834, 1691, 1600, 1507, 1460, 1440, 1399, 1349, 1244, 1213, 1173, 1147, 1122, 1054, 1047, 829, 793, 699, 671, 578, 528. elemental analysis calcd (%) for $C_{18}H_{20}O_3$: C 76.03, H 7.09, O 6.88; found: C 76.34, H 7.39.

Ethyl-3-(2-methoxyphenyl)bicyclo[2.2.2]octa-2,5-diene-2-carboxylate (23) Ethyl-3-bromobicyclo[2.2.2]octa-2,5-diene-2-carboxylate (1 equiv, 0.00175 mol, 0.45g) and 2-methoxyphenylboronic acid (1.2 equiv, 0.0021 mol, 0.32 g), were reacted at 60 °C for 20 hours according to general procedure A. After column chromatography (gradient 0-20% EtOAc in petroleum spirits) **23** (0.18 g, 40%) was obtained as a clear oil. R_f=0.58 (EtOAc/hexane 1:4);^{1}H NMR (400 MHz, $CDCl_3$ with TMS): *δ*=7.29 – 7.23 (m, 1H; Ar-*H*), 6.98 (dd, *J* = 7.7, 1.8 Hz, 1H; Ar-*H*), 6.91 – 6.87 (m, 2H; Ar-*H*), 6.47 (ddd, *J* = 7.4, 5.9, 1.7 Hz, 1H; *H*RC=CRH), 6.41 (ddd, *J* = 7.4, 5.9, 1.7 Hz, 1H; HRC=CR*H*), 4.27 – 4.23 (m, 1H; *H*R_3C), 4.00 – 3.90 (m, 2H; COOC*H*$_2$$CH_3$), 3.79 (s, 3H; ROC*H*$_3$), 3.75 – 3.72 (m, 1H; *H*R_3C), 1.61 – 1.55 (m, 2H; R*H*$_2$C-CH_2R), 1.43 – 1.35 (m, 2H; RH_2C-C*H*$_2$R), 0.92 (t, *J* = 7.1 Hz, 3H; COOCH_2C*H*$_3$).^{13}C NMR (100 MHz, $CDCl_3$ with TMS) *δ*=166.5, 156.5, 155.1, 135.1, 133.6, 133.5, 129.6, 128.8, 120.1, 110.7,

110.2, 59.9, 55.7, 45.5, 38.3, 25.5, 24.4, 13.8. IR (cm^{-1}): 3059, 2936, 2869, 2837, 1691, 1638, 1592, 1489, 1432, 1367, 1343, 1264, 1242, 1215, 1181, 1118, 1068, 1051, 1027, 837, 793, 748, 705, 675, 629, 554, 501. elemental analysis calcd (%) for $C_{18}H_{20}O_3$: C 76.03, H 7.09, O 16.88; found: C 75.61, H 7.41.

Ethyl-3-(naphthalen-2-yl)bicyclo[2.2.2]octa-2,5-diene-2-carboxylate (24) Ethyl-3-bromobicyclo[2.2.2]octa-2,5-diene-2-carboxylate (1 equiv, 0.00194 mol, 0.5 g) in and 2-naphtylboronic acid (1.2 equiv, 0,00233 mol, 0.40 g), were reacted at 60 °C for 19 hours according to general procedure A. After column chromatography (gradient 0-10% EtOAc in petroleum spirits) and then a second time to automated flash column chromatography (gradient 0-5% EtOAc in Hexane) **24** (0.39g, 66%) was obtained as a slightly yellow oil. R_f=0.6 (EtOAc/hexane 1:4);^{1}H NMR (400 MHz, $CDCl_3$): δ=7.84 – 7.76 (m, 3H; Ar-*H*), 7.68 (q, *J* = 0.9 Hz, 1H; Ar-*H*), 7.49 – 7.44 (m, 2H; Ar-*H*), 7.33 (dd, *J* = 8.4, 1.8 Hz, 1H; Ar-*H*), 6.53 (ddd, *J* = 7.6, 6.1, 1.6 Hz, 1H; *H*RC=CRH), 6.46 (ddd, *J* = 7.4, 5.9, 1.6 Hz, 1H; HRC=CR*H*), 4.32 (dh, *J* = 6.8, 1.5 Hz, 1H; HR_3C), 4.06 – 3.94 (m, 3H; HR_3C and $COOCH_2CH_3$), 1.66 – 1.58 (m, 2H; RH_2C-CH_2R), 1.52 – 1.41 (m, 2H; RH_2C-CH_2R), 0.94 (t, *J* = 7.1 Hz, 3H; $COOCH_2CH_3$). ^{13}C NMR (100 MHz, $CDCl_3$): δ=166.6, 156.3, 137.2, 135.5, 133.2, 133.1, 132.9, 132.8, 128.2, 127.8, 127.2, 126.4, 126.3, 126.2, 126.1, 60.2, 46.2, 39.1, 25.1, 24.9, 14.0. IR (cm^{-1}): 3053, 2943, 2859, 1690, 1597, 1502, 1444, 1370, 1344, 1265, 1243, 1215, 1186, 1148, 1119, 1093, 1036, 1066, 1019, 965, 893, 855, 815, 795, 747, 699, 674, 626, 476. elemental analysis calcd (%) for $C_{21}H_{20}O_2$: C 82.86, H 6.62, O 10.51; found: C 82.97, H 6.75.

Ethyl-3-(naphthalen-1-yl)bicyclo[2.2.2]octa-2,5-diene-2-carboxylate (25) Ethyl-3-bromobicyclo[2.2.2]octa-2,5-diene-2-carboxylate (1 equiv, 0.0039 mol, 1.0 g) and 1-naphtylboronic acid (1.2 equiv, 0,004689 mol, 0.802 g) were reacted at 60 °C for 20 hours according to general procedure A. After column chromatography (gradient 5% EtOAc in petroleum spirits) and then a second time to automated flash column chromatography (gradient 2% EtOAc in petroleum spirits) **25** (0.78 g, 66%) was obtained as a mixtures of rotamers and as a slightly yellow oil. R_f=0.6 (EtOAc/hexane 1:4);^{1}H NMR (400 MHz, $CDCl_3$): δ =7.86 – 7.73 (m, 3H), 7.57 (ddd, *J* = 7.9, 1.8, 0.8 Hz, 0.5H), 7.52 – 7.35 (m, 5.4H), 7.21 (dd, *J* = 7.1, 1.2 Hz, 0.4H), 7.12 (dd, *J* = 7.0, 1.2 Hz, 1H), 6.65 – 6.46 (m, 3H), 4.44 (dq, *J* = 4.9, 2.4 Hz, 0,4H), 4.40 (ddd, *J* = 6.7, 2.5, 1.1 Hz, 1H), 3.88 – 3.69 (m, 3.6H), 3.62 (qd, *J* = 7.1, 4.6 Hz, 1H), 1.82 – 1.45 (m, 7H), 0.59 (t, *J* = 7.1 Hz, 3H), 0.32 (t, *J* = 7.1 Hz, 1.2H). ^{1}H NMR (DMSO-d_6 at 130 °C): δ = 7.97 – 7.78 (m, 2H; Ar-*H*), 7.52 – 7.41 (m, 4H; Ar-*H*), 7.16 (dd, *J* = 7.0, 1.2 Hz, 1H; Ar-*H*), 6.60 (qdd, *J* = 7.3, 5.6, 1.7 Hz, 2H; *H*RC=CR*H*), 4.31 (dq, *J* = 4.2, 2.1 Hz, 1H; HR_3C), 3.80 – 3.64 (m, 3H; HR_3C and $COOCH_2CH_3$), 1.73 – 1.60 (m, 2H; RH_2C-CH_2R), 1.52 – 1.46 (m, 2H; RH_2C-CH_2R), 0.65 – 0.58 (m, 3H; $COOCH_2CH_3$). ^{13}C NMR (100 MHz, $CDCl_3$): δ=165.9, 157.1, 139.4, 138.8, 135.7, 135.7, 134.4, 133.6, 133.4, 133.0, 131.4, 128.4, 128.3, 127.4, 127.3, 126.0, 125.9, 125.8, 125.7, 125.2, 125.1, 123.9, 123.6, 59.9, 47.0, 46.3, 38.3, 38.3, 25.6, 24.9, 24.6, 13.5, 13.0. IR (cm^{-1}): 3054, 2948, 2870, 1693, 1637, 1601, 1506, 1445, 1395, 1370, 1345, 1262, 1232, 1217, 1175, 1147, 1124, 1067, 1036, 1016, 848, 883, 794, 774, 738, 704, 676, 649, 631, 573, 474. elemental analysis calcd (%) for $C_{21}H_{20}O_2$: C 82.86, H 6.62, O 10.51; found: C 83.20, H 6.89.

Ethyl-4'-methoxy-[1,1'-biphenyl]-2-carboxylate (30) Ethyl 2-bromobenzoate (1 equiv, 0.0014 mol, 0.215 g) and 4-methoxyphenylboronic acid (1.45 equiv, 0.002 mol, 0.310 g) were reacted at 80 °C for 2 hours, according to general procedure A. After column chromatography (25% DCM in hexane **30** (0.27 g, 77%) was obtain as a colorless oil. Data consistent with literature[2]

^{1}H NMR (400 MHz, $CDCl_3$): δ= 7.77–7.79 (m, 1H), 7.46-7.51 (m, 1H), 7.33-7.38 (m, 2H), 7.22-7.26 (m, 2H), 6.90–6.94 (m, 2H), 4.09 (q, *J* = 7.2 Hz, 2H), 3.83 (s, 3H), 1.04 (t, *J* = 7.2 Hz, 3H). ^{13}C NMR (100 MHz, $CDCl_3$): δ =168.9, 158.9, 141.9, 133.8, 131.3, 131.0, 130.6, 129.6, 129.5, 126.8, 113.5, 113.4, 77.4, 77.0, 76.7, 60.9, 55.3, 13.8.

Ethyl-2'-methoxy-[1,1'-biphenyl]-2-carboxylate (31) Ethyl 2-bromobenzoate (1 equiv, 0.0014 mol, 0.215 g) and 2-methoxyphenylboronic acid (1.45 equiv, 0.002 mol, 0.310 g) were reacted at 80 °C for 2 hours, according to general procedure A. After column chromatography (25% DCM in hexane) **31** (0.27 g, 78%) was obtain as a colorless oil. Data consistent with literature[3]

^{1}H NMR (400 MHz, $CDCl_3$): δ = 7.87 (d, *J*=7.8 Hz, 1H). 7.51–7.55 (m, 1H) 7.31–7.41 (m, 3H), 7.23–7.25 (m, 1H,), 7.01–7.06 (m, 1H), 6.88-6.90 (d, *J* = 8.1 Hz, 1H), 4.08 (q, *J* = 7.1 Hz, 2H), 3.71 (s, 3H), 1.04 (t, *J* = 7.1 Hz, 3H). ^{13}C NMR (100 MHz, $CDCl_3$): δ=168.3, 156.1, 138.7, 131.9, 131.4, 131.2, 130.8, 129.9, 129.4, 128.7, 127.1, 120.6, 110.0, 77.4, 77.0, 76.7, 60.5, 55.2, 13.7.

Ethyl-2-(naphthalen-2-yl)benzoate (32) Ethyl 2-bromobenzoate (1 equiv, 0.0014 mol, 0.215 g) and 2-naphtylboronic acid (1.45 equiv, 0.002 mol, 0.343 g) were reacted at 80 °C for 2 hours according to general procedure A. After column chromatography (25% DCM in hexane) **32** (0.29 g, 75%) was obtain as a colorless oil. Data consistent with literature[4]

^{1}H NMR (400 MHz, $CDCl_3$): δ= 7.79-7.89 (m, 5H), 7.43-7.58 (m, 6H), 4.09 (q, *J* = 7.2 Hz, 2H), 0.91 (t, *J* = 7.2 Hz, 3H). ^{13}C NMR (100 MHz, $CDCl_3$): δ = 168.6, 142.2, 138.9, 133.0, 132.3, 131.2, 131.1, 130.8, 129.7, 127.9, 127.5, 127.2, 127.1, 126.9, 126.7, 126.0, 125.8, 77.2, 76.8, 76.5, 60.8, 13.5.

Ethyl-2-(naphthalen-1-yl)benzoate (33) Ethyl 2-bromobenzoate (1 equiv, 0.0014 mol, 0.215 g) and 1-naphtylboronic acid (1.45 equiv, 0.002 mol, 0.343 g) were reacted at 80 °C for 2 hours according to general procedure A. After column chromatography (25% DCM in hexane) **33** (0.284 g, 74%) was obtain as a colorless oil. Data consistent with literature[4]

^{1}H NMR (400 MHz, $CDCl_3$): δ=8.03-8.05 (m, 1H). 7.85-7.89 (m, 2H), 7.59-7.63 (m, 1H), 7.31-7.54 (m, 7H), 3.71-3.87 (m, 2H), 0.55 (t, $_J$ = 7.2 Hz, 3H). ^{13}C NMR (100 MHz, $CDCl_3$): δ=167.6, 140.9, 139.7, 133.0, 132.0, 131.8, 131.5, 131.3, 129.9, 127.9, 127.4, 127.2, 125.8, 125.7, 125.5, 125.4, 124.9, 77.2, 76.8, 76.5, 60.4, 12.9.

Table S.2.1 Screening of reaction conditions and product formation of ethyl-3-phenylbicyclo[2.2.2]octa-2,5-diene-2-carboxylate analyzed by NMR.

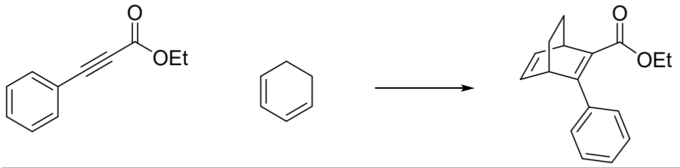

**Entry**	**Solvent**	**Diene equivalent**	**Temperature (°C)**	**Time (h)**	**Product formation[a] (%)**
**1**	chlorobenzene	2	110	18	19
				45	19
**2**	toluene	1.5	160	4	0
				23	14
				42	14
**3**	toluene	4.5	160	4	13
				23	17
				42	17
**4**	toluene	9	160	4	(-)
				23	22
				42	27
				78	30
**5**	neat	9	160	21	23
				48	26
				72	29

[a] (-) The quality of the NMR spectra was too low to analyze the product formation.

2. Numbering and structures of compounds

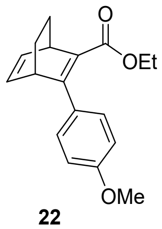

22

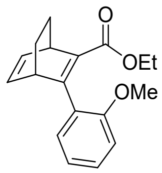

23

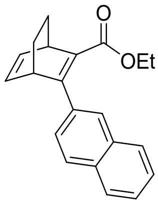

24

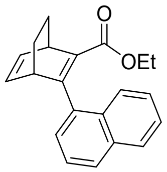

25

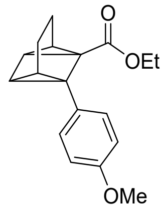

26

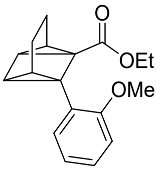

27

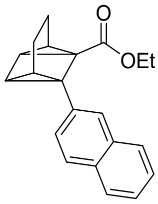

28

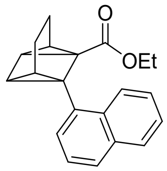

29

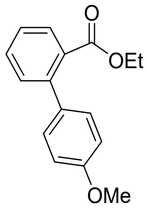

30

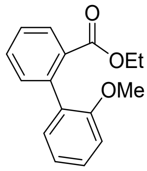

31

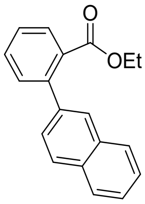

32

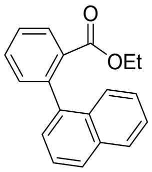

33

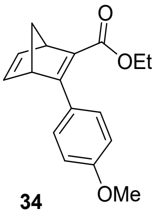

34

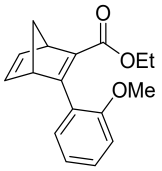

35

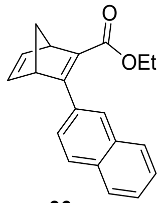

36

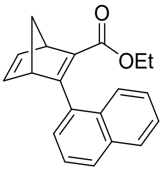

37

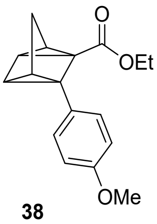

38

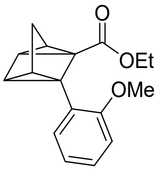

39

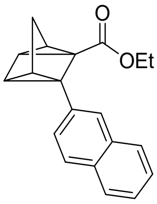

40

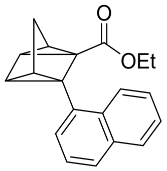

41

Figure S2.1. Structure of relevant compounds and their numbering.

3. NMR spectroscopy

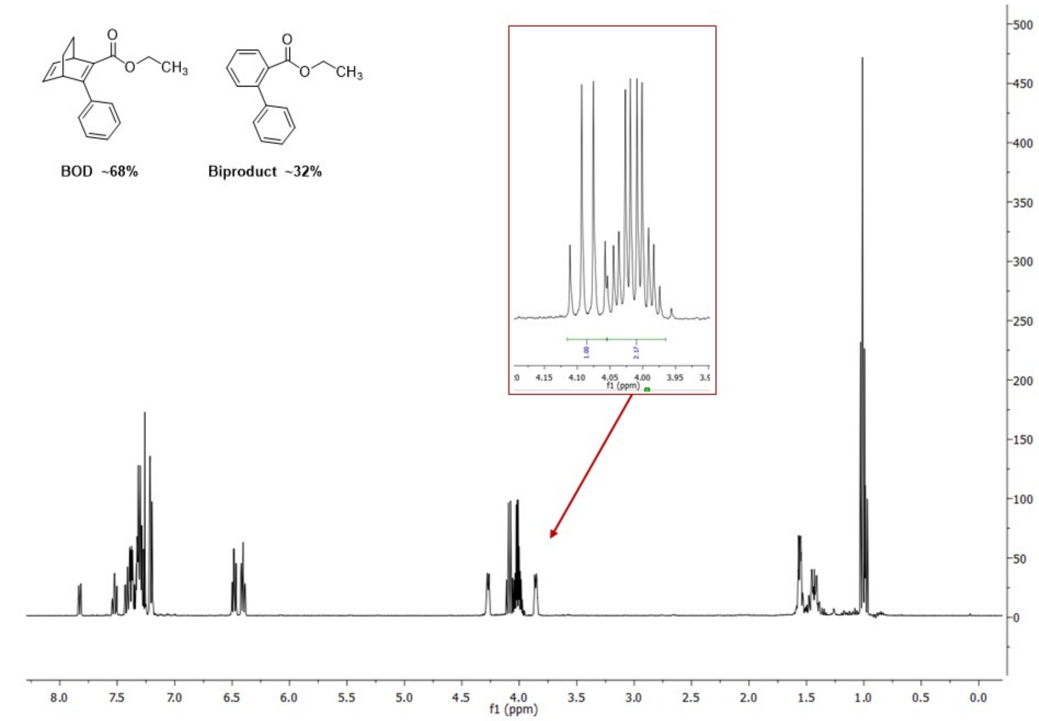

Figure S3.1 ^{1}H-NMR of ethyl-3-phenylbicyclo[2.2.2]octa-2,5-diene-2-carboxylate and ethyl [1,1'-biphenyl]-2-carboxylate.

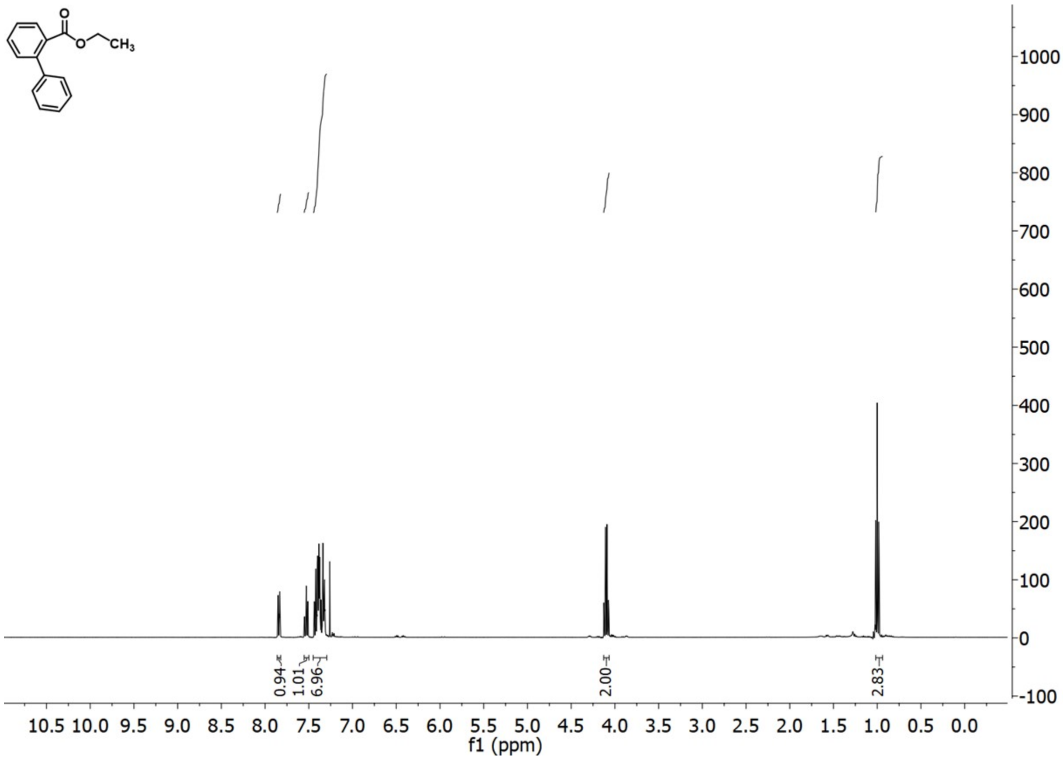

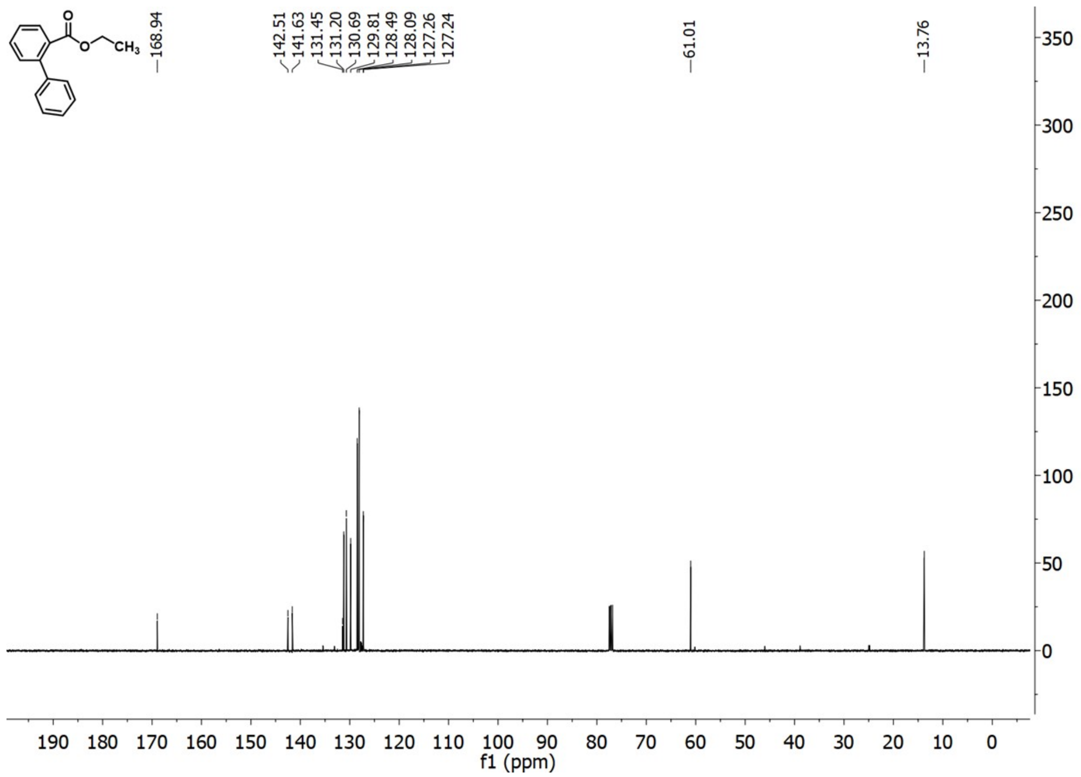

Figure S3.2 ^{1}H-NMR and ^{13}C-NMR of ethyl [1,1'-biphenyl]-2-carboxylate.

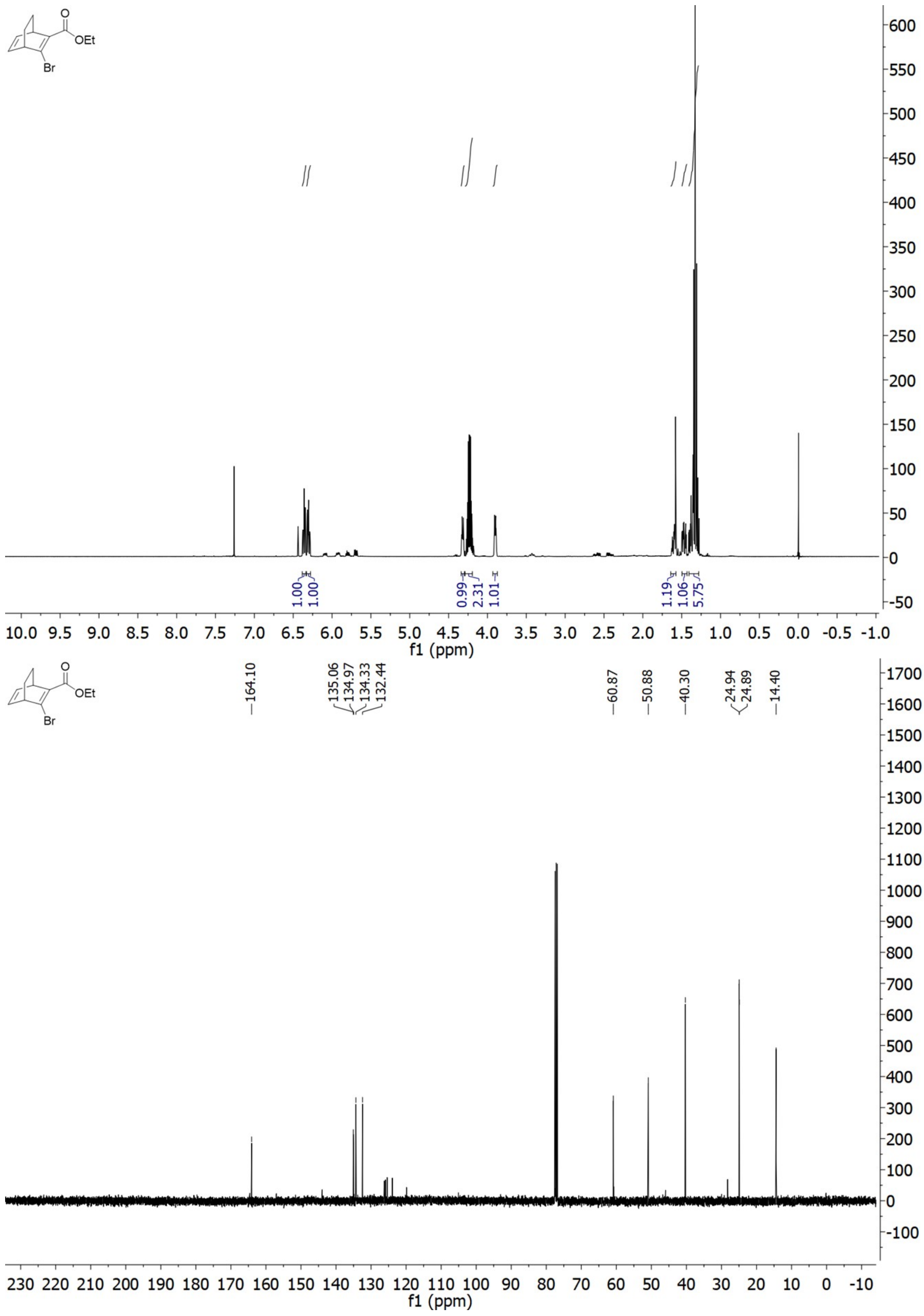

Figure S3.3 ^{1}H-NMR and ^{13}C-NMR of ethyl-3-bromopropilate.

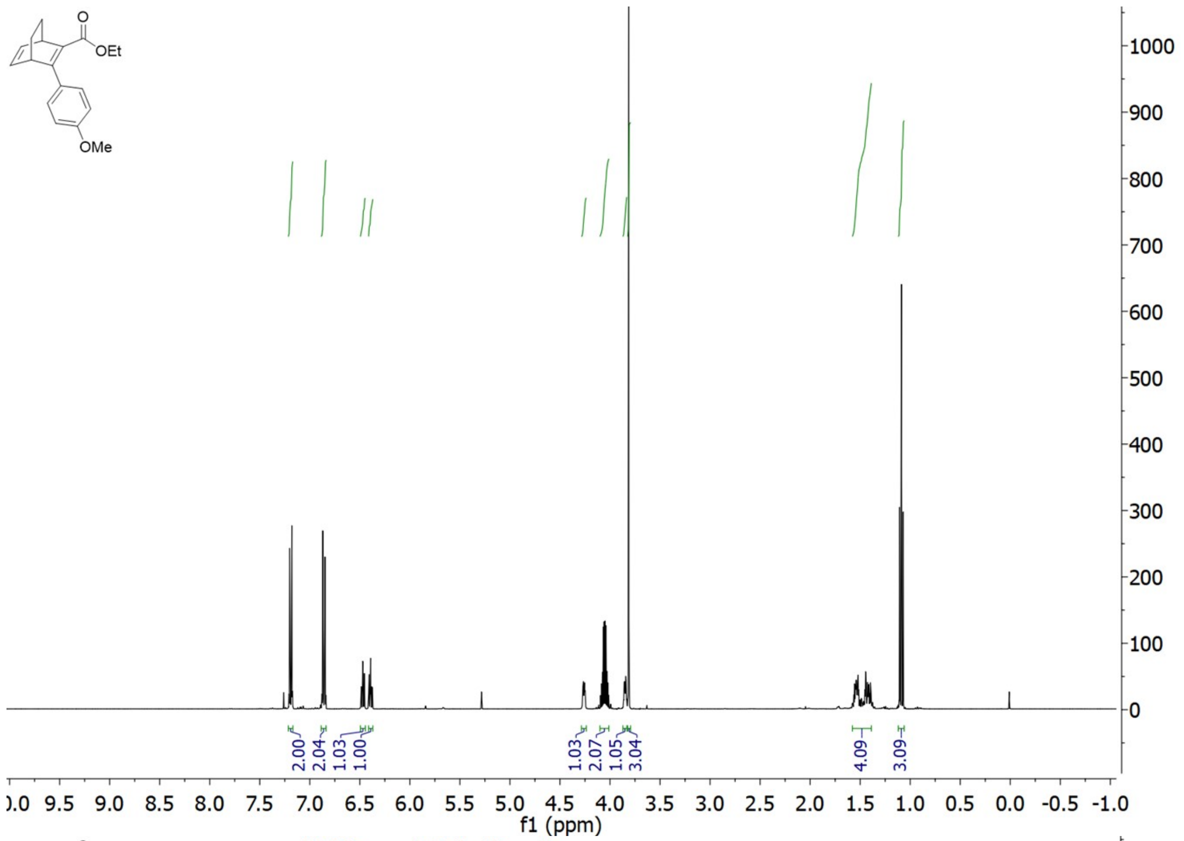

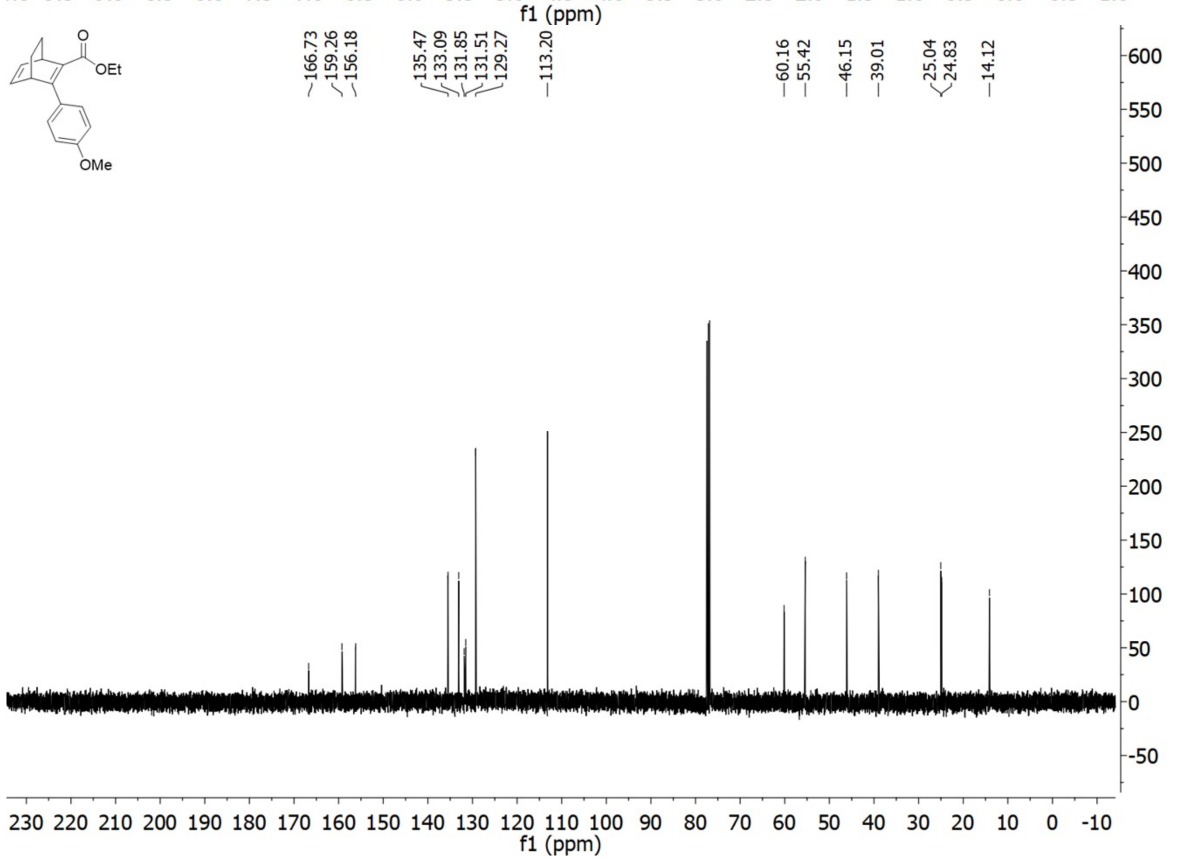

Figure S3.4 ^{1}H-NMR and ^{13}C-NMR of Ethyl-3-(4-methoxyphenyl)bicyclo[2.2.2]octa-2,5-diene-2-carboxylate (**22**)

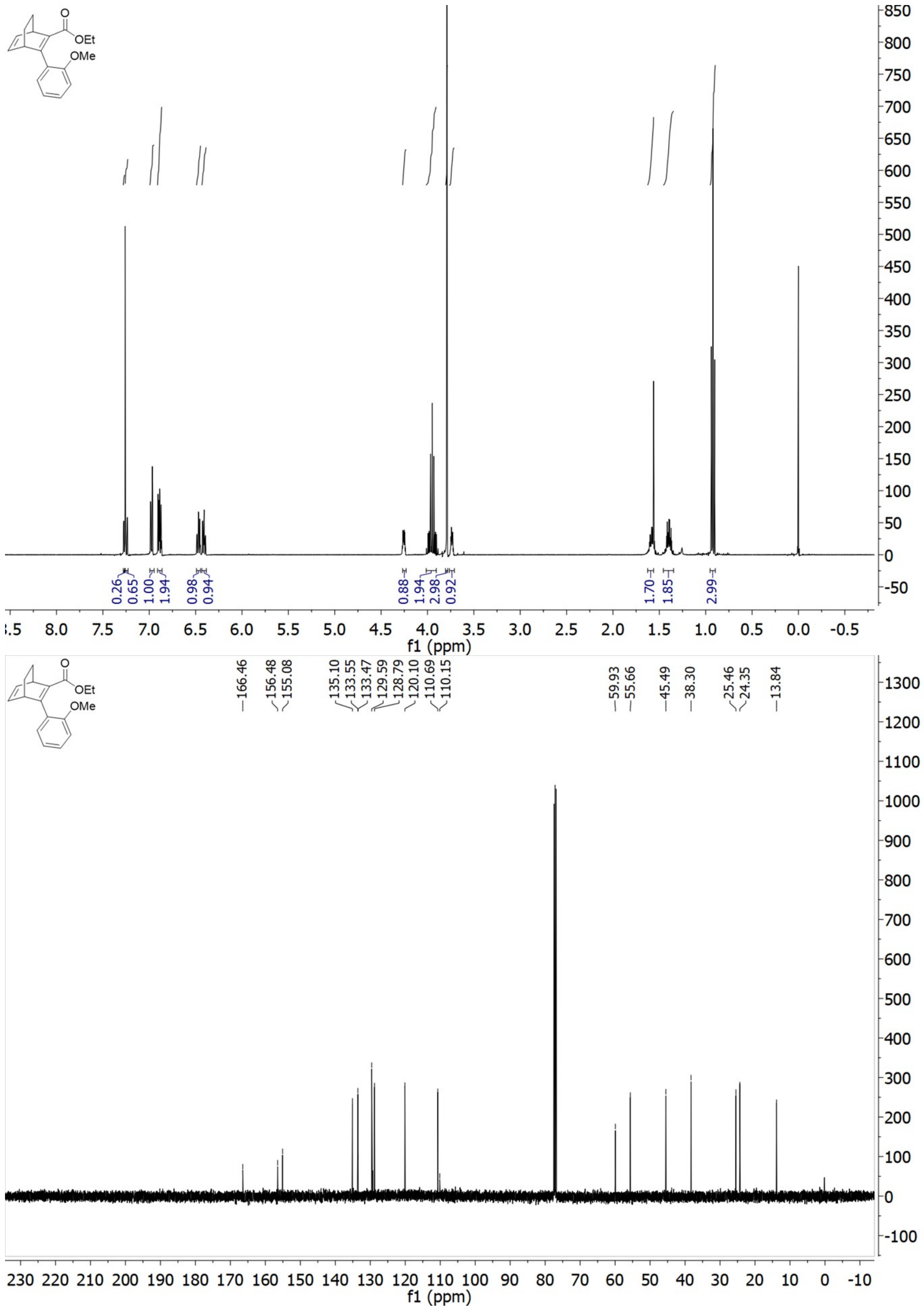

Figure S3.5 ^{1}H-NMR and ^{13}C-NMR of Ethyl-3-(1-methoxyphenyl)bicyclo[2.2.2]octa-2,5-diene-2-carboxylate (**23**).

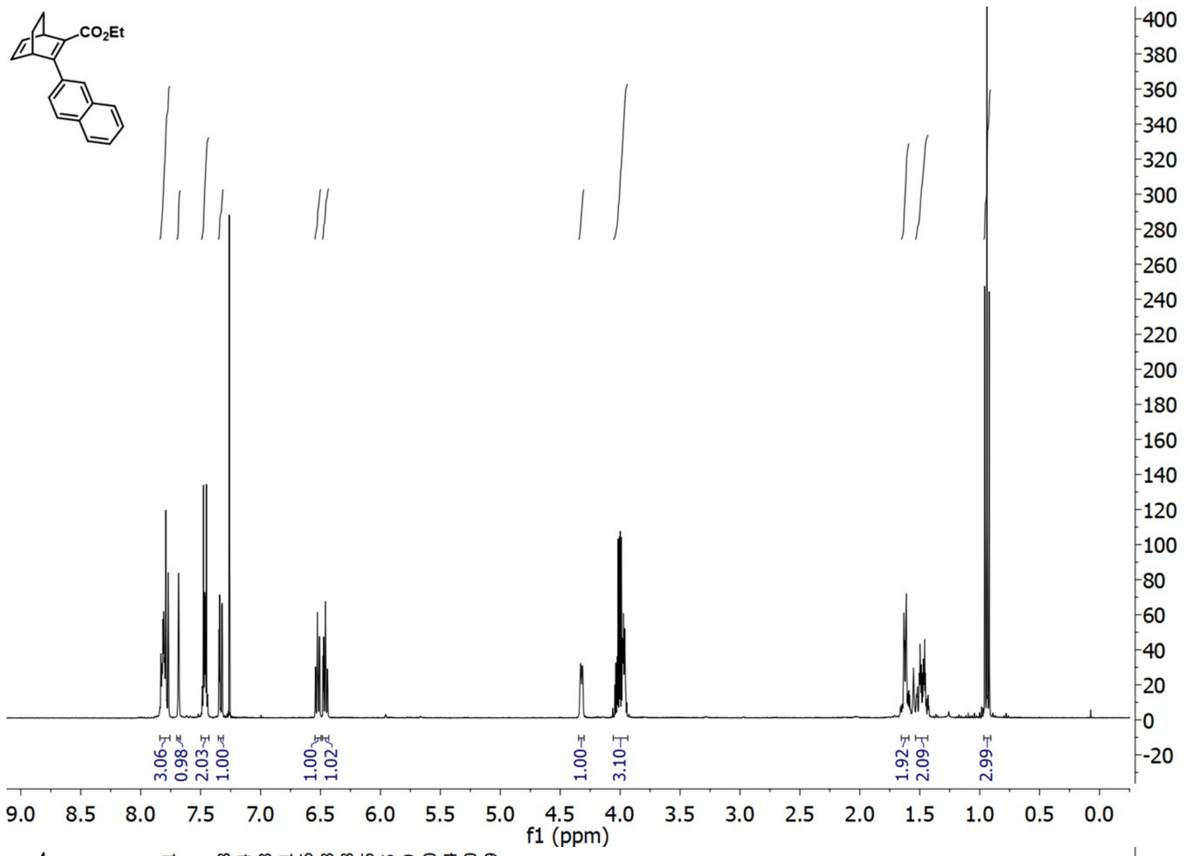

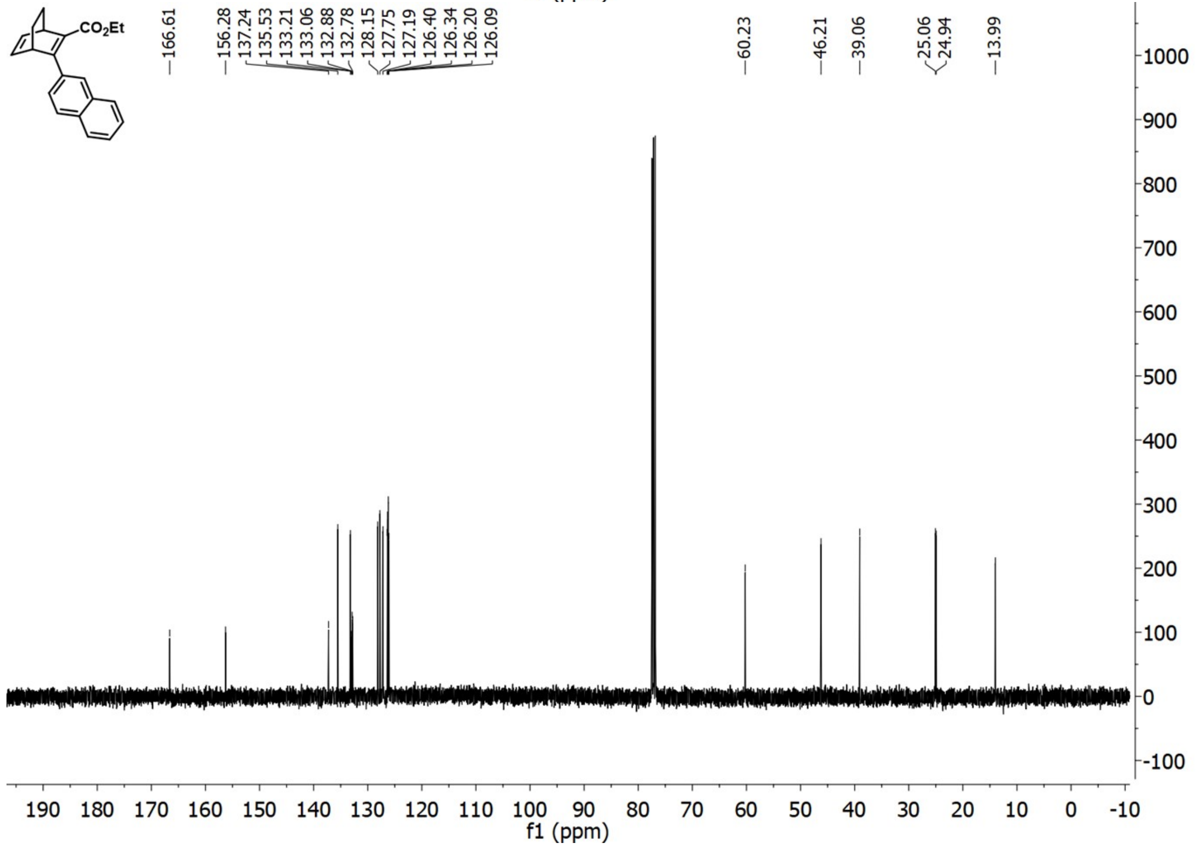

Figure S3.6 ^{1}H-NMR and ^{13}C-NMR of Ethyl-3-(naphthalen-2-yl)bicyclo[2.2.2]octa-2,5-diene-2-carboxylate (**24**).

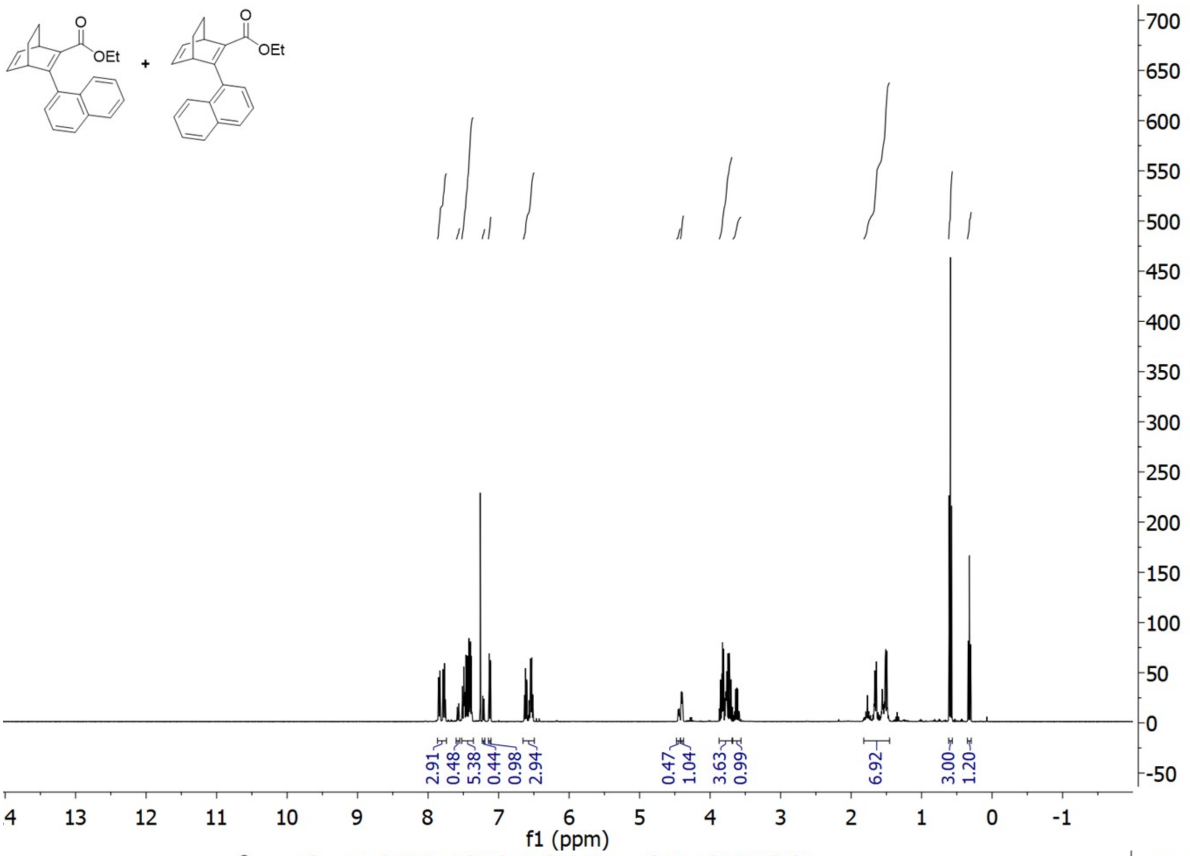

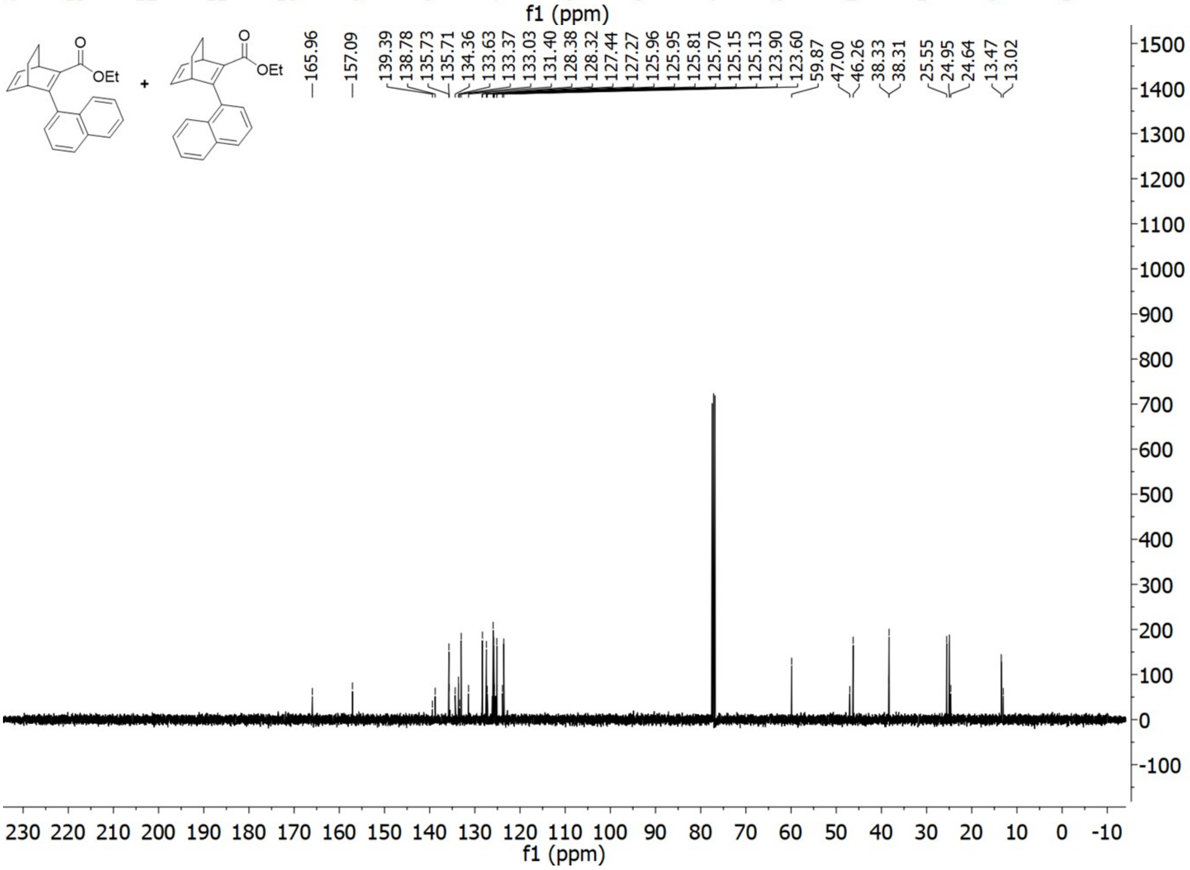

Figure S3.7 ^{1}H-NMR and ^{13}C-NMR of Ethyl-3-(naphthalen-1-yl)bicyclo[2.2.2]octa-2,5-diene-2-carboxylate (**25**) at room temperature.

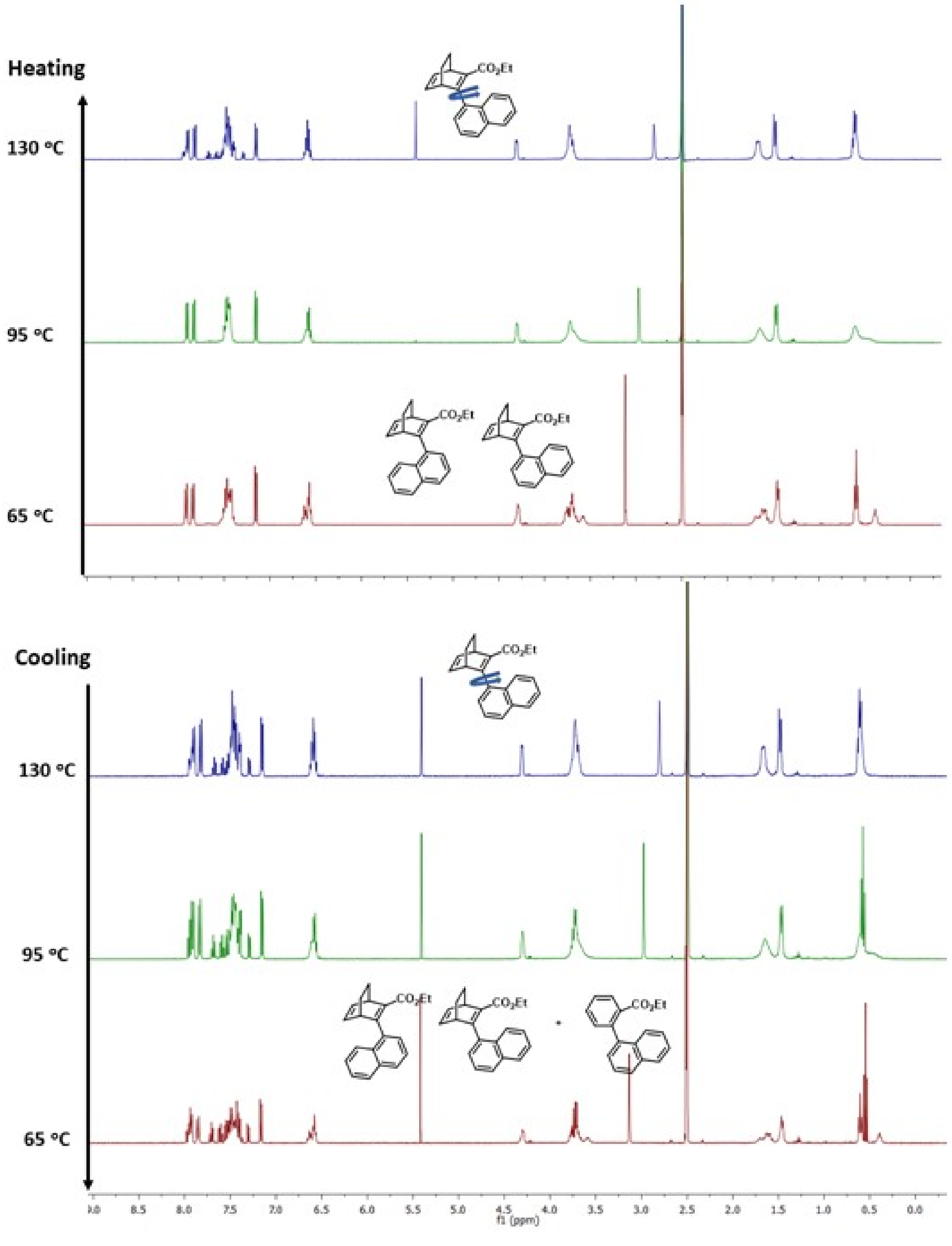

Figure S3.8 ^{1}H-NMR of ethyl-3-(naphthalen-1-yl)bicyclo[2.2.2]octa-2,5-diene-2-carboxylate (**25**) at 3 different temperatures. a) heating up the sample b) cooling down the sample.

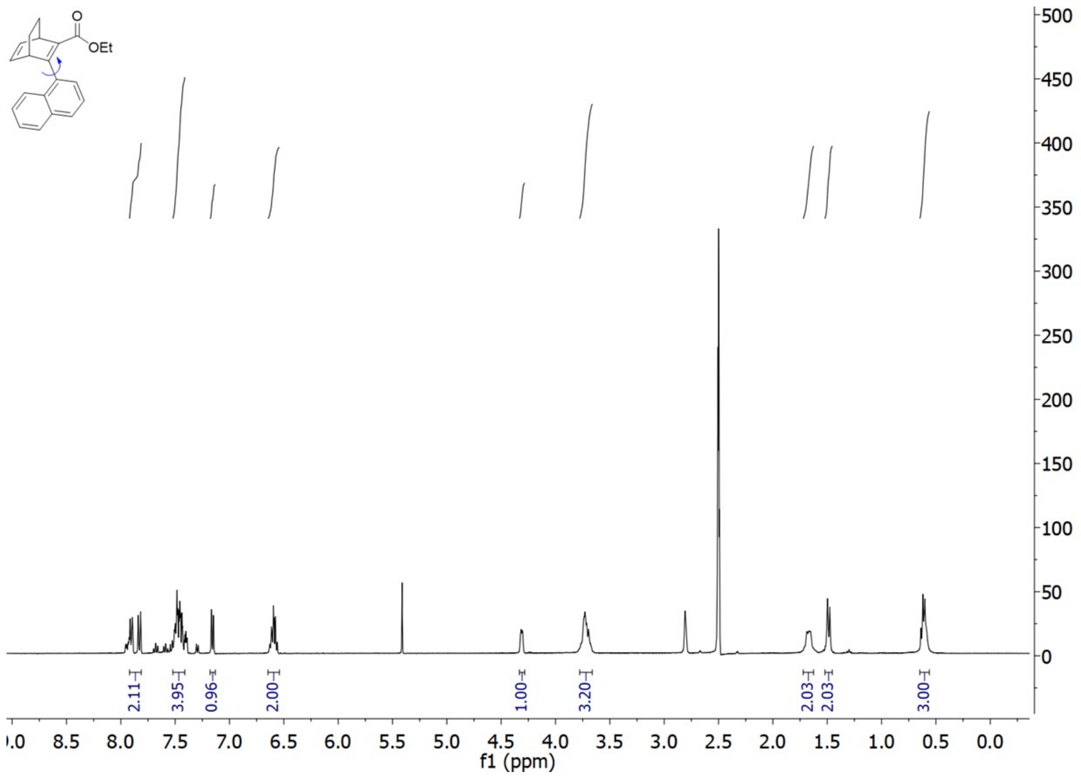

Figure S3.9 ^{1}H-NMR of ethyl-3-(naphthalen-1-yl)bicyclo[2.2.2]octa-2,5-diene-2-carboxylate (**25**) at 130°C.

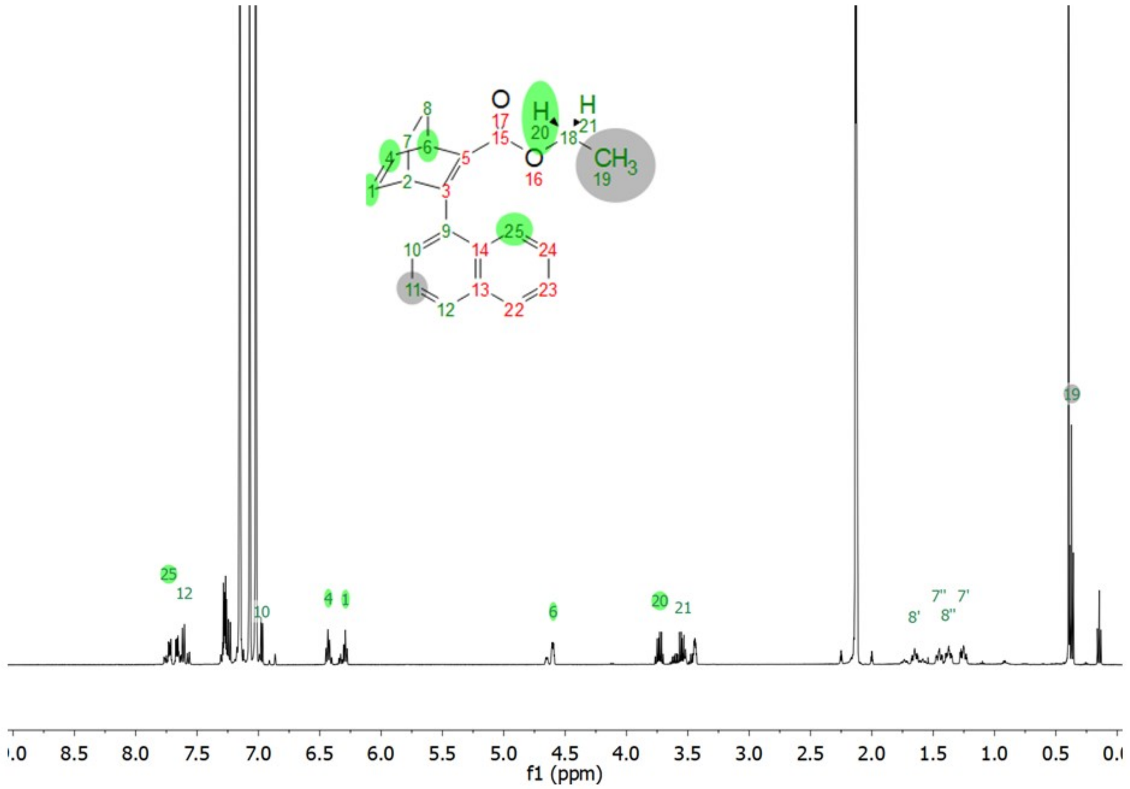

Figure S3.10 ^{1}H-NMR of **25** in toluene-d_8 with relevant protons assigned.

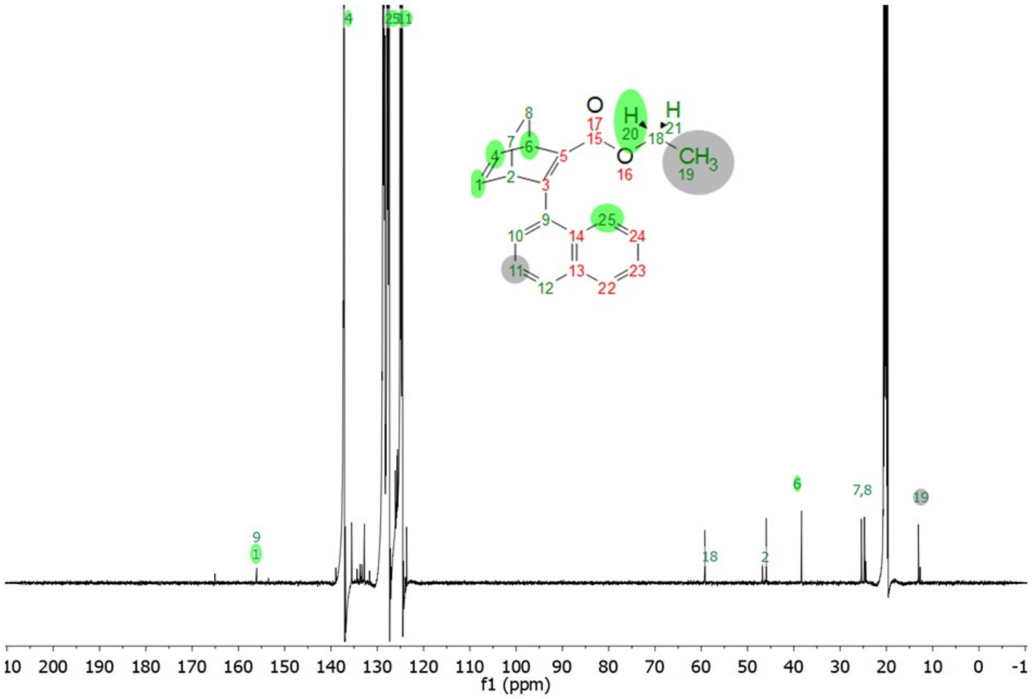

Figure S3.11 ^{13}C-NMR of **25** in toluene-d$_8$ with relevant protons assigned.

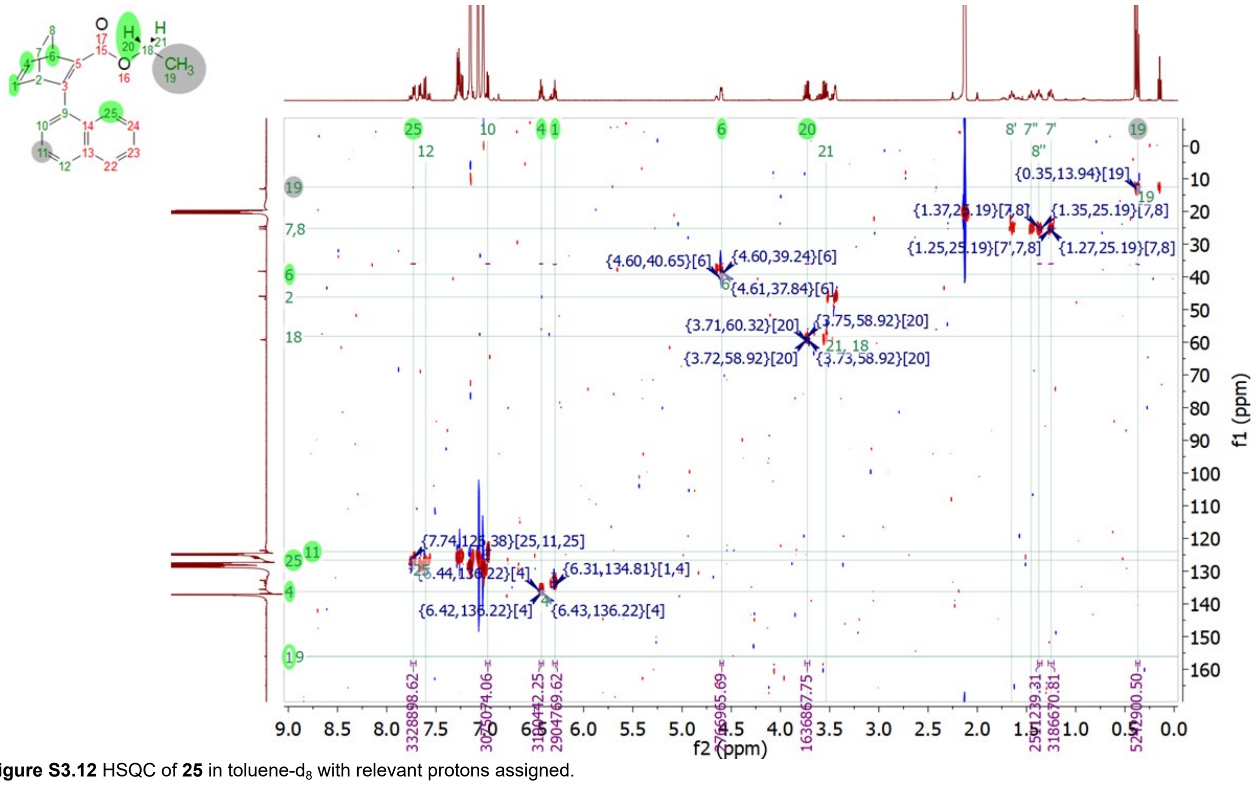

Figure S3.12 HSQC of **25** in toluene-d$_8$ with relevant protons assigned.

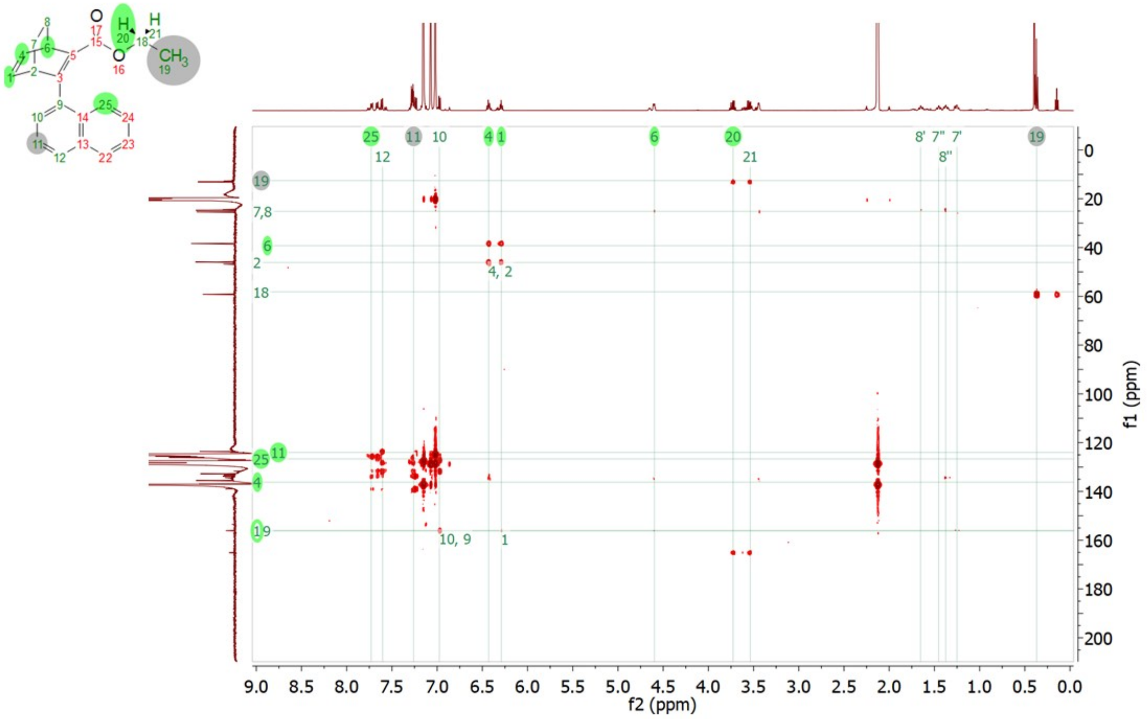

Figure S3.13 HMBC of **25** in toluene-d_8 with relevant protons assigned.

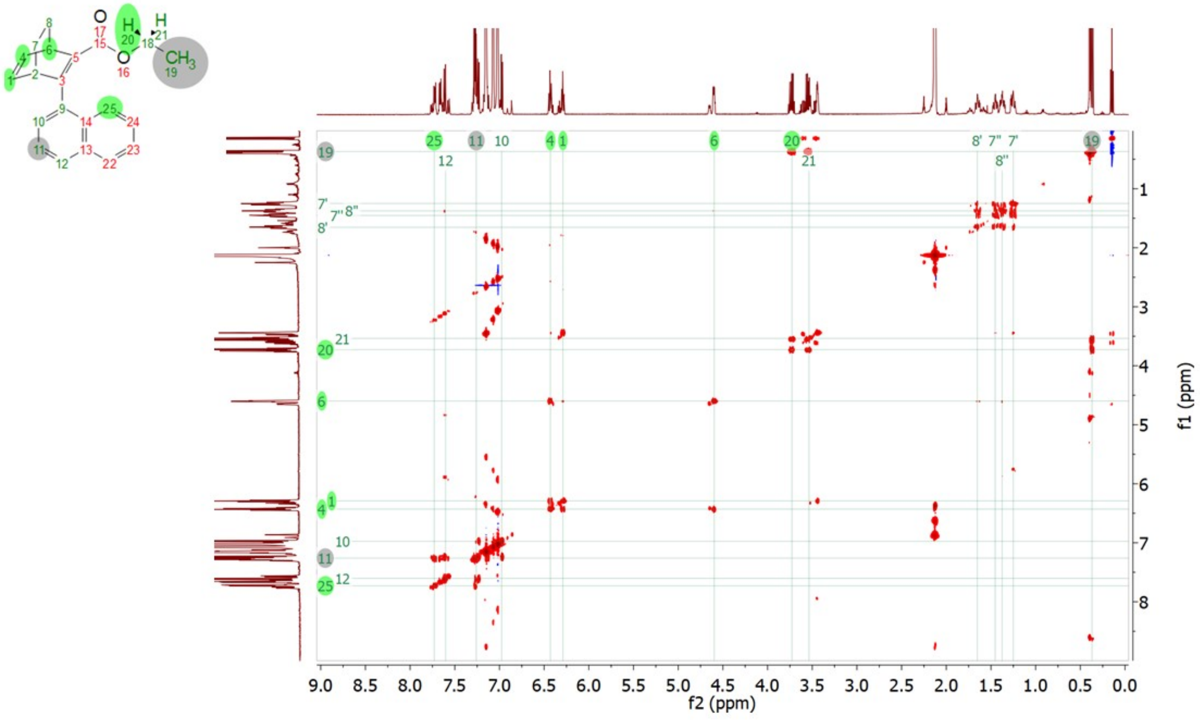

Figure S3.14 COSY spectra of **25** in toluene-d8 with relevant protons assigned.

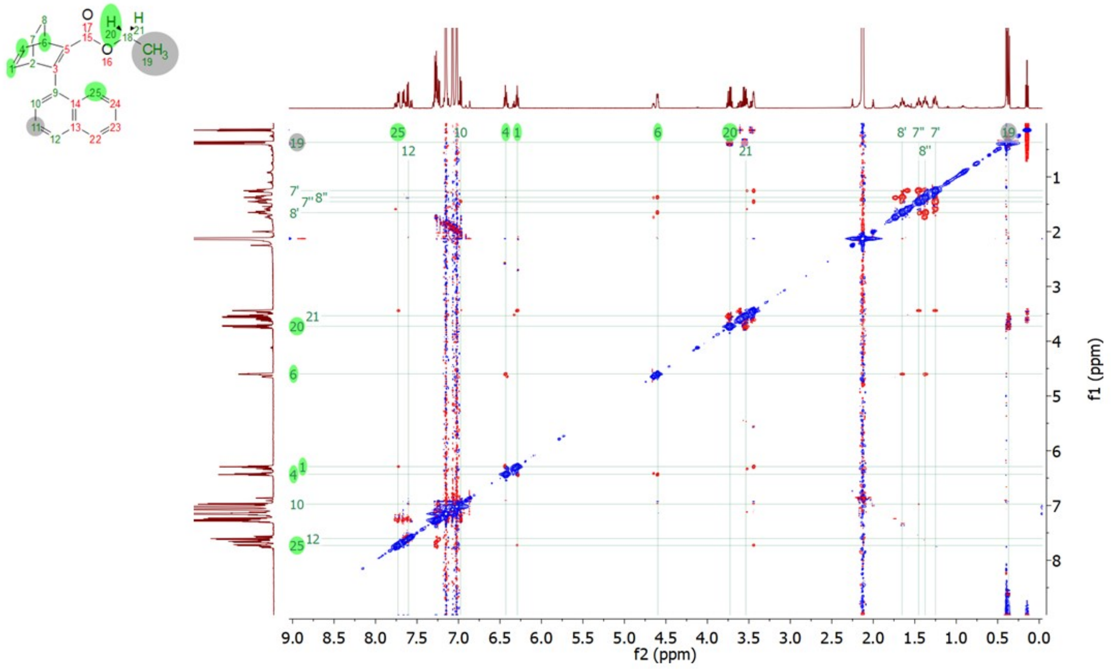

FigureS3.15 Full NOESY of **25** in toluene--d_8 with relevant protons assigned.

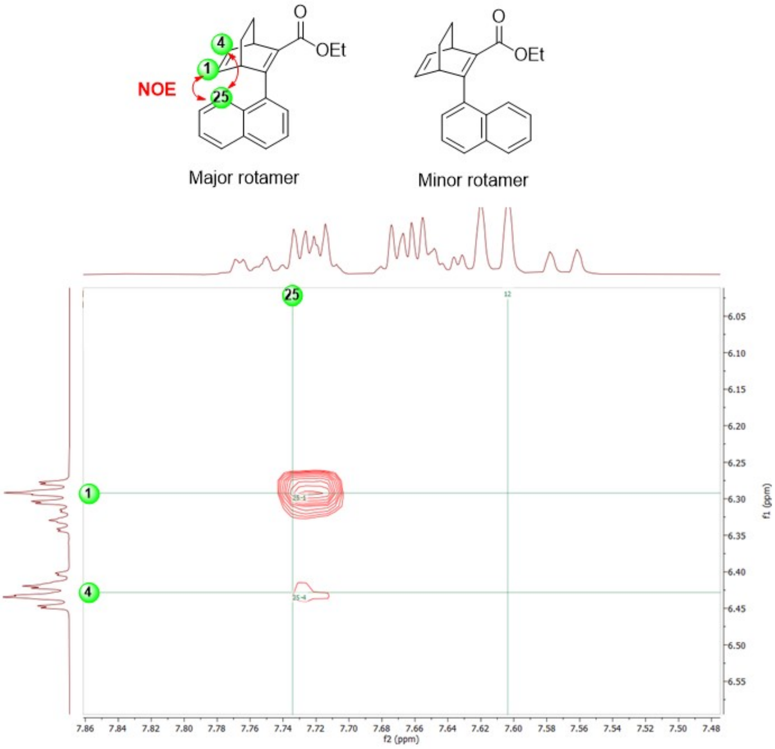

Figure S3.16 Parts of NOESY spectra of **25** in toluene-d8 showing NOE between protons 25 and 1 and 25 and 4 in the major rotamer. No NOE between these protons were obtained in the minor rotamer.

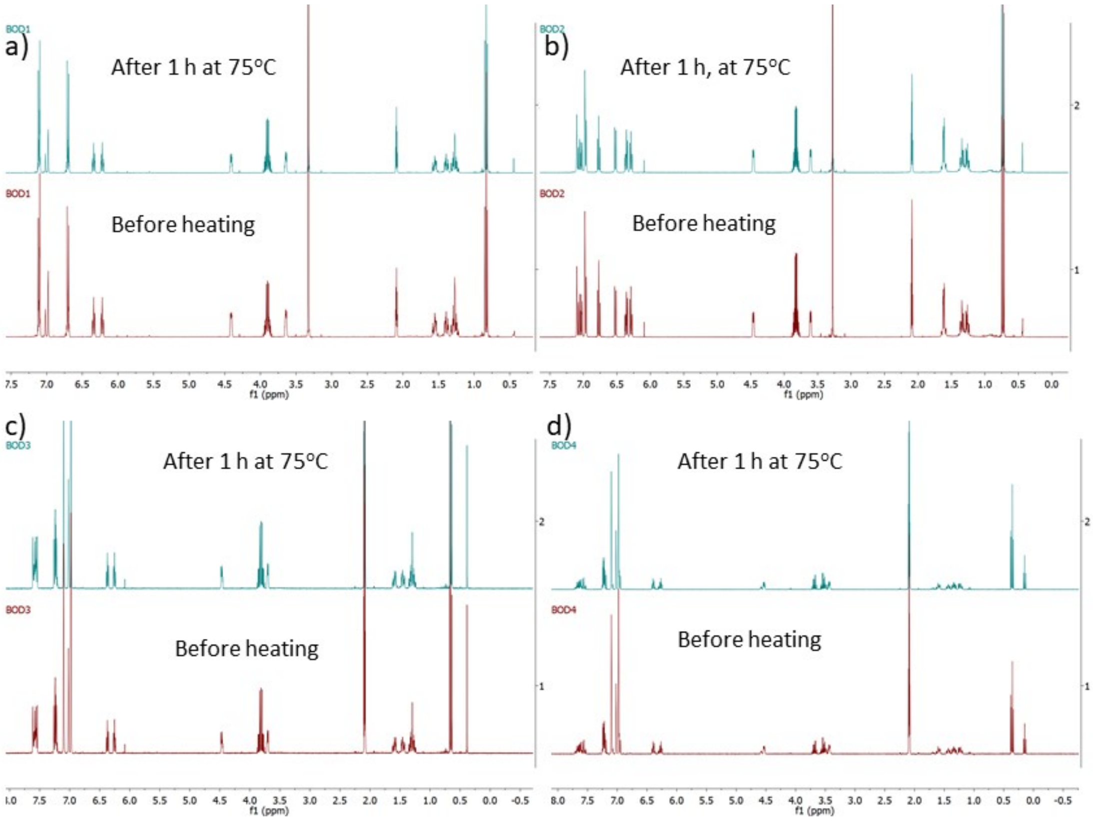

Figure S3.17 ^{1}H-NMR before and after heating up to 75 ^{o}C a) **22**, b) **23**, c) **24** and d) **25**.

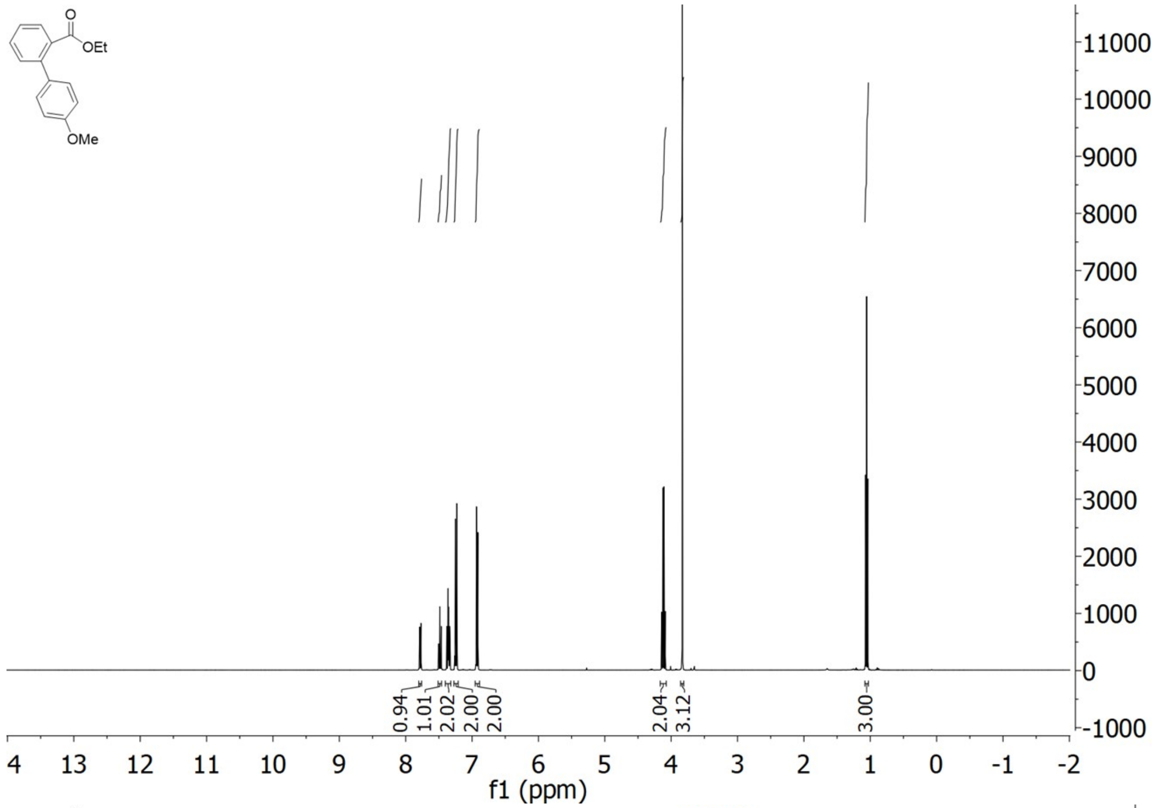

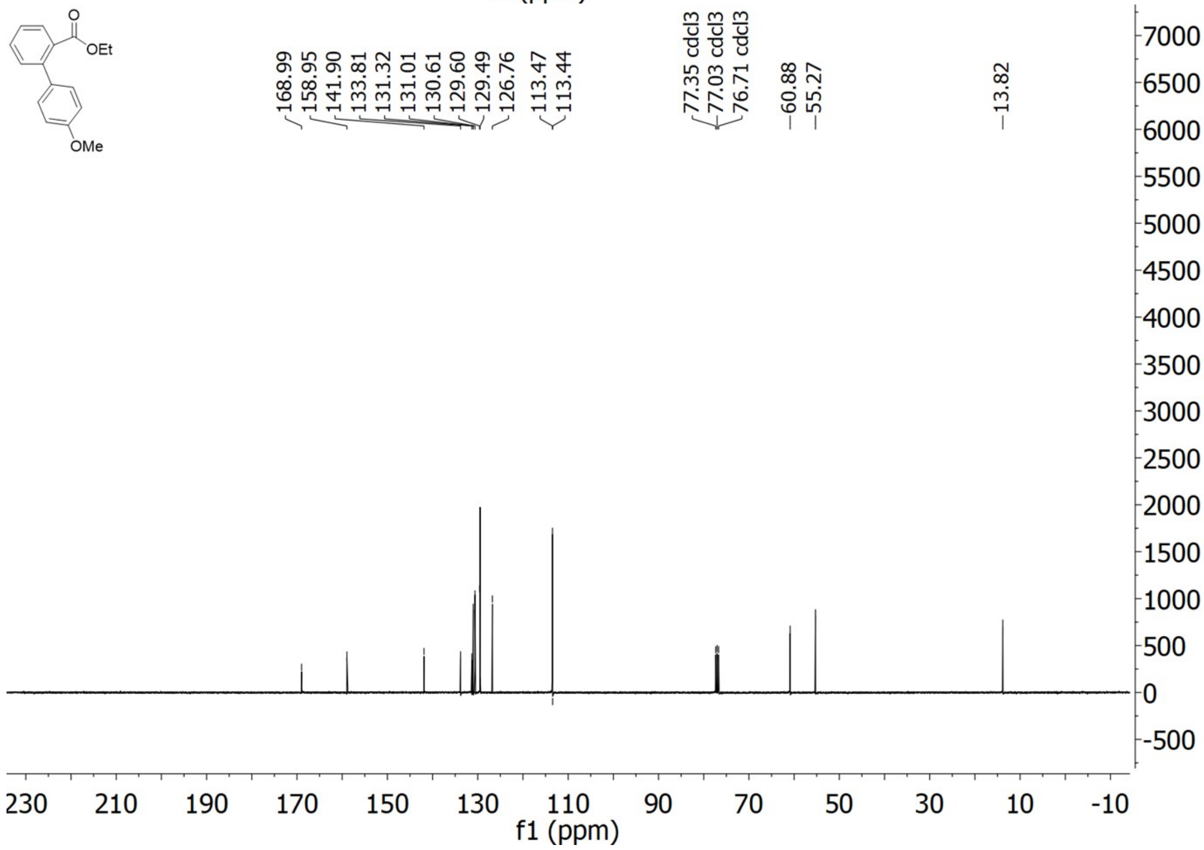

Figure S3.18 ^{1}H-NMR and ^{13}C-NMR of ethyl-4'-methoxy-[1,1'-biphenyl]-2-carboxylate (**30**).

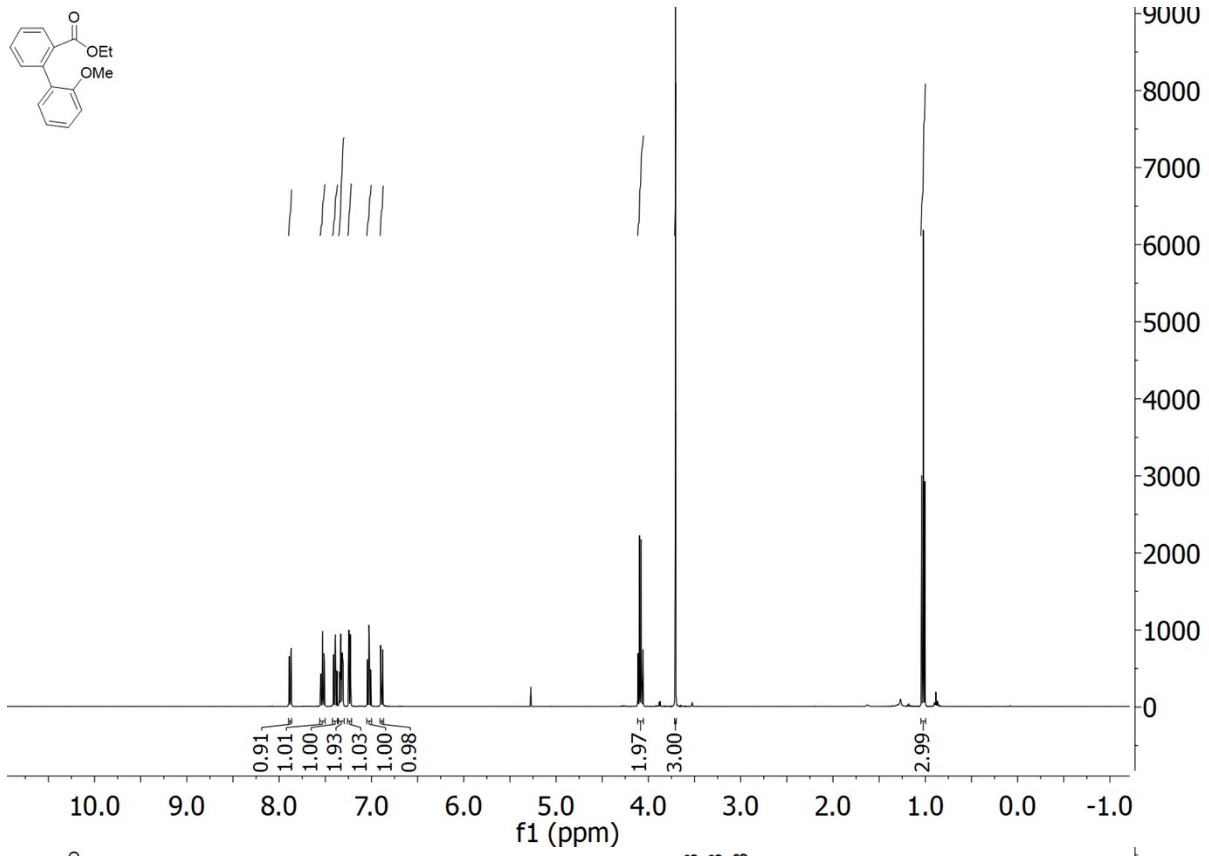

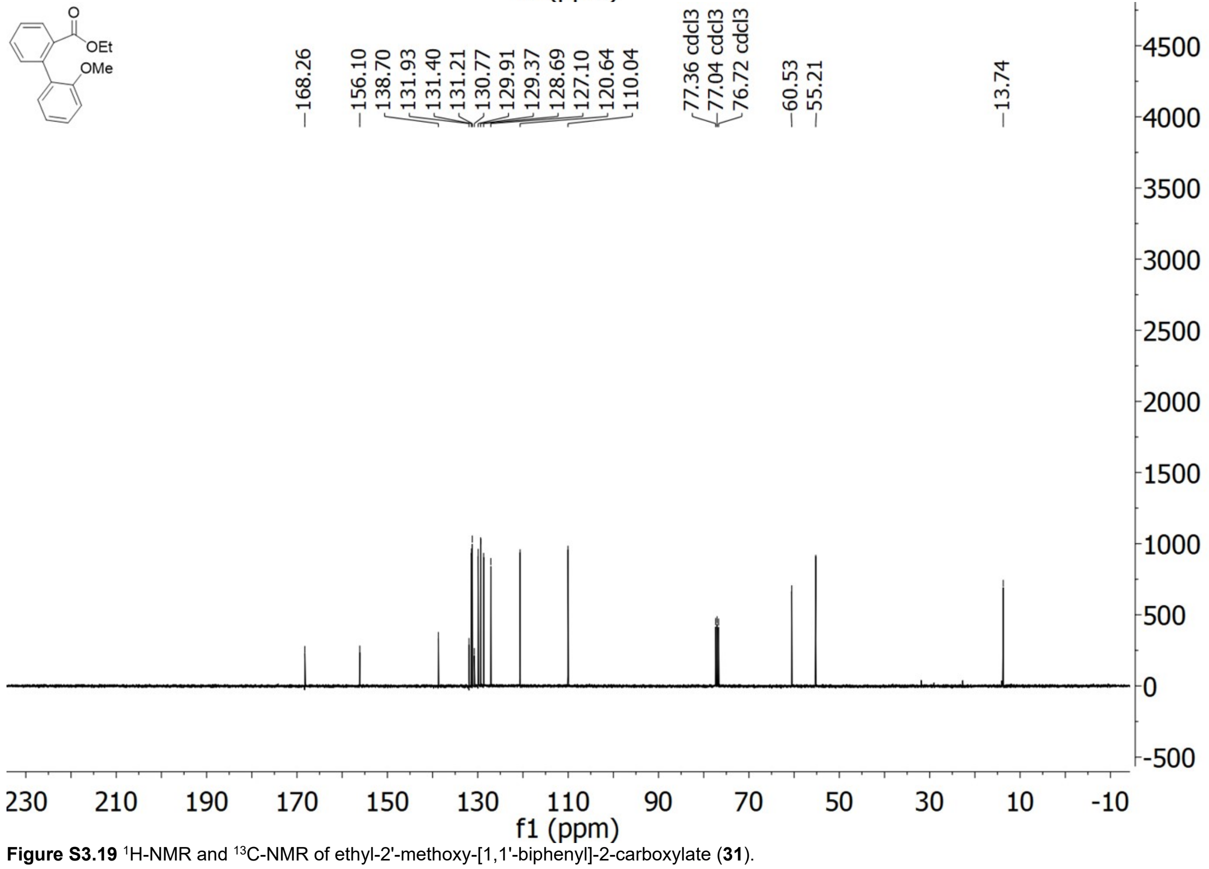

Figure S3.19 ^{1}H-NMR and ^{13}C-NMR of ethyl-2'-methoxy-[1,1'-biphenyl]-2-carboxylate (**31**).

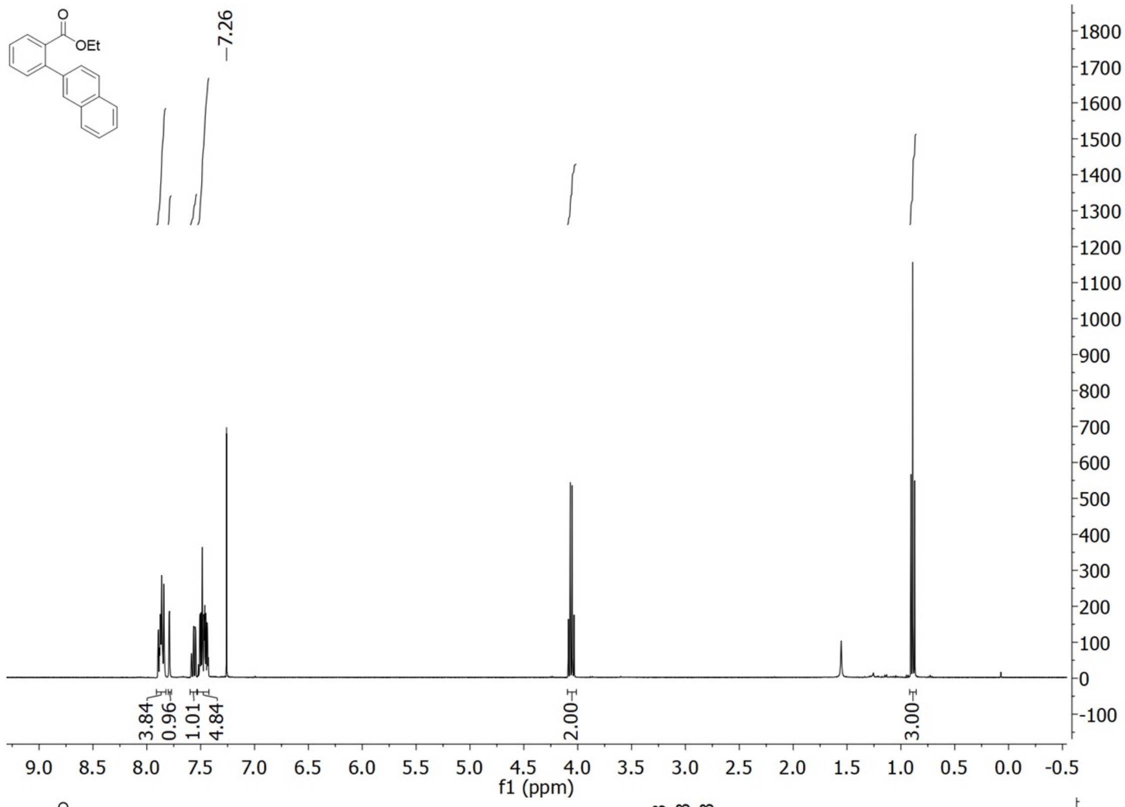

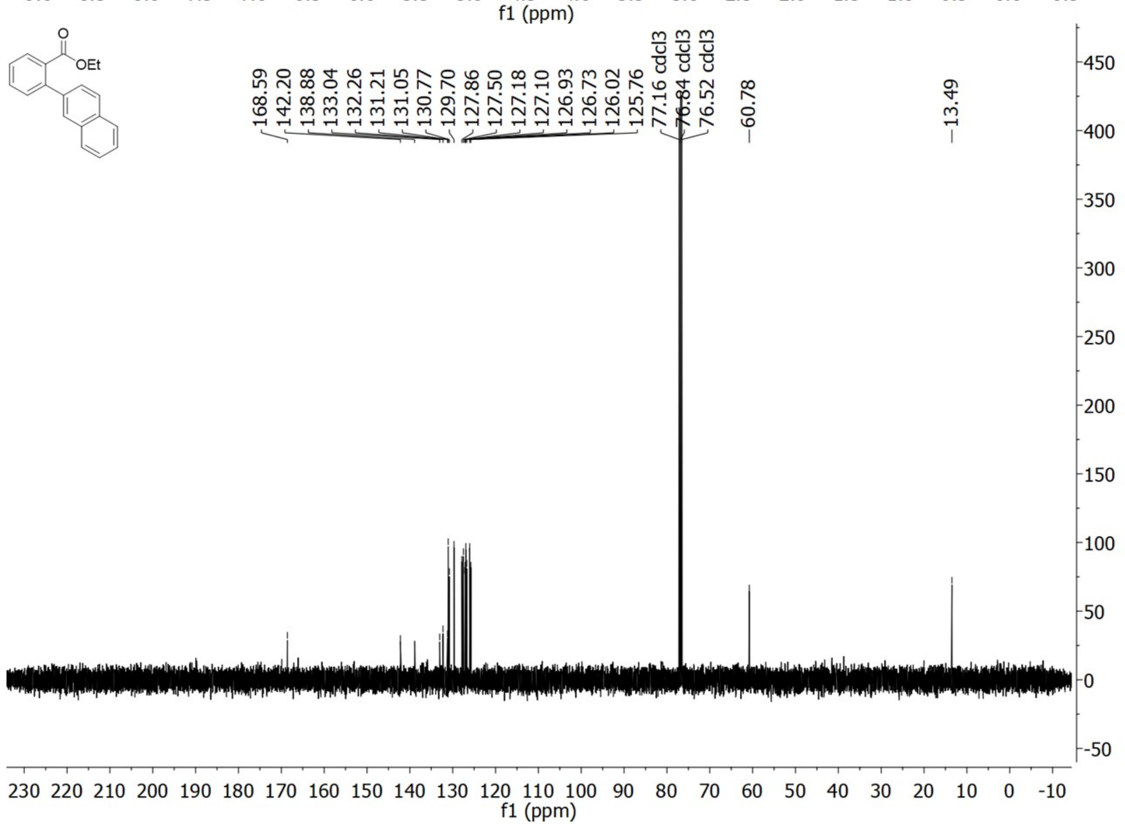

Figure S3.20 ^{1}H-NMR and ^{13}C-NMR of Ethyl-2-(naphthalen-2-yl)benzoate (**32**).

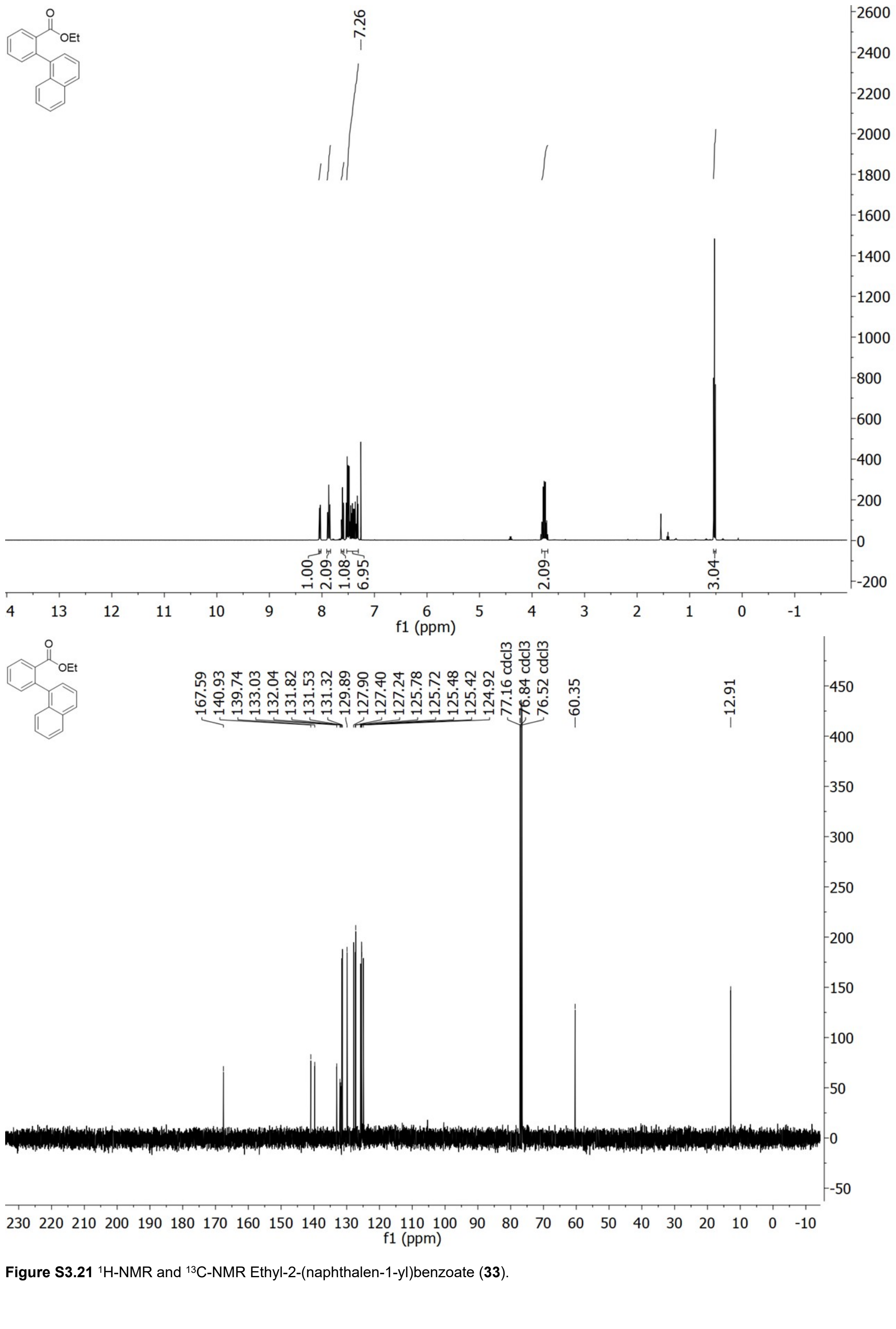

Figure S3.21 ^{1}H-NMR and ^{13}C-NMR Ethyl-2-(naphthalen-1-yl)benzoate (**33**).

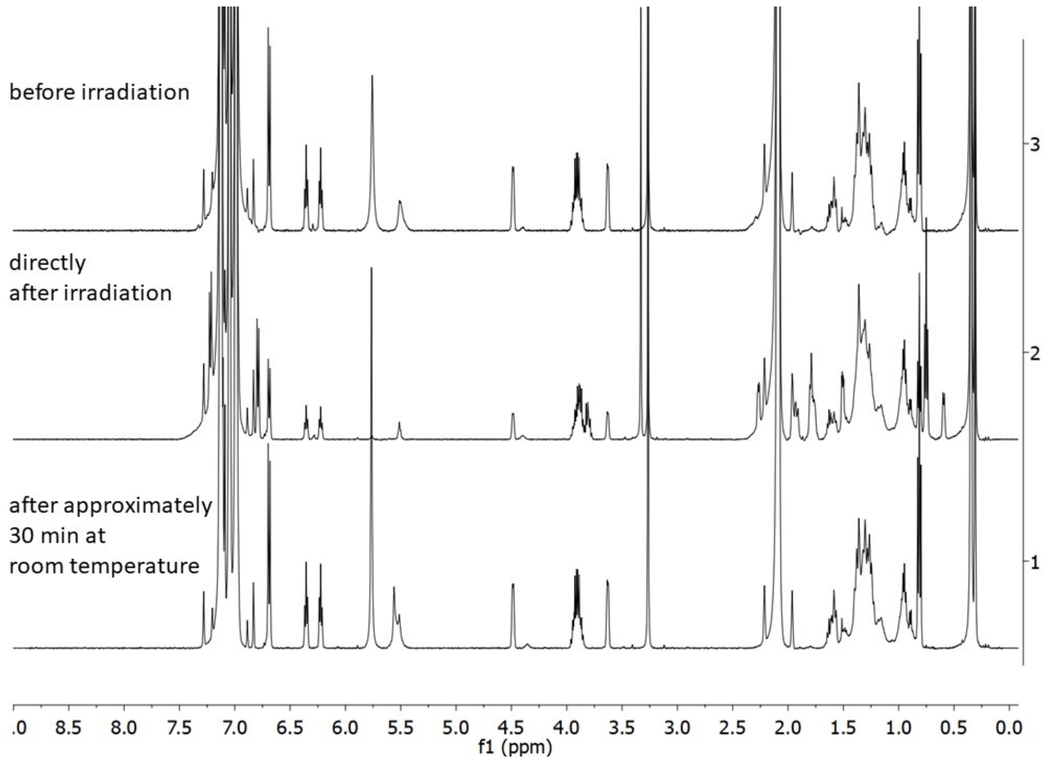

Figure S3.22 ^{1}H-NMR of **22** in toluene-d_8 recorded at -20°C. Spectra from before irradiation (BOD), directly after irradiation (BOD and TCO) and after the sample was kept at room temperature to facilitate the back conversion (BOD).

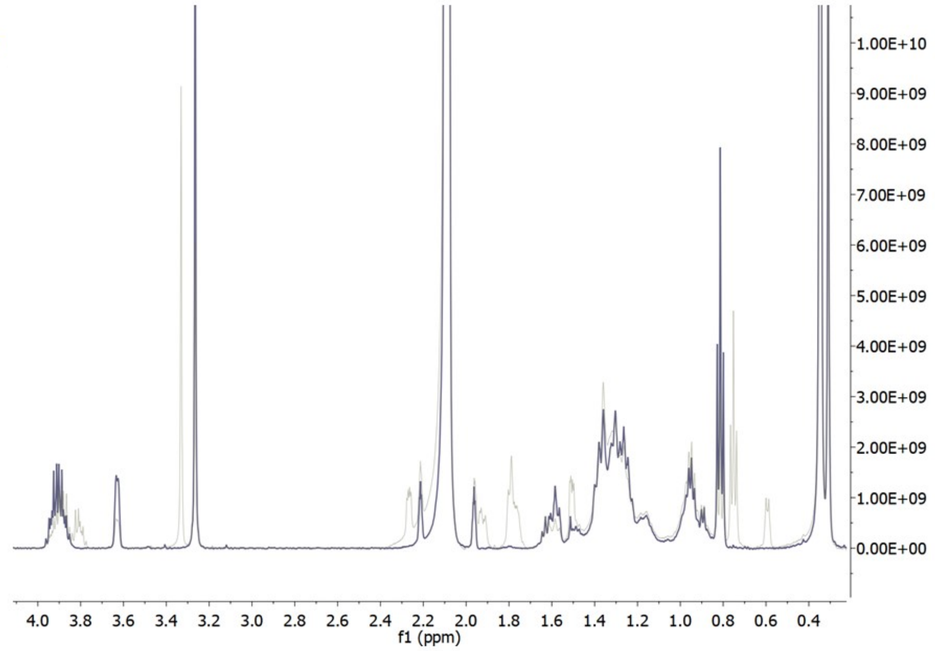

Figure S3.23 Superimposed ^{1}H-NMR spectra of before and after irradiation of BOD **22** to TCO **26**. Selected signals from BOD are presented in blue and from TCO in grey.

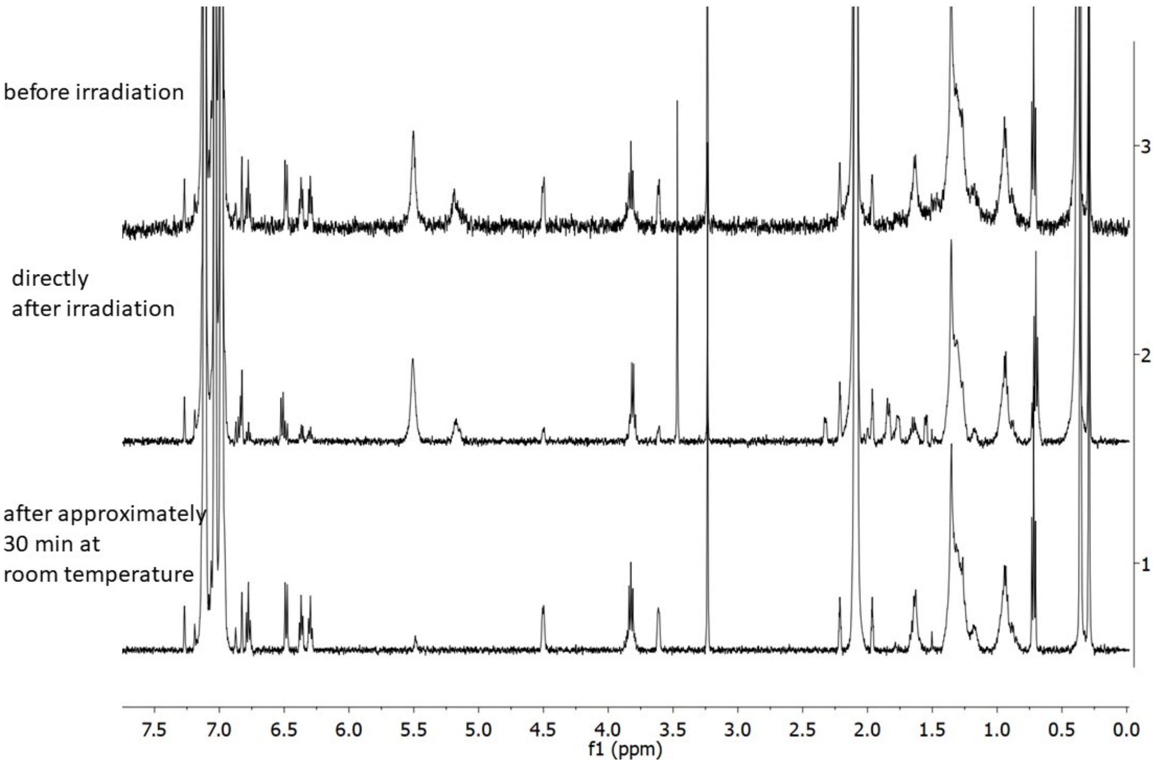

Figure S3.24 ^{1}H-NMR of **23** in toluene-d_8 recorded at -20°C. Spectra from before irradiation (BOD), directly after irradiation (BOD and TCO) and after the sample was kept at room temperature to facilitate the back conversion (BOD).

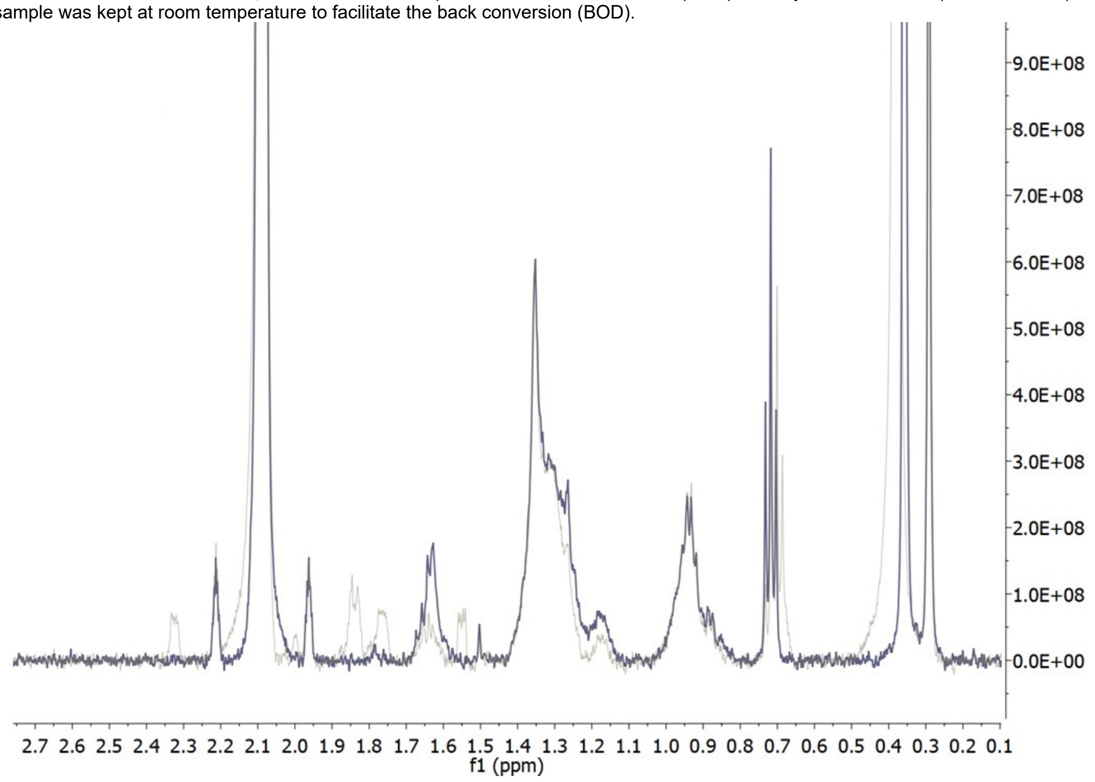

Figure S3.25 Superimposed ^{1}H-NMR spectra of before and after irradiation of BOD **23** to TCO **27**. Selected signals from BOD are presented in blue and from TCO in grey.

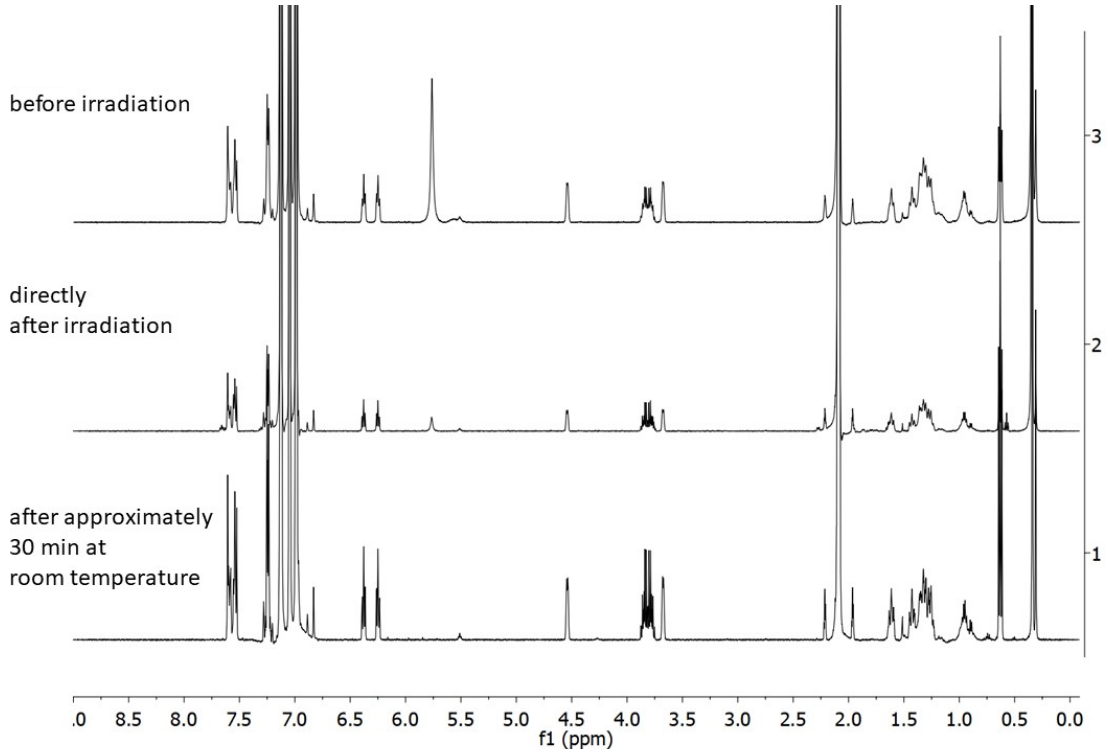

Figure S3.26 ^{1}H-NMR of **24** in toluene-d_8 recorded at -20°C. Spectra from before irradiation (BOD), directly after irradiation (BOD and TCO) and after the sample was kept at room temperature to facilitate the back conversion (BOD).

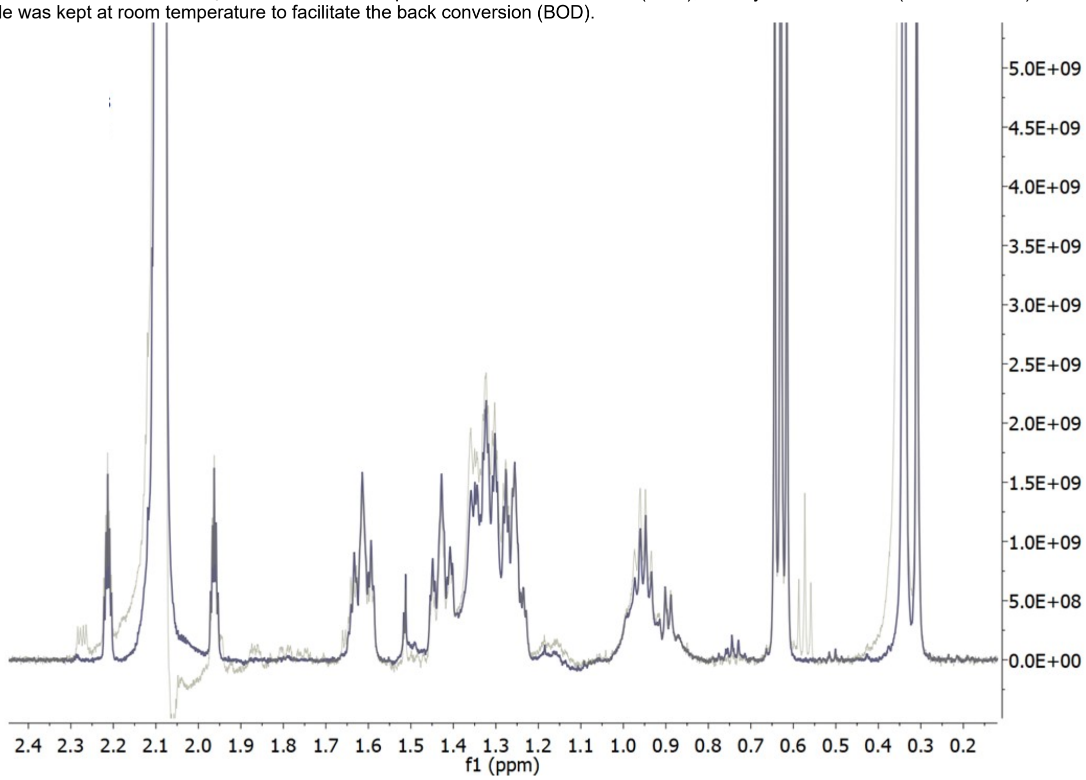

Figure S3.27 Superimposed ^{1}H-NMR spectra of before and after irradiation of BOD **24** to TCO **28**. Selected signals from BOD are presented in blue and from TCO in grey.

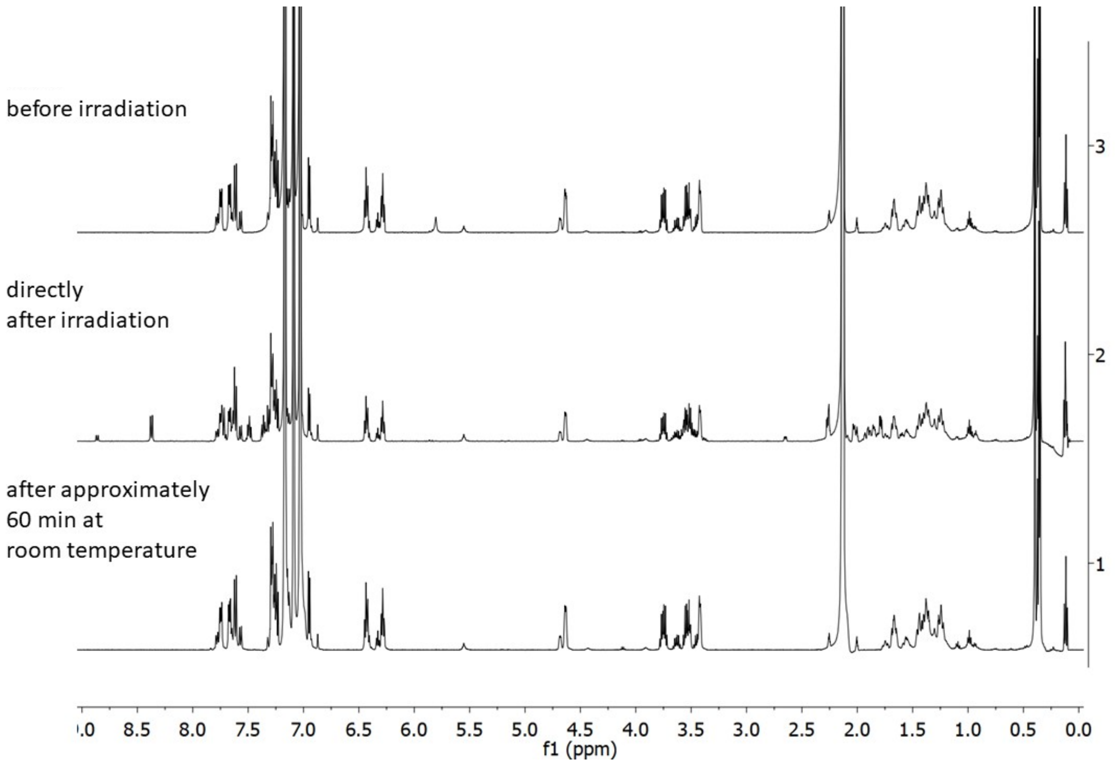

Figure S3.28 ^{1}H-NMR of **25** in toluene-d_8 recorded at -20°C. Spectra from before irradiation (BOD), directly after irradiation (BOD and TCO) and after the sample was kept at room temperature to facilitate the back conversion (BOD).

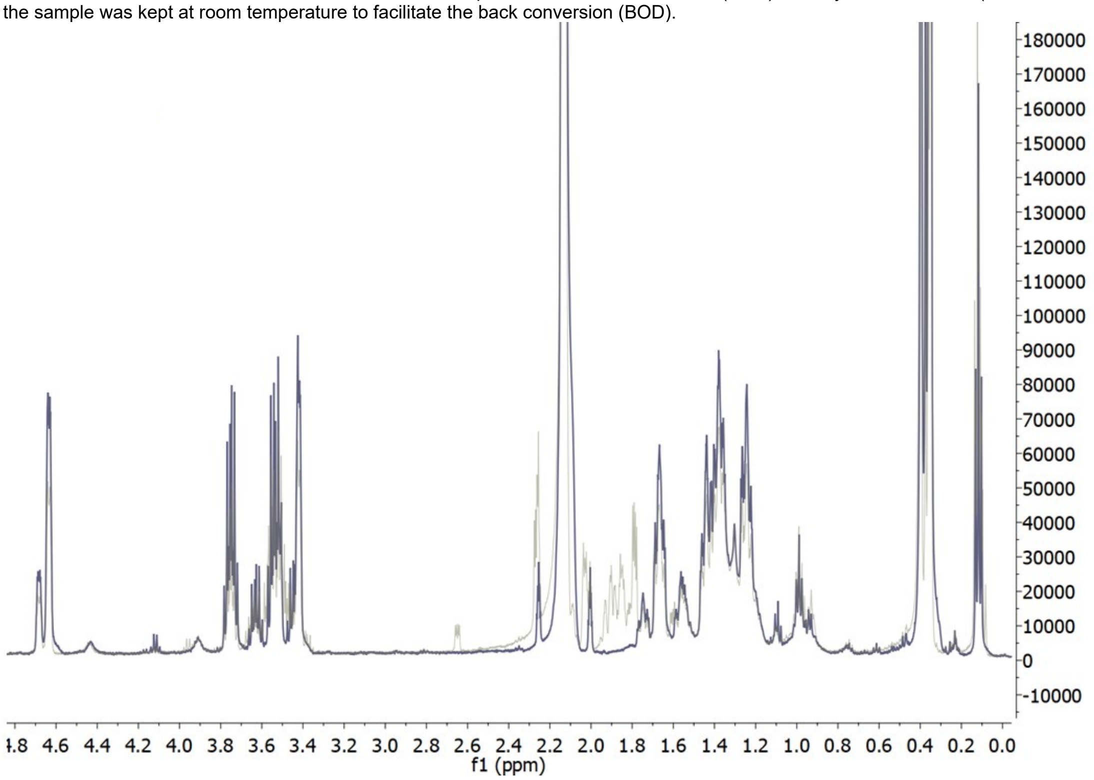

Figure S3.29 Superimposed ^{1}H-NMR spectra of before and after irradiation of BOD **25** to TCO **29**. Selected signals from BOD are presented in blue and from TCO in grey.

4. Kinetic study of back-conversion reaction

Kinetic study oft the back conversion

The bicyclooctadienes were dissolved in toluene and acetonitrile, irradiated with a LED (310 nm) for 2-3 min, to obtain the corresponding photoisomers. Thereafter, the increase of the bicyclooctadiene concentration over time were measured by recording the increase in absorption at 310 or 300 nm with a Cary 50 Bio UV/Vis-spectrophotometer. The measurements were performed at four different temperatures. An exponential fit of the Eyring equation was applied to the obtained data to determine the rate constants at the different temperatures for all compounds. The enthalpy and entropy of activation was derived from the linear form of the Eyring equation, and also the half-life at 25 ^{o}C.

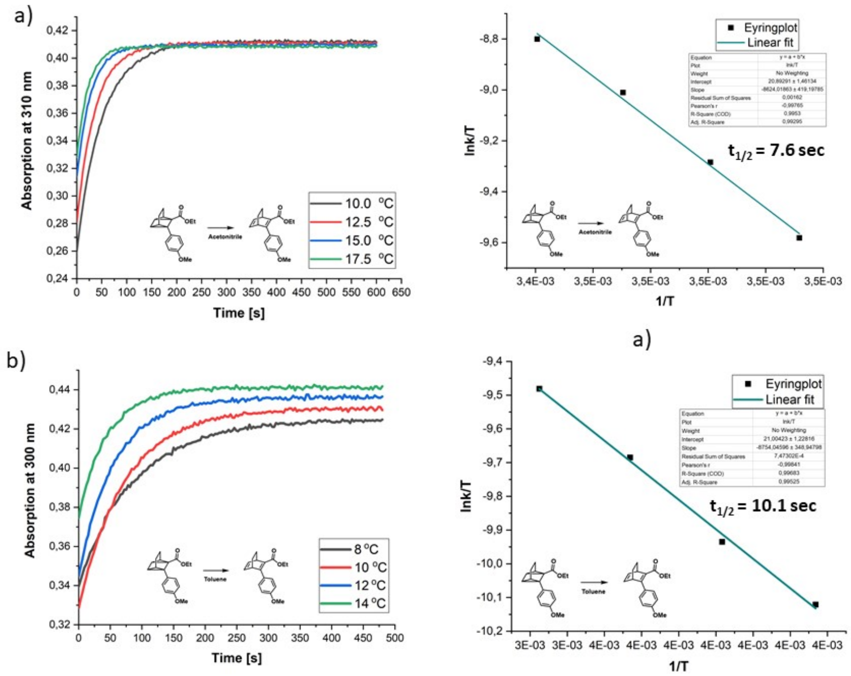

Figure S4.1 Kinetic study of the backconversion for TCO **26** to BOD **22** along with Eyring plot a) in MeCN and b) in toluene.

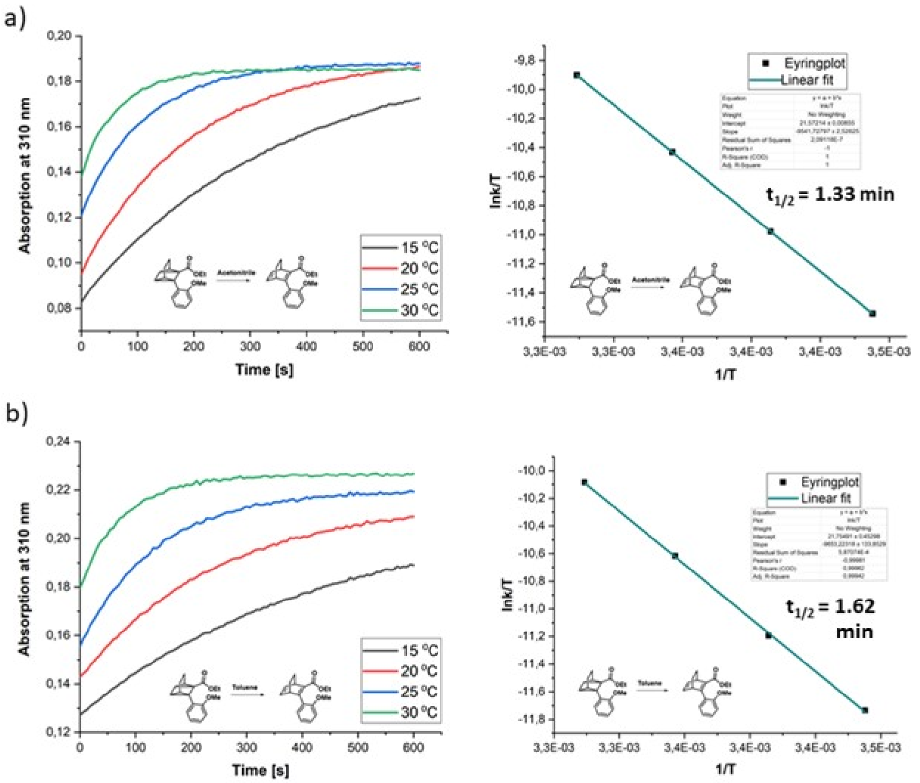

Figure S4.2 Kinetic study of the backconversion for TCO **27** to BOD **23** along with Eyring plot a) in MeCN and b) in toluene.

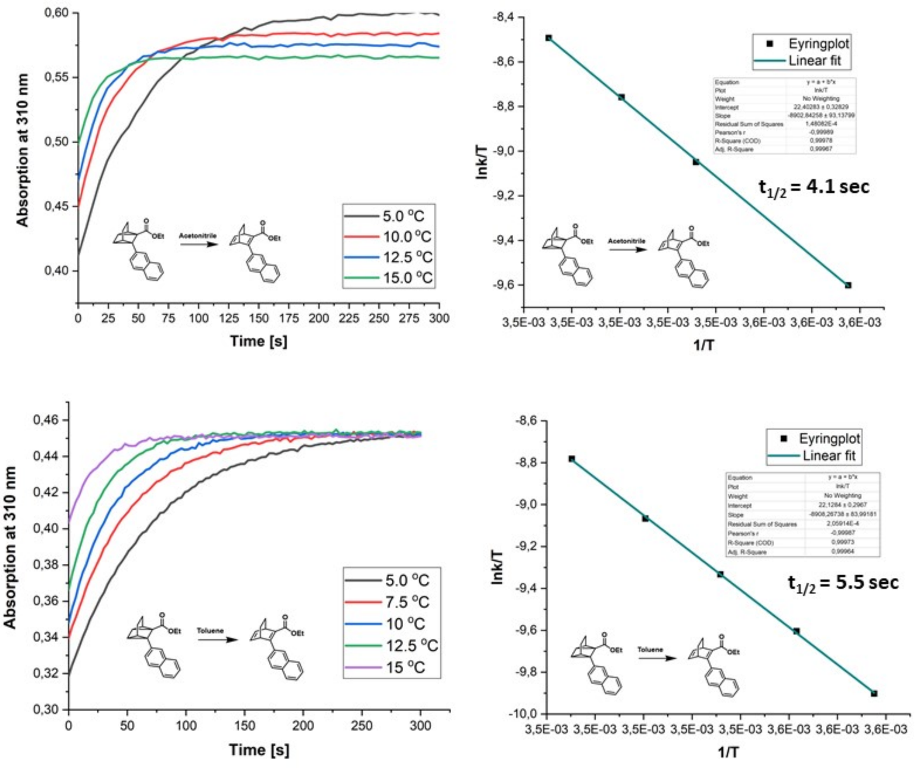

Figure S4.3 Kinetic study of the backconversion for TCO **28** to BOD **24** along with Eyring plot a) in MeCN and b) in toluene.

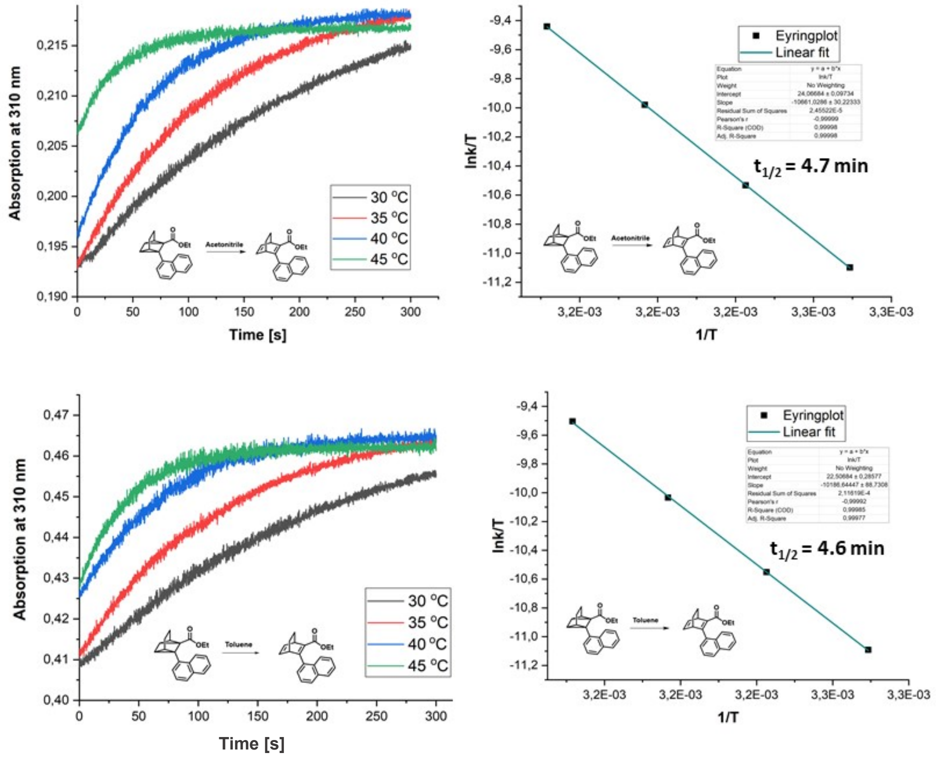

Figure S4.4 Kinetic study of the backconversion for TCO **29** to BOD **25** along with Eyring plot a) in MeCN and b) in toluene.

5. UV/Vis spectroscopy

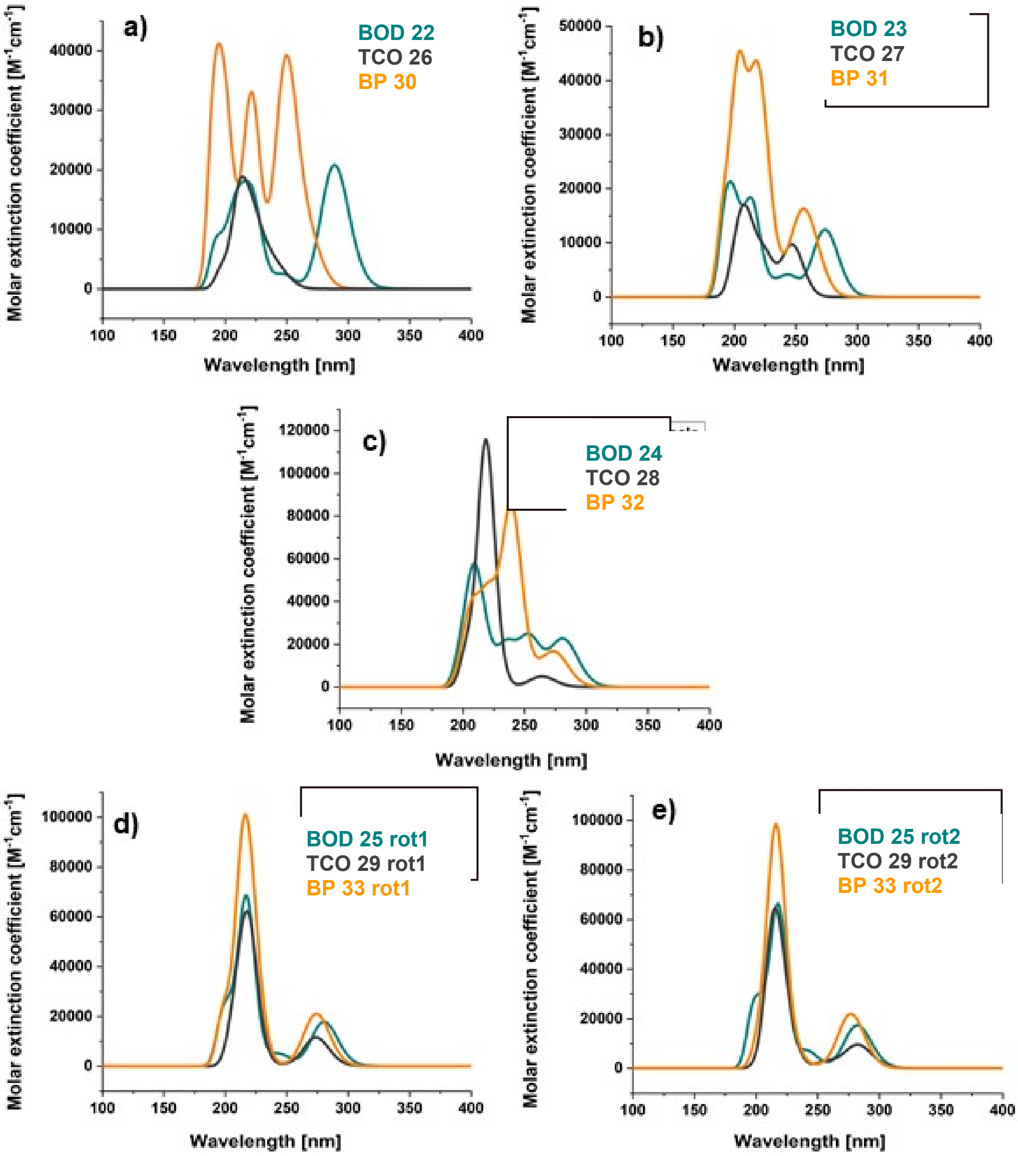

Figure S5.1 Calculated spectra of bicyclooctadienes (BODs), the corresponding photoisomers (TCO) and corresponding aromatic byproducts (BP) formed from a *retro*Diels-Alder process. a) BOD **22**, TCO **26** and BP **30** b) BOD **23**, TCO **27** and BP **31** c) BOD **24**, TCO **28** and BP **32** d) BOD **25** rotamer1, TCO **29** rotamer1 and BP **33** rotamer1 and e) BOD **25** rotamer2, TCO **29** rotamer2 and BP **33** rotamer2.

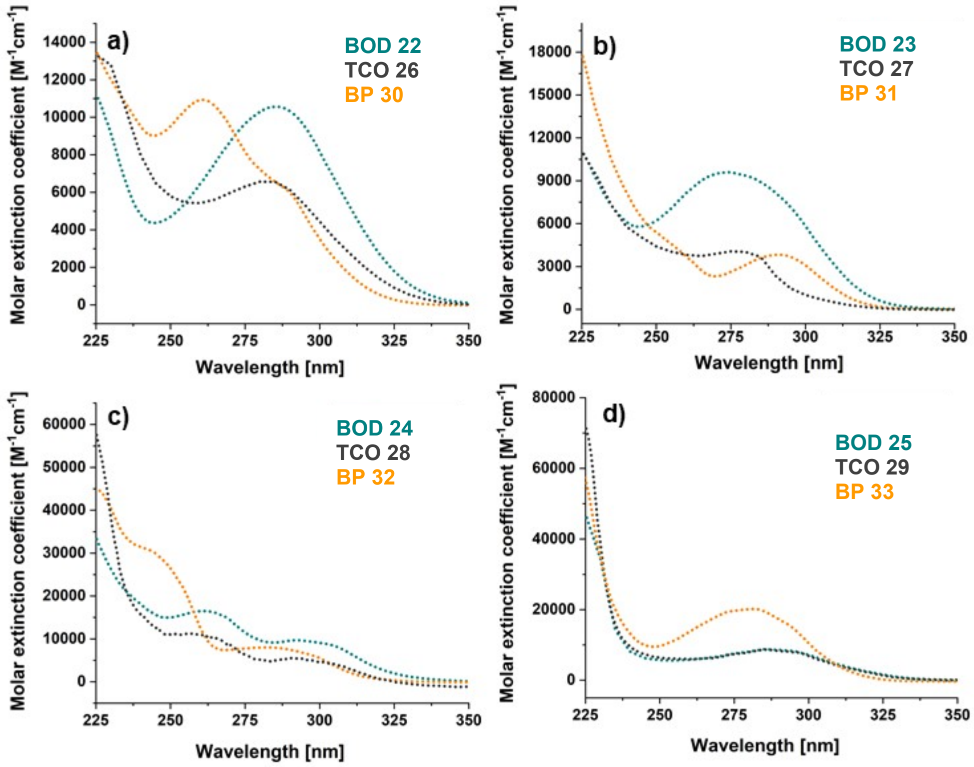

Figure S5.2 Experimental spectra of bicyclooctadienes (BODs), the corresponding photoisomers (TCO) and corresponding aromatic byproducts formed from a retro-Diels-Alder process (BP) a) BOD **22**, TCO **26** and BP **30** b) BOD **23**, TCO **27** and BP **31** c) BOD **24**, TCO **28** and BP **32** and d) BOD **25**, TCO **29** and BP **33.**

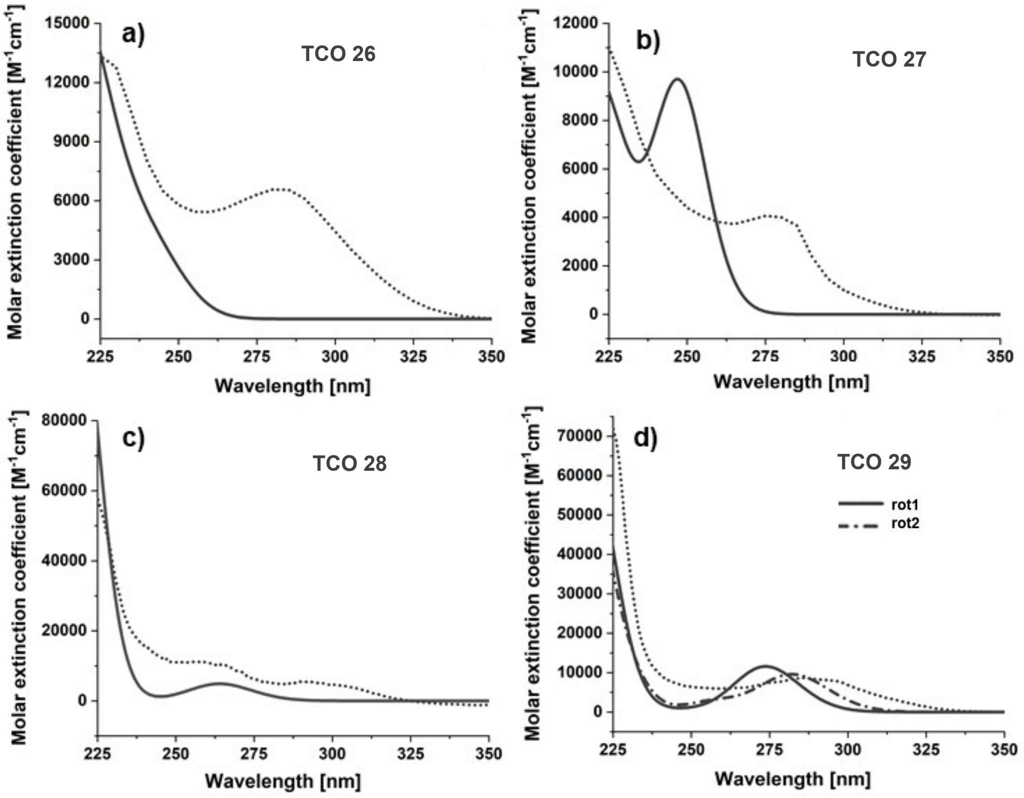

Figure S5.3 Calculated (line) and experimentally obtained (dashed) spectra of tetracyclooctanes (TCO) a) **26**, b) **27** c) **28** and d) **29.**

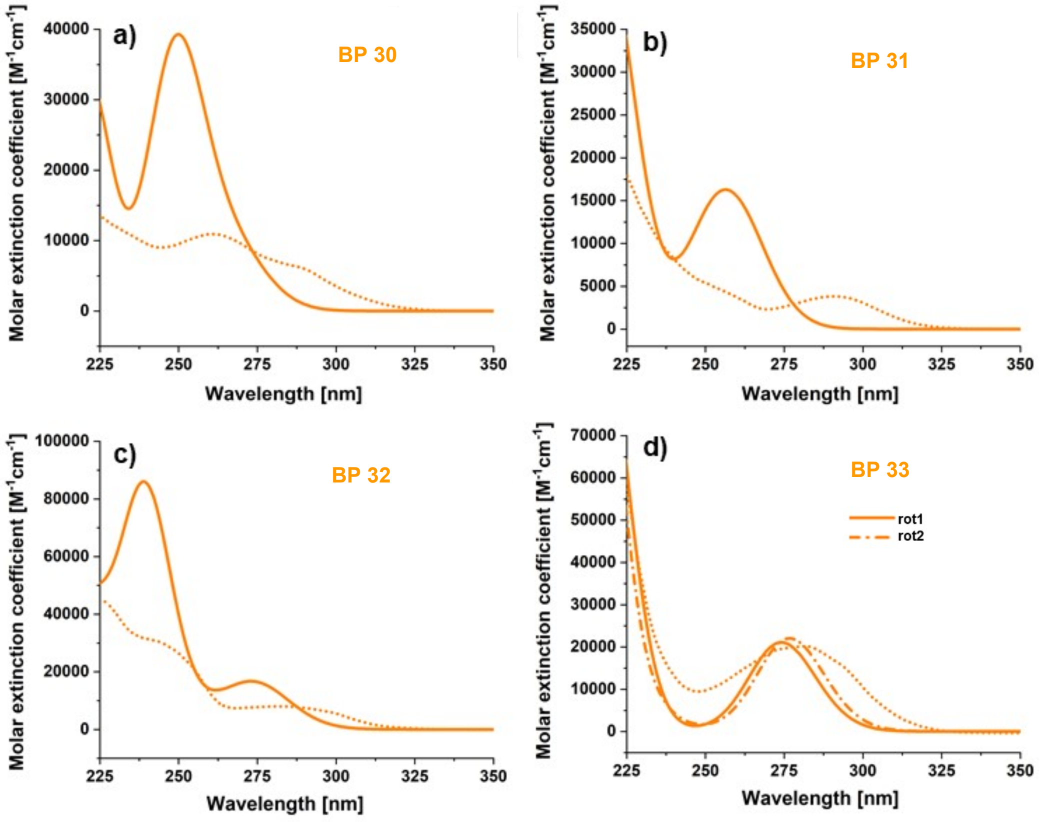

Figure S5.4 Calculated (line) and experimentally obtained (dashed) spectra of aromatic byproducts (BP) a) **30**, b) **31** c) **32** and d) **33**

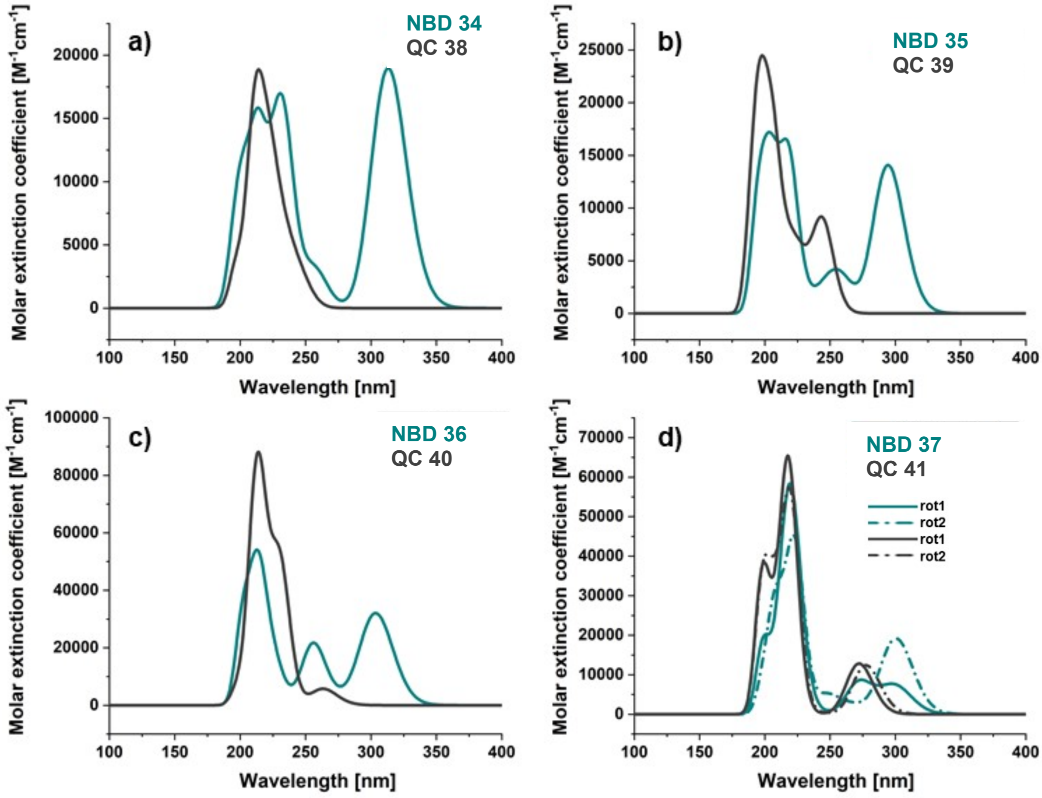

Figure S5.5 Calculated spectra of NBD (green line) and corresponding QC (grey line) a) **34** and **38** b) **35** and **39** c) **36** and **40** and d) **37** and **41**.

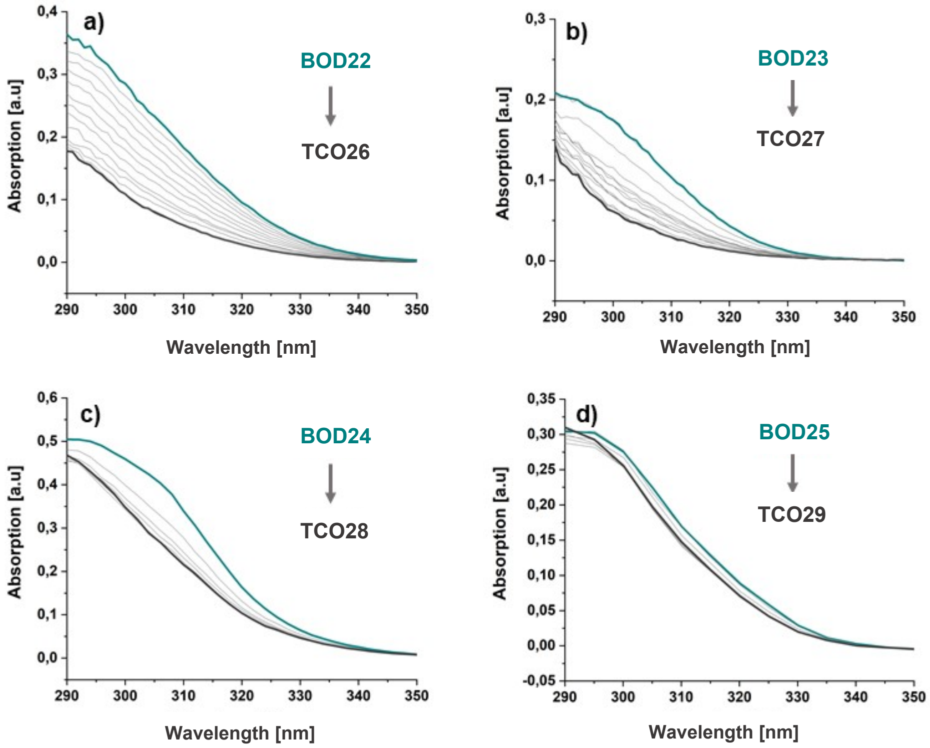

Figure S5.6 Stepwise irradiation BODs in toluene a) **22** b) **23** c) **24** and d) **25**.

.

6. TLC experiments

TLC was performed in 20% EtOAc in hexane on BODs **22** - **25** before and after irradiation along with the corresponding byproducts.

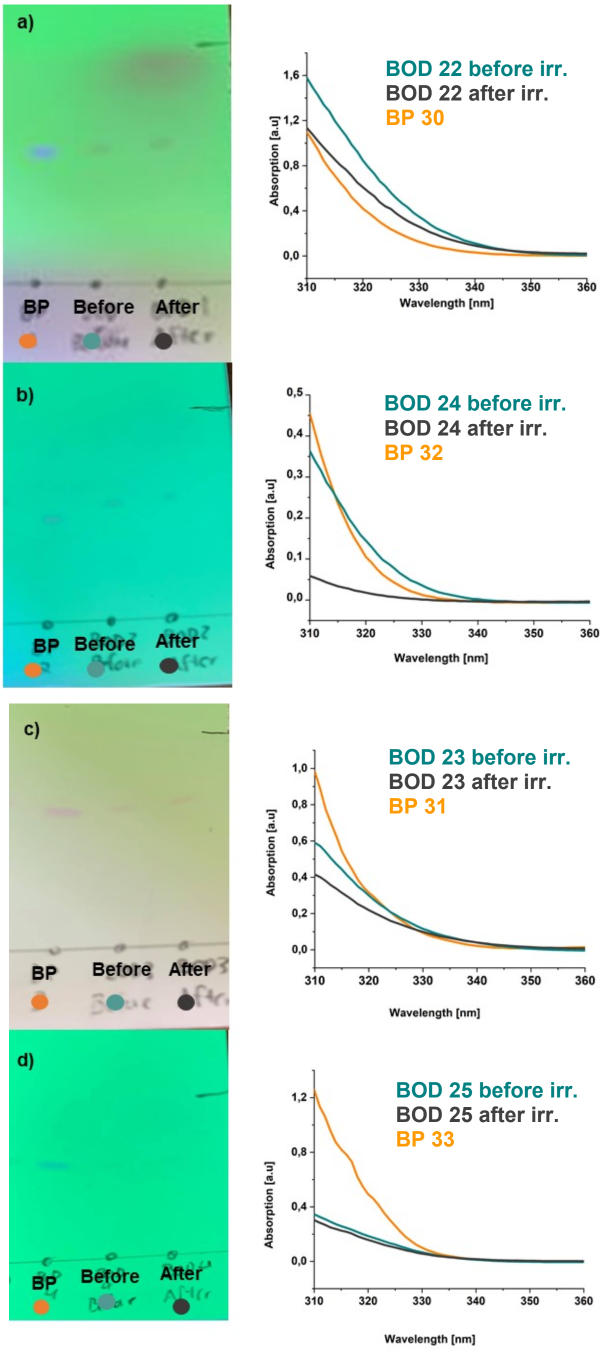

Figure S6.1. TLC of samples of BODs before and after irradiation and corresponding byproducts (BP) along with the UV/Vis spectra. a) **22** and **30**, b) **23** and **31**, c) **24** and **32** and d) **25** and **33**.

7. Quantum yield measurements

Quantum yield measurements

The photonflux of the irradiation source was determined by potassium ferrioxalate actinometry before and after the measurements were performed.[5] A fiber-coupled LED (310 nm) was used as irradiation source for **23** and **25**. The quantum yield measurements were carried out by irradiating a solution of the BODs in toluene at 5 °C and monitoring the decrease in absorption with a Cary 100 -UV-Visible spectrophotometer. The solutions were prepared and stored in the dark to avoid that any photoconversion occurred before the measurements were executed. To ensure that all photons were absorbed, the concentration of the solutions were prepared to be optically thick at the irradiation wavelength (absorption over 2, at 310 nm) When all photons are absorbed, a linear dependence between the decrease in absorption and the irradiation time is obtained and the quantum yield can be determined from the slope.[6]

310 nm LED<br>Photon flux: 1.99 x 10^{-9} mol s^{-1}		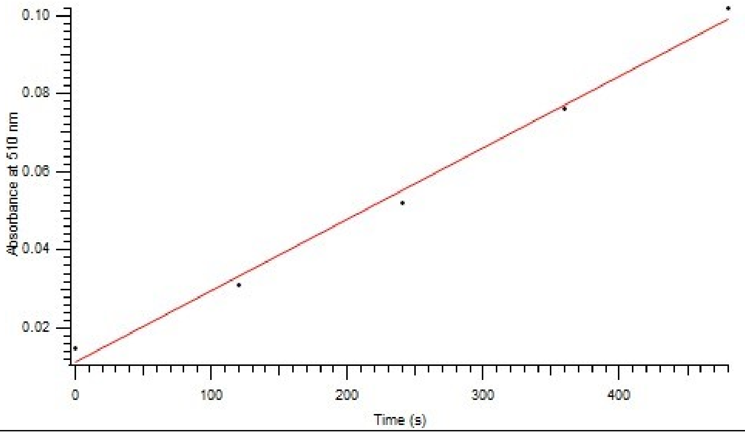
**23**<br>φ_1 = 14.39%<br>φ_2 = 14.47%<br>$\varphi_{average}$ = 14.43 ± 0.04%	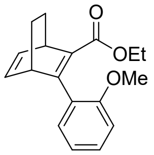<br>Molecular Weight: 284.36	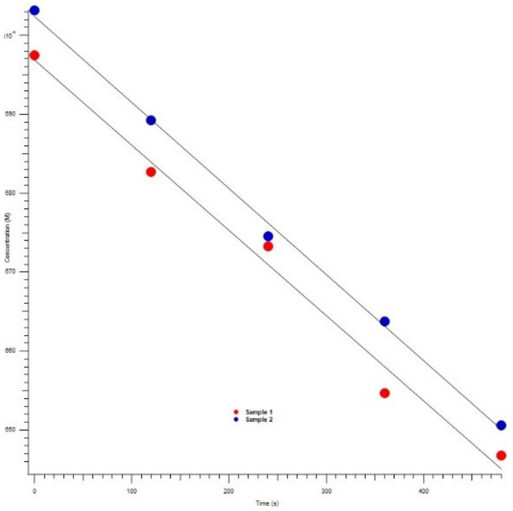

**25**<br><br>φ_1 = 16.46%<br>φ_2 = 22.27%<br><br>$\varphi_{average}$ = 19.37 ± 2.91%	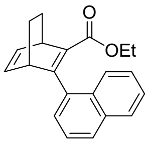<br>Molecular Weight: 304.39	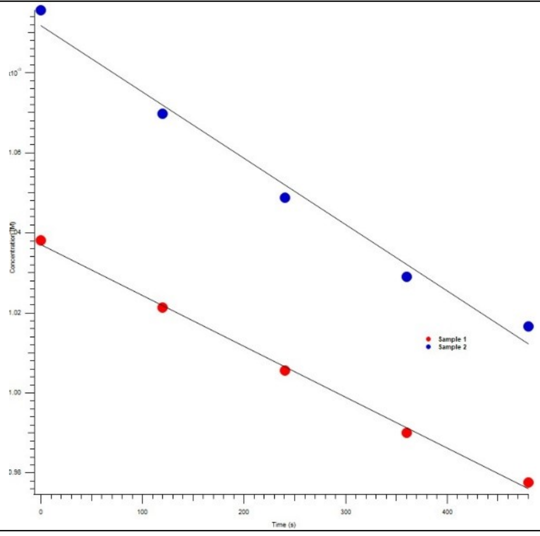

Figure S7.1 Data from quantum yield measurements.

8. Cyclization studies

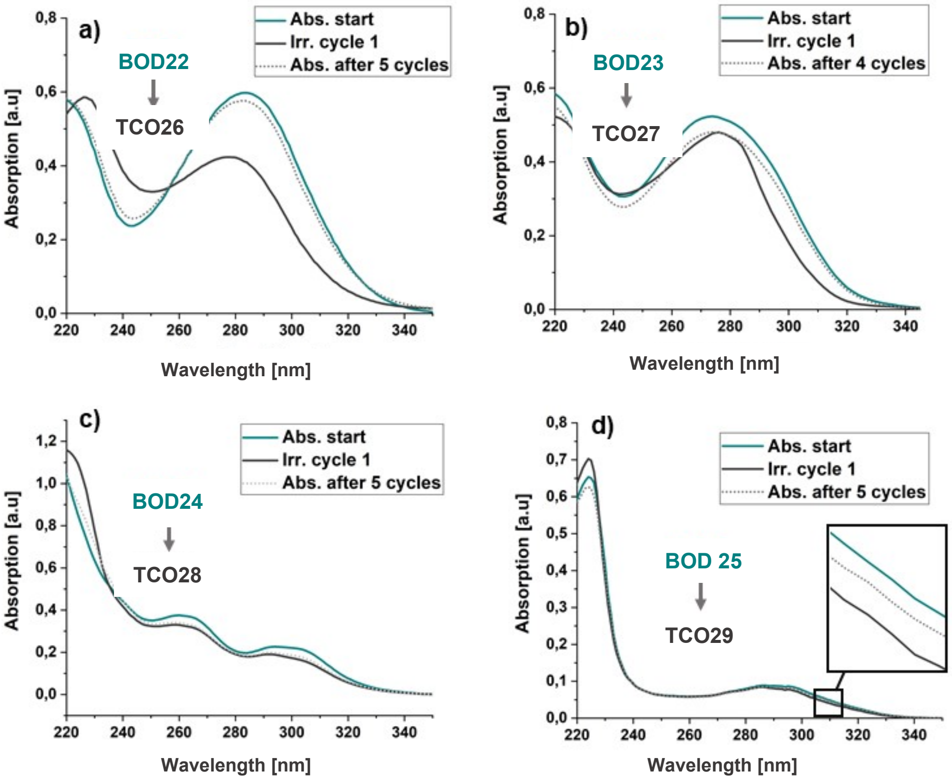

Figure S8.1 Qualitative cyclization study of BODs **22-25**. All compounds were cycled 4 to 5 times. a) **22**/**26** b) **23**/**27** c) **24**/**28** and d) **25**/**29**.

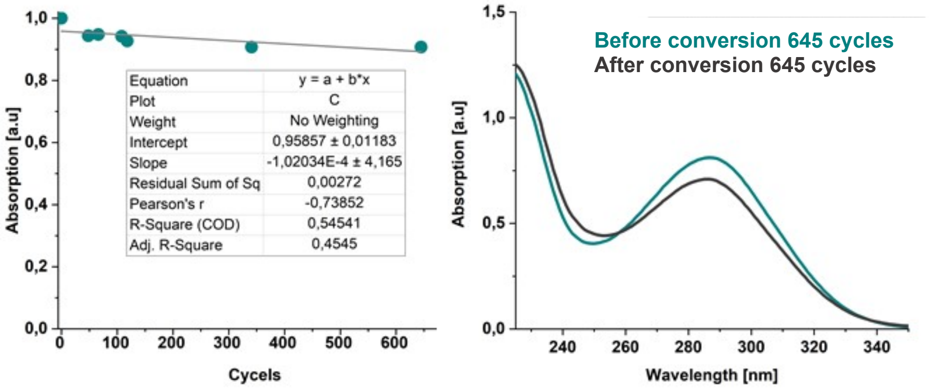

Figure S8.2 Quantitative cycling study of 645 cycles of **22**/**26** a) Absorption after a number of cycles with a linear fir to determine the degradation. b) absorption spectra after 645cycles**.**

9. Computations

Computational details

The structures of the studied BOD/TCO derivatives were obtained by performing geometry optimization in acetonitrile of the five lowest energy conformations of the BOD and TCO derivatives using the M06-2X/6-311++G(d,p)[7],[8] methodology, which has previously shown good performance for these systems.[9] The lowest energy conformation of each system was determined by initially conducting a conformer search in vacuum using a genetic algorithm as implemented in OpenBabel[10] from which the five lowest energy conformations for each of the BOD and TCO systems were determined.
Subsequently, we determined the storage energy of each system as the difference in Gibbs free energy between the TCO and BOD structures at 298.15 K using the lowest energy conformations found previously. For each of the four systems, we determined thermal back reaction barriers and barriers for the retro-Diels-Alder biproduct reaction by optimizing the transition state of each reaction path from the saddle point of a two-dimensional PM6[11] scan of the relevant bonds at the M06-2X/6-311++G(d,p) level of theory in acetonitrile. We further simulated UV-Vis spectra by performing time-dependent density functional theory calculations of the 15 lowest singlet excitation energies and associated oscillator strengths using the M06-2X/6-311++G(d,p) methodology in acetonitrile. The UV-Vis spectra were then simulated by convoluting the excitation energies and oscillator strengths to gaussian functions using a full width half maximum of 3226 cm^{-1}.
For the **25**/**29** system, we identified the two different rotamers and determined separate storage energies, biproduct reaction barrier, and UV-Vis spectra. Meanwhile, the transition state of thermal back reaction could only be converged for the low energy rotamer and we thus only give the barrier for the low energy rotamer.
All density functional theory calculations were performed utilizing the Gaussian 16 program[12] utilizing the polarizable continuum model for solvation[13].

Computed absorption onset, maxima, and extinction coefficients

Table S9.1. Calculated (calc.) absorption onset (λ_{onset}), first absorption maxima (λ_{max}), and molar extinction coefficient at the absorption maxima (ε_{max}) for the NBDs corresponding to BODs **22** – **25**.

NBD	λ_{onset} (calc.) [nm]	λ_{max} (calc.) [nm]	ε_{max} (calc.) [$M^{-1}cm^{-1}$]
**34**	364	313	18971
**35**	337	294	14074
**36**	354	303	32053
**37**	341[a]<br>345[b]	297[a]<br>301[b]	7728[a]<br>19189[b]

[a] Data for rotamer 1, [b] Data for rotamer 2.

Table S9.2 Newman projection and dihedral angles for BODs and the corresponding TCOs and TS.

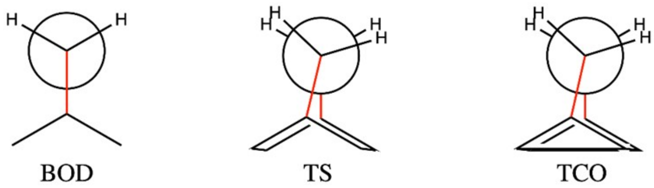

BOD/TCO	BOD	TS	TCO
**22/26**	0.7	20.4	11.8
**23/27**	0.6	13.8	10.6
**24/28**	0.8	20.9	10.9
**25/29**	1.1[a]<br>1.2[b]	19.6[a]<br>15.9[b]	11.7[a]<br>9.3[b]

[a] Data for rotamer 1, [b] Data for rotamer 2.

Light to chemical energy conversion efficiency calculations[14]

$$ɲ = \frac{\Delta E\ \Phi}{h\nu\ N}$$

ɲ = light to chemical energy conversion efficiency
ΔE = energy difference (ΔG) between BOD/TCO or NBD/QC
Φ = quantum yield
h = Plancks constant (6,62607E-34 Js 1,58367E-37 Kcal*s)
ν = frequency (1/s) (calculated from absorption maxima)
N = Avogadro's number (6,02214E23 1/mol)

Table S8.3. Data for **23**, **25**, **35** and **37** used for calculating light to energy conversion efficiency.

BOD/ NBD	λ_{max}[a] [nm]	QY [%]	ΔG [kJ/mol]	ɲ [%]
**23**	274	14.43	146.27	4.8
**25**	284	19.37	147.89	6.8
**35**	294	14.43	54.51	1.9
**37**	297	19.37	51.61	2.4

[a] Experimentally obtained values for BOD and calculated for NBD

Computed geometries and Gibbs Free energies

BOD22

Gibbs free energy at 298.15 K: -923.175076 a.u.

O	-0.50104	2.01694	-0.43499
C	-1.48204	1.31075	-0.37340
O	-2.72485	1.81533	-0.27450
C	-2.84553	3.24861	-0.21363
C	-2.57944	3.76021	1.18877
C	-1.50283	-0.16926	-0.36700
C	-0.43104	-0.95491	-0.12994
C	0.99281	-0.58770	0.00181
C	1.65379	0.17125	-0.96217
C	3.01275	0.45616	-0.85859
C	3.73303	-0.01496	0.23959
O	5.05500	0.21516	0.44227
C	5.74550	0.99628	-0.52137
C	3.08767	-0.78493	1.21254
C	1.74199	-1.07826	1.08234
C	-0.81569	-2.41518	0.07043
H	0.05984	-3.03865	0.23902
C	-1.63303	-2.87120	-1.11909
C	-2.68201	-2.09387	-1.37250
C	-2.82063	-0.92805	-0.41975
H	-3.65346	-0.27810	-0.66756
C	-3.00165	-1.55301	1.00534
C	-1.78150	-2.44540	1.30567
H	-3.87141	3.44657	-0.51987
H	-2.16226	3.69182	-0.93805
H	-3.26040	3.29105	1.90088
H	-2.73816	4.83978	1.21985
H	-1.55167	3.55264	1.48761
H	1.09968	0.54569	-1.81319
H	3.49044	1.04035	-1.63323
H	6.77239	1.06611	-0.17186
H	5.31439	1.99811	-0.59400
H	5.72495	0.51524	-1.50271
H	3.66167	-1.14785	2.05657
H	1.25985	-1.68528	1.84133
H	-1.37769	-3.77252	-1.66256
H	-3.41093	-2.27374	-2.15338
H	-3.92801	-2.12872	1.02581
H	-3.09167	-0.74614	1.73490
H	-2.07934	-3.47933	1.48475
H	-1.24402	-2.09559	2.18903

TCO26

Gibbs free energy at 298.15 K: -923.120600 a.u.

O	2.14960	2.73656	0.48567
C	1.50507	1.77731	0.11438
O	0.27086	1.87190	-0.38399
C	-0.34731	3.17014	-0.38449
C	-1.76639	2.97415	-0.86861
C	1.98223	0.38989	0.14268
C	3.43787	0.09893	-0.16643
C	2.92792	-0.19111	1.19754
C	2.04889	-1.45342	1.27416
C	1.90860	-2.14121	-0.05486
C	3.05504	-2.25598	-1.03472
C	3.81421	-0.93057	-1.20764
C	1.10692	-0.89887	0.21861
C	-0.37749	-0.87028	0.26697
C	-1.14243	-1.28493	-0.81665
C	-2.53667	-1.21107	-0.79844
C	-3.17906	-0.70849	0.33170
O	-4.52791	-0.58596	0.45576
C	-5.32846	-0.97874	-0.64773
C	-2.42296	-0.29782	1.43566
C	-1.04238	-0.37962	1.39718
H	0.22355	3.83367	-1.03703
H	-0.31419	3.57293	0.62961
H	-1.77459	2.58005	-1.88624
H	-2.29585	3.92814	-0.86128
H	-2.29320	2.27308	-0.21766
H	4.01183	1.01936	-0.20492
H	3.27261	0.35039	2.06765
H	1.76353	-1.91484	2.20934
H	1.28927	-3.03056	0.01506
H	2.67465	-2.60192	-1.99812
H	3.72972	-3.03317	-0.66661
H	3.60924	-0.49748	-2.18966
H	4.89208	-1.09927	-1.15897
H	-0.64520	-1.67115	-1.70049
H	-3.09769	-1.54370	-1.66131
H	-6.35880	-0.79507	-0.35357
H	-5.19589	-2.04091	-0.87021
H	-5.09028	-0.38590	-1.53500
H	-2.94084	0.08171	2.30872
H	-0.46196	-0.05499	2.25538

TS for thermal back reaction of BOD22/TCO26
Gibbs free energy at 298.15 K: -923.075507 a.u.

O	2.54850	2.49885	-0.08375
C	1.72999	1.58091	-0.27277
O	0.51063	1.85402	-0.86447
C	0.13684	3.22889	-0.95187
C	-0.37378	3.75157	0.38036
C	1.91478	0.23429	0.02480
C	3.20369	-0.33597	0.58153
C	2.37153	-1.00567	1.59125
C	1.50847	-2.00502	1.11571
C	1.54263	-2.22105	-0.40168
C	2.89282	-2.19021	-1.10562
C	3.91851	-1.32075	-0.35549
C	0.92967	-0.93422	-0.03207
C	-0.53737	-0.75164	0.04877
C	-1.40141	-1.47810	-0.76285
C	-2.78493	-1.30955	-0.68413
C	-3.31175	-0.40624	0.23527
O	-4.63481	-0.16877	0.40226
C	-5.54536	-0.89063	-0.41465
C	-2.44904	0.33281	1.05937
C	-1.08466	0.16924	0.95564
H	-0.64985	3.25803	-1.70678
H	0.98337	3.81947	-1.30394
H	-1.24050	3.17480	0.70912
H	-0.66921	4.79912	0.28645
H	0.40747	3.67937	1.13931
H	3.85175	0.42824	0.99978
H	2.31558	-0.65308	2.61691
H	0.77336	-2.48661	1.74031
H	0.86638	-3.01521	-0.69685
H	2.72351	-1.79567	-2.11006
H	3.26022	-3.21074	-1.21811
H	4.52756	-0.75484	-1.06165
H	4.59419	-1.94687	0.23419
H	-1.00609	-2.17555	-1.49174
H	-3.42494	-1.88227	-1.34085
H	-6.53840	-0.55822	-0.12374
H	-5.45405	-1.96631	-0.24402
H	-5.38009	-0.66990	-1.47225
H	-2.87961	1.03460	1.76384
H	-0.41888	0.75694	1.57846

TS for byproduct reaction of BOD22/TCO26
Gibbs free energy at 298.15 K: -923.126663 a.u.

O	-0.38603	1.94810	-0.83618
C	-1.38553	1.31982	-0.57691
O	-2.53012	1.90948	-0.19173
C	-2.52248	3.34563	-0.08156
C	-1.92294	3.78614	1.23951
C	-1.53259	-0.15757	-0.62082
C	-0.50253	-1.02710	-0.33971
C	0.92567	-0.68286	-0.11738
C	1.67747	-0.02295	-1.08707
C	3.03456	0.23524	-0.90862
C	3.66330	-0.16933	0.26889
O	4.97623	0.04024	0.54705
C	5.75511	0.72342	-0.42298
C	2.92775	-0.84431	1.24698
C	1.58288	-1.10540	1.04685
C	-0.89768	-2.39525	-0.13131
H	-0.11962	-3.08663	0.17444
C	-1.98119	-2.90967	-0.90845
C	-2.98629	-2.04709	-1.20514
C	-2.85783	-0.71980	-0.68841
H	-3.67796	-0.02989	-0.83889
C	-3.05879	-1.17599	1.34369
C	-2.01173	-2.03740	1.67993
H	-3.57134	3.62803	-0.15546
H	-1.97734	3.76183	-0.92874
H	-2.47120	3.34322	2.07271
H	-1.98473	4.87263	1.32472
H	-0.87494	3.49253	1.30573
H	1.19720	0.29656	-2.00272
H	3.58150	0.74557	-1.68970
H	6.75814	0.79226	-0.00963
H	5.36349	1.72812	-0.60253
H	5.78486	0.16751	-1.36374
H	3.42971	-1.15879	2.15411
H	1.03554	-1.64218	1.81350
H	-2.03401	-3.96870	-1.13015
H	-3.90803	-2.36287	-1.67811
H	-4.06028	-1.57749	1.24980
H	-3.01157	-0.13935	1.65626
H	-2.21252	-3.09068	1.83108
H	-1.17051	-1.64742	2.23923

Byproduct 30
Gibbs free energy at 298.15 K: -844.670281 a.u.

O	2.94914	-1.79961	-0.09027
C	3.18283	-0.61731	-0.18415
O	4.41358	-0.11457	-0.28897
C	5.51153	-1.05085	-0.26173
C	5.84821	-1.45206	1.16047
C	2.12832	0.43958	-0.20430
C	2.44686	1.79350	-0.31193
C	1.42249	2.73303	-0.31682
C	0.09638	2.32665	-0.21787
C	-0.23598	0.97075	-0.10679
C	-1.65080	0.53897	0.00117
C	-2.57996	1.28857	0.73691
C	-3.89976	0.88777	0.84143
C	-4.33221	-0.28134	0.20677
O	-5.64274	-0.59525	0.36463
C	-6.11643	-1.78305	-0.25213
C	-3.42464	-1.04071	-0.53109
C	-2.09853	-0.62346	-0.62472
C	0.79936	0.03451	-0.09949
H	6.33373	-0.51317	-0.72990
H	5.25031	-1.91413	-0.87350
H	6.08534	-0.57129	1.75917
H	6.71895	-2.11019	1.15506
H	5.01562	-1.98409	1.62139
H	3.48085	2.10206	-0.39071
H	1.65671	3.78674	-0.40746
H	-0.69312	3.06934	-0.24635
H	-2.25949	2.18662	1.25323
H	-4.61475	1.46137	1.41896
H	-7.17155	-1.85240	-0.00041
H	-5.58966	-2.66003	0.13292
H	-6.00199	-1.73254	-1.33799
H	-3.73138	-1.94415	-1.04017
H	-1.40942	-1.21358	-1.21870
H	0.58406	-1.02235	0.00700

BOD23
Gibbs free energy at 298.15 K: -923.17674 a.u.

O	-0.42463	2.87502	0.00282
C	-0.26960	1.78084	-0.49429
O	0.75127	1.47427	-1.30012
C	1.76327	2.48011	-1.49412
C	2.75266	2.47961	-0.34675
C	-1.21938	0.66321	-0.31786
C	-0.90606	-0.63885	-0.24647
C	0.43546	-1.25817	-0.27041
C	0.67752	-2.34759	-1.10505
C	1.91740	-2.98008	-1.13789
C	2.92719	-2.53142	-0.29750
C	2.70644	-1.46094	0.56756
C	1.46877	-0.81867	0.58189
O	1.18400	0.22892	1.39756
C	2.10105	0.53258	2.43810
C	-2.11699	-1.51343	0.04603
H	-1.86029	-2.57095	0.05021
C	-3.23745	-1.17541	-0.91056
C	-3.53184	0.12170	-0.95885
C	-2.67807	0.97225	-0.04461
H	-2.88547	2.03664	-0.12684
C	-2.94586	0.45132	1.40996
C	-2.59811	-1.04995	1.46799
H	1.28076	3.45056	-1.61058
H	2.24269	2.20357	-2.43161
H	2.25908	2.74737	0.58834
H	3.54463	3.20430	-0.54515
H	3.20454	1.49082	-0.24141
H	-0.12411	-2.69557	-1.74851
H	2.08606	-3.81531	-1.80596
H	3.89690	-3.01508	-0.30130
H	3.50230	-1.13664	1.22442
H	1.64396	1.33450	3.01336
H	3.05873	0.87548	2.03933
H	2.26100	-0.33836	3.07917
H	-3.75644	-1.94963	-1.46259
H	-4.32594	0.55395	-1.55553
H	-3.99318	0.62438	1.66159
H	-2.33148	1.02596	2.10536
H	-3.46449	-1.64837	1.75281
H	-1.80273	-1.24598	2.18977

TCO 27
Gibbs free energy at 298.15 K: -923.121035 a.u.

O	-0.74103	-1.29101	-1.24208
C	-1.31314	-0.66903	-0.37408
O	-2.39462	-1.13329	0.27413
C	-2.92294	-2.40014	-0.16173
C	-3.77666	-2.23878	-1.40417
C	-0.93533	0.67272	0.08148
C	-1.95585	1.79029	0.16957
C	-1.25125	1.35166	1.40660
C	0.20357	1.84707	1.48419
C	0.60510	2.67786	0.29995
C	-0.33030	3.67077	-0.35375
C	-1.74511	3.10186	-0.55289
C	0.53095	1.18498	0.14898
C	1.69733	0.32687	-0.15610
C	2.70312	0.73219	-1.02663
C	3.78477	-0.09778	-1.32286
C	3.85270	-1.35815	-0.74599
C	2.85889	-1.79093	0.13347
C	1.79402	-0.94695	0.43561
O	0.79878	-1.26573	1.30518
C	0.65208	-2.62870	1.67179
H	-3.51304	-2.75232	0.68282
H	-2.09404	-3.08816	-0.33037
H	-4.58911	-1.53403	-1.21891
H	-4.21135	-3.20189	-1.67823
H	-3.17668	-1.87934	-2.24082
H	-2.96670	1.39915	0.11252
H	-1.76886	0.88687	2.23356
H	0.80033	1.78081	2.38268
H	1.63272	3.01993	0.38022
H	0.08918	3.98534	-1.31170
H	-0.36590	4.56027	0.28032
H	-1.94239	2.93147	-1.61405
H	-2.49530	3.81617	-0.20664
H	2.62587	1.71098	-1.48811
H	4.55649	0.23620	-2.00546
H	4.68147	-2.01864	-0.97255
H	2.92980	-2.77428	0.57915
H	-0.27199	-2.68906	2.24301
H	1.48519	-2.96582	2.29360
H	0.57764	-3.25841	0.78047

TS for thermal back reaction of BOD23/TCO27
Gibbs free energy at 298.15 K: -923.064199 a.u.

O	1.46211	-1.61093	-0.71457
C	1.89892	-0.51701	-0.34122
O	3.24849	-0.23723	-0.50229
C	4.07318	-1.30779	-0.95981
C	4.44437	-2.24776	0.17418
C	1.21096	0.54411	0.29330
C	1.69990	1.97972	0.00648
C	0.76185	2.03002	-1.12716
C	-0.60540	2.00309	-0.83082
C	-0.87791	1.95806	0.65907
C	-0.05819	2.83006	1.59920
C	1.38608	2.99705	1.10669
C	-0.31471	0.65919	0.22802
C	-1.19915	-0.53328	0.27433
C	-0.73314	-1.62235	1.01736
C	-1.53838	-2.72361	1.28356
C	-2.82809	-2.76365	0.77333
C	-3.32256	-1.69650	0.02908
C	-2.52707	-0.57495	-0.20439
O	-2.97473	0.50750	-0.88807
C	-4.30416	0.49408	-1.38921
H	4.96149	-0.82332	-1.36723
H	3.56710	-1.84643	-1.76168
H	4.95435	-1.70229	0.97082
H	5.11344	-3.03066	-0.19004
H	3.55005	-2.71889	0.58446
H	2.73725	1.99971	-0.30520
H	1.11265	1.92210	-2.15039
H	-1.37366	1.92896	-1.57831
H	-1.93909	1.91719	0.87472
H	-0.07864	2.35194	2.58110
H	-0.55352	3.79676	1.70191
H	2.09524	2.84760	1.92192
H	1.54662	4.00328	0.71220
H	0.27817	-1.59175	1.39697
H	-1.15135	-3.54575	1.87225
H	-3.46325	-3.62344	0.95102
H	-4.33187	-1.74040	-0.35518
H	-4.43984	1.44794	-1.89277
H	-5.02739	0.40237	-0.57547
H	-4.44359	-0.32061	-2.10352

TS for byproduct reaction of BOD23/TCO27
Gibbs free energy at 298.15 K: -923.130713 a.u.

O	-0.50910	2.82237	-0.12297
C	-0.30026	1.74574	-0.63750
O	0.79492	1.47075	-1.35068
C	1.81236	2.48873	-1.40879
C	2.65390	2.48878	-0.14860
C	-1.25652	0.61532	-0.58913
C	-0.90242	-0.70671	-0.52790
C	0.48100	-1.23530	-0.42128
C	0.91569	-2.25579	-1.26119
C	2.18602	-2.81430	-1.13006
C	3.02607	-2.35734	-0.12465
C	2.60983	-1.34905	0.74465
C	1.34507	-0.78366	0.59566
O	0.86724	0.20623	1.39494
C	1.59844	0.53174	2.56695
C	-1.98308	-1.62397	-0.29201
H	-1.72168	-2.66915	-0.16473
C	-3.27893	-1.30281	-0.81034
C	-3.61741	0.01077	-0.83307
C	-2.63578	0.93394	-0.34545
H	-2.89081	1.98662	-0.30248
C	-2.64785	0.36930	1.66288
C	-2.29760	-0.98511	1.71597
H	1.33623	3.45529	-1.57483
H	2.40545	2.22646	-2.28312
H	2.04727	2.74989	0.71926
H	3.45840	3.22026	-0.24613
H	3.09765	1.50310	0.00816
H	0.24384	-2.61022	-2.03572
H	2.50788	-3.59981	-1.80229
H	4.01435	-2.78452	-0.00179
H	3.27468	-1.01499	1.53001
H	1.00695	1.28122	3.08823
H	2.57803	0.94966	2.32215
H	1.72291	-0.34938	3.20219
H	-3.98531	-2.09200	-1.03798
H	-4.61554	0.35311	-1.07790
H	-3.67817	0.65274	1.84125
H	-1.91030	1.10801	1.95568
H	-3.06551	-1.72512	1.90567
H	-1.30195	-1.25765	2.04720

Byproduct 31
Gibbs free energy at 298.15 K: -844.667922 a.u.

O	4.15736	0.95681	-0.30463
C	3.15835	0.28192	-0.21512
O	3.17276	-1.02360	0.05863
C	4.46111	-1.63557	0.27837
C	4.95188	-1.37869	1.68909
C	1.77746	0.81426	-0.40695
C	1.62982	2.16921	-0.69733
C	0.35667	2.69604	-0.87830
C	-0.76194	1.87775	-0.76710
C	-0.62644	0.51491	-0.47786
C	-1.80567	-0.38645	-0.40585
C	-1.78974	-1.60499	-1.08220
C	-2.87257	-2.47940	-1.03969
C	-3.99564	-2.12900	-0.30369
C	-4.03966	-0.92058	0.38904
C	-2.95161	-0.04884	0.34242
O	-2.91141	1.13126	1.01156
C	-4.03827	1.49499	1.79430
C	0.65531	-0.00703	-0.29760
H	5.15979	-1.25662	-0.46733
H	4.28734	-2.69552	0.10333
H	5.11748	-0.31389	1.85494
H	5.89557	-1.90427	1.84586
H	4.22744	-1.74603	2.41757
H	2.51086	2.79366	-0.77831
H	0.23390	3.74708	-1.10988
H	-1.75127	2.29333	-0.91233
H	-0.91273	-1.86085	-1.66687
H	-2.83726	-3.41649	-1.58090
H	-4.85059	-2.79328	-0.25980
H	-4.92070	-0.67177	0.96437
H	-3.79569	2.45779	2.23687
H	-4.21802	0.76315	2.58596
H	-4.93217	1.59270	1.17310
H	0.78199	-1.05612	-0.06023

BOD24
Gibbs free energy at 298.15 K: -962.268681 a.u.

O	-2.75090	2.48438	0.42955
C	-1.95417	1.70743	-0.05046
O	-0.79877	2.08942	-0.59765
C	-0.41059	3.46335	-0.41688
C	0.97008	3.61018	-1.01439
C	-2.16171	0.24564	-0.10766
C	-1.20585	-0.69595	-0.01981
C	0.23621	-0.49261	0.23198
C	0.67414	0.39345	1.25833
C	2.00531	0.58731	1.49784
C	2.98915	-0.08572	0.72311
C	4.37887	0.10519	0.93710
C	5.30101	-0.56283	0.17319
C	4.87693	-1.45739	-0.83982
C	3.54095	-1.66363	-1.06654
C	2.56298	-0.98412	-0.29245
C	1.17309	-1.17821	-0.50456
C	-1.77495	-2.10530	-0.05126
H	-0.99044	-2.85899	-0.03095
C	-2.71411	-2.25122	-1.22749
C	-3.65362	-1.31069	-1.28752
C	-3.57467	-0.30068	-0.16274
H	-4.30988	0.49638	-0.24603
C	-3.76322	-1.11418	1.16365
C	-2.66850	-2.19769	1.23763
H	-0.41748	3.68529	0.65286
H	-1.14405	4.10635	-0.90655
H	1.66833	2.92880	-0.52420
H	1.32399	4.63319	-0.87837
H	0.95275	3.38827	-2.08247
H	-0.06810	0.90665	1.86059
H	2.32890	1.25834	2.28646
H	4.69839	0.79022	1.71522
H	6.36035	-0.40972	0.34174
H	5.61630	-1.97929	-1.43562
H	3.21131	-2.34820	-1.84079
H	0.85737	-1.86646	-1.28311
H	-2.62316	-3.08170	-1.91692
H	-4.43832	-1.26630	-2.03285
H	-4.75938	-1.55854	1.16717
H	-3.69745	-0.42729	2.00906
H	-3.10115	-3.19767	1.28557
H	-2.03497	-2.06379	2.11655

TCO28
Gibbs free energy at 298.15 K: -962.210446 a.u.

O	1.99168	2.78451	0.56791
C	1.59641	1.78238	0.01124
O	0.60364	1.77114	-0.88487
C	-0.16326	2.97762	-1.05483
C	-1.27229	3.03428	-0.02229
C	2.15919	0.44101	0.21044
C	3.66143	0.24309	0.23838
C	2.87763	-0.04397	1.46777
C	2.08166	-1.36225	1.40051
C	2.27810	-2.09483	0.10093
C	3.61385	-2.15340	-0.60646
C	4.31985	-0.78765	-0.65065
C	1.36718	-0.89998	0.14741
C	-0.09221	-0.93764	-0.12639
C	-0.56019	-1.16821	-1.44911
C	-1.89695	-1.13396	-1.73677
C	-2.85163	-0.86148	-0.71830
C	-4.24394	-0.79704	-0.98676
C	-5.13722	-0.52019	0.01613
C	-4.68022	-0.29835	1.33823
C	-3.34133	-0.36022	1.62750
C	-2.39298	-0.64255	0.60902
C	-0.99825	-0.69110	0.87363
H	-0.56311	2.90963	-2.06531
H	0.50144	3.83827	-0.98812
H	-1.90921	2.15053	-0.10314
H	-1.88370	3.92433	-0.18232
H	-0.85653	3.07678	0.98642
H	4.17633	1.19735	0.29149
H	2.98976	0.54026	2.37060
H	1.62850	-1.81983	2.26895
H	1.71365	-3.02232	0.07224
H	3.46931	-2.53647	-1.61875
H	4.23530	-2.88302	-0.08110
H	4.32347	-0.39194	-1.66907
H	5.36506	-0.88979	-0.35108
H	0.16534	-1.36443	-2.23150
H	-2.24822	-1.30903	-2.74831
H	-4.58885	-0.96745	-2.00121
H	-6.19810	-0.46969	-0.19867
H	-5.39619	-0.07923	2.12157
H	-2.98656	-0.19086	2.63873
H	-0.64923	-0.51162	1.88661

TS for thermal back reaction BOD24/TCO28
Gibbs free energy at 298.15 K: -962.164540 a.u.

O	2.30907	2.77103	0.10806
C	1.72447	1.71950	-0.20692
O	0.63958	1.75871	-1.06338
C	0.05511	3.03943	-1.29999
C	-0.86620	3.43902	-0.16027
C	2.05702	0.43077	0.19705
C	3.32004	0.07209	0.94972
C	2.49230	-0.82198	1.77317
C	1.92093	-1.91413	1.10413
C	2.24114	-2.00890	-0.39342
C	3.65430	-1.66841	-0.85012
C	4.35718	-0.68931	0.10791
C	1.33736	-0.89054	-0.07421
C	-0.13963	-0.98370	-0.20640
C	-0.73276	-1.91598	-1.09926
C	-2.09524	-2.00869	-1.20932
C	-2.94597	-1.18620	-0.42774
C	-4.36160	-1.26250	-0.51411
C	-5.15125	-0.44785	0.25373
C	-4.56548	0.48476	1.14771
C	-3.20370	0.58207	1.25144
C	-2.35724	-0.24928	0.46646
C	-0.94874	-0.16176	0.54527
H	-0.50349	2.92807	-2.23006
H	0.84079	3.78075	-1.44807
H	-1.66992	2.70826	-0.04766
H	-1.31106	4.41731	-0.35630
H	-0.30651	3.49478	0.77579
H	3.73237	0.90741	1.50834
H	2.21657	-0.57857	2.79502
H	1.20772	-2.57510	1.57033
H	1.79892	-2.89661	-0.83012
H	3.57262	-1.23032	-1.84733
H	4.22761	-2.59061	-0.94916
H	4.95072	0.03309	-0.45402
H	5.03706	-1.22595	0.77595
H	-0.10526	-2.54276	-1.72063
H	-2.54015	-2.71273	-1.90409
H	-4.80554	-1.97731	-1.19864
H	-6.23048	-0.51233	0.18208
H	-5.20387	1.12076	1.74933
H	-2.74935	1.29284	1.93382
H	-0.50021	0.57069	1.20898

TS for byproduct reaction BOD24/TCO28
Gibbs free energy at 298.15 K: -962.219822 a.u.

O	-2.76073	2.31398	0.14945
C	-1.97048	1.56157	-0.37690
O	-0.89026	1.97631	-1.04300
C	-0.63335	3.39635	-1.05579
C	0.03755	3.84502	0.22736
C	-2.13526	0.08723	-0.39488
C	-1.12148	-0.82962	-0.28272
C	0.29866	-0.50955	0.00080
C	0.63015	0.41036	1.03553
C	1.93407	0.67396	1.35086
C	2.99400	0.04142	0.64724
C	4.35772	0.29973	0.94466
C	5.35548	-0.32946	0.24620
C	5.03726	-1.24956	-0.78302
C	3.72926	-1.51986	-1.09026
C	2.67401	-0.88330	-0.38373
C	1.31072	-1.14599	-0.67763
C	-1.53929	-2.20201	-0.19510
H	-0.76485	-2.94231	-0.02537
C	-2.72624	-2.59953	-0.88884
C	-3.72621	-1.68492	-0.96054
C	-3.48098	-0.41609	-0.34222
H	-4.27615	0.32076	-0.34004
C	-3.42994	-1.05690	1.64739
C	-2.39500	-1.99215	1.75362
H	-1.57425	3.92060	-1.22263
H	0.01650	3.54286	-1.91651
H	-0.60931	3.66438	1.08724
H	0.24585	4.91521	0.17139
H	0.97915	3.31404	0.37264
H	-0.17050	0.89406	1.58684
H	2.17565	1.37110	2.14639
H	4.59583	1.00455	1.73417
H	6.39401	-0.12568	0.47856
H	5.83581	-1.73994	-1.32699
H	3.48096	-2.22413	-1.87707
H	1.07384	-1.85339	-1.46652
H	-2.85066	-3.62647	-1.21037
H	-4.71127	-1.92428	-1.34204
H	-4.45553	-1.40508	1.66517
H	-3.28146	-0.06015	2.04657
H	-2.63679	-3.04508	1.82886
H	-1.46277	-1.70349	2.22603

Byproduct 32

Gibbs free energy at 298.15 K: -883.760929 a.u.

O	4.97970	-0.19910	-0.14377
C	3.81354	0.09927	-0.03386
O	3.39453	1.34409	0.18910
C	4.41519	2.35701	0.29968
C	3.71624	3.67454	0.53926
C	2.69137	-0.88032	-0.13464
C	3.00665	-2.21703	-0.37645
C	1.98641	-3.15491	-0.46597
C	0.66073	-2.76119	-0.31531
C	0.33135	-1.42245	-0.07516
C	-1.08513	-1.00492	0.07490
C	-2.00587	-1.84362	0.76505
C	-3.31605	-1.47871	0.91189
C	-3.79543	-0.25384	0.37640
C	-5.14939	0.15113	0.50987
C	-5.57728	1.33963	-0.02223
C	-4.67082	2.18097	-0.71384
C	-3.35794	1.81571	-0.85730
C	-2.88480	0.59085	-0.31436
C	-1.53207	0.18621	-0.44912
C	1.36352	-0.48580	0.01665
H	4.99700	2.36326	-0.62348
H	5.07903	2.08951	1.12347
H	3.05109	3.91504	-0.29123
H	4.45885	4.46868	0.62902
H	3.13323	3.64016	1.46059
H	4.04418	-2.50550	-0.48986
H	2.22078	-4.19447	-0.65964
H	-0.12885	-3.49880	-0.40542
H	-1.65365	-2.77303	1.19787
H	-4.00582	-2.12061	1.44928
H	-5.83891	-0.49771	1.03929
H	-6.61254	1.64158	0.08288
H	-5.02285	3.11746	-1.12996
H	-2.66078	2.45698	-1.38588
H	-0.85264	0.83030	-0.99843
H	1.13648	0.55207	0.22612

BOD25 rotamer 1

Gibbs free energy at 298.15 K: -962.266482 a.u.

O	-2.99733	1.90642	0.11203
C	-1.91974	1.37966	-0.06244
O	-0.79492	2.06310	-0.28461
C	-0.87968	3.50153	-0.24842
C	-1.36491	4.05186	-1.57500
C	-1.72789	-0.08737	-0.06742
C	-0.60313	-0.74952	0.24356
C	0.67705	-0.19515	0.73536
C	0.73355	0.44302	1.95186
C	1.96144	0.91665	2.47027
C	3.12226	0.74249	1.76524
C	3.10722	0.09031	0.50359
C	1.87428	-0.38600	-0.02157
C	1.86988	-1.01335	-1.29684
C	3.03463	-1.17566	-2.00188
C	4.26344	-0.71332	-1.47256
C	4.29620	-0.09222	-0.25166
C	-0.78635	-2.26087	0.22326
H	0.14758	-2.78084	0.43005
C	-1.42549	-2.67725	-1.08278
C	-2.53942	-2.00914	-1.37260
C	-2.92353	-0.98263	-0.32862
H	-3.80524	-0.40550	-0.59601
C	-3.14676	-1.78147	1.00122
C	-1.84904	-2.54106	1.34427
H	0.13701	3.82522	-0.03099
H	-1.53405	3.79595	0.57212
H	-0.70768	3.72963	-2.38424
H	-1.36065	5.14292	-1.54088
H	-2.38063	3.71547	-1.78455
H	-0.17885	0.57886	2.52254
H	1.97559	1.41321	3.43319
H	4.06831	1.09909	2.15804
H	0.92967	-1.35575	-1.71649
H	3.01724	-1.65523	-2.97341
H	5.17624	-0.84718	-2.04077
H	5.23242	0.27336	0.15666
H	-1.00682	-3.47647	-1.68260
H	-3.15911	-2.18845	-2.24271
H	-3.98279	-2.46877	0.86526
H	-3.41378	-1.07894	1.79259
H	-2.01917	-3.61705	1.39741
H	-1.44131	-2.21888	2.30450

BOD25 rotamer 2
Gibbs free energy at 298.15 K: -962.267262 a.u.

O	1.12571	-1.96317	-0.44481
C	1.85594	-1.03733	-0.17666
O	3.17428	-1.17911	0.02569
C	3.72183	-2.50420	-0.12343
C	3.95414	-2.83617	-1.58438
C	1.43630	0.37603	-0.03529
C	0.19128	0.75432	0.29104
C	-0.94556	-0.10748	0.68199
C	-0.88607	-0.84682	1.83951
C	-1.99354	-1.61184	2.27376
C	-3.15250	-1.62522	1.54482
C	-3.25467	-0.87760	0.34147
C	-2.14226	-0.10891	-0.09967
C	-2.24985	0.61521	-1.31784
C	-3.41031	0.58981	-2.04777
C	-4.52141	-0.16529	-1.60160
C	-4.44236	-0.88338	-0.43736
C	0.04312	2.26379	0.41100
H	-0.98395	2.54965	0.63186
C	0.59407	2.92599	-0.83228
C	1.82920	2.54025	-1.14274
C	2.41444	1.52849	-0.18039
H	3.40472	1.18959	-0.47072
C	2.44144	2.23159	1.22024
C	1.00560	2.65918	1.58647
H	3.04805	-3.21903	0.34892
H	4.65806	-2.47288	0.43094
H	3.01116	-2.85921	-2.13113
H	4.42486	-3.81780	-1.66425
H	4.61512	-2.09854	-2.04234
H	0.02400	-0.83613	2.42948
H	-1.91815	-2.18249	3.19172
H	-4.00782	-2.20553	1.87369
H	-1.39693	1.18366	-1.67399
H	-3.47899	1.14576	-2.97531
H	-5.43202	-0.17696	-2.18866
H	-5.28636	-1.47157	-0.09309
H	0.02040	3.66870	-1.37361
H	2.40693	2.92692	-1.97342
H	3.10941	3.09251	1.17071
H	2.84555	1.53406	1.95597
H	0.93807	3.73678	1.74057
H	0.66512	2.16836	2.50037

TCO29 rotamer 1
Gibbs free energy at 298.15 K: -962.210153 a.u.

O	-1.81991	2.96837	-0.07794
C	-1.20281	1.92556	-0.15130
O	0.12138	1.86292	-0.27836
C	0.86861	3.08913	-0.25374
C	2.32826	2.69210	-0.23835
C	-1.80967	0.58873	-0.10588
C	-3.17474	0.40590	0.51983
C	-3.03447	0.19068	-0.94367
C	-2.34332	-1.13143	-1.32523
C	-1.99186	-1.94787	-0.11828
C	-2.87827	-2.04506	1.10281
C	-3.41476	-0.67580	1.54845
C	-1.13161	-0.76152	-0.49269
C	0.30636	-0.92004	-0.82841
C	0.70857	-1.16682	-2.11705
C	2.07960	-1.32411	-2.44137
C	3.03623	-1.21756	-1.46940
C	2.66360	-0.96112	-0.12111
C	1.28885	-0.82416	0.20796
C	0.93116	-0.57022	1.55800
C	1.89261	-0.44680	2.52855
C	3.26312	-0.57522	2.19824
C	3.63712	-0.83028	0.90441
H	0.58770	3.65456	0.63683
H	0.60868	3.68007	-1.13444
H	2.54349	2.07711	0.63884
H	2.95741	3.58313	-0.21076
H	2.57619	2.11363	-1.13081
H	-3.61958	1.36727	0.75441
H	-3.49881	0.84637	-1.66701
H	-2.36033	-1.53411	-2.32849
H	-1.49816	-2.87644	-0.38891
H	-2.32030	-2.51634	1.91480
H	-3.70596	-2.71679	0.86041
H	-2.93575	-0.36031	2.47883
H	-4.48584	-0.73343	1.75239
H	-0.03490	-1.23051	-2.90370
H	2.36353	-1.51955	-3.46886
H	4.08846	-1.32487	-1.71056
H	-0.11796	-0.46760	1.81546
H	1.60588	-0.24977	3.55494
H	4.01376	-0.47400	2.97327
H	4.68517	-0.93342	0.64317

TCO29 rotamer 2
Gibbs free energy at 298.15 K: -962.212996 a.u.

O	0.95161	2.93985	-0.00385
C	0.84217	1.79839	-0.39814
O	0.00319	1.42483	-1.36898
C	-0.88843	2.42565	-1.89989
C	-2.10119	2.59476	-1.00668
C	1.64428	0.66904	0.09564
C	3.14296	0.85193	0.24963
C	2.32600	0.56615	1.45776
C	1.87944	-0.90606	1.55641
C	2.36904	-1.74076	0.40739
C	3.74304	-1.57339	-0.20166
C	4.12111	-0.09687	-0.40376
C	1.19987	-0.82110	0.19419
C	-0.15410	-1.29654	-0.19006
C	-0.29257	-2.24698	-1.17260
C	-1.56752	-2.69415	-1.59534
C	-2.70011	-2.17548	-1.02977
C	-2.60696	-1.19839	-0.00155
C	-1.32588	-0.75842	0.43442
C	-1.26153	0.20259	1.47970
C	-2.40429	0.70412	2.04903
C	-3.67616	0.27566	1.60380
C	-3.77195	-0.65601	0.60309
H	-1.16693	2.04435	-2.88090
H	-0.34006	3.35992	-2.02035
H	-2.61176	1.63862	-0.87114
H	-2.79531	3.30207	-1.46521
H	-1.81099	2.97590	-0.02667
H	3.40782	1.90196	0.17364
H	2.21501	1.28281	2.26043
H	1.46264	-1.33716	2.45590
H	2.04613	-2.77363	0.49829
H	3.78131	-2.10809	-1.15310
H	4.46202	-2.06125	0.46107
H	4.15909	0.14709	-1.46798
H	5.11725	0.10121	-0.00223
H	0.59768	-2.65325	-1.64073
H	-1.63841	-3.44361	-2.37479
H	-3.68270	-2.50329	-1.35187
H	-0.29672	0.54172	1.83730
H	-2.33419	1.43674	2.84463
H	-4.57130	0.68294	2.05876
H	-4.74232	-0.99729	0.25799

TS for thermal back reaction BOD25/TCO29 rotamer 1
Gibbs free energy at 298.15 K: -962.163000 a.u.

O	-1.94912	2.78773	-0.65275
C	-1.27847	1.74406	-0.55716
O	0.05261	1.74628	-0.92907
C	0.72907	3.00012	-0.86861
C	1.09225	3.34821	0.56462
C	-1.73088	0.51572	-0.07984
C	-3.20994	0.16683	0.00220
C	-2.98558	-0.87728	-1.01641
C	-2.16189	-1.94751	-0.64651
C	-1.61877	-1.85358	0.77777
C	-2.55576	-1.38501	1.88207
C	-3.63956	-0.42984	1.35172
C	-1.03302	-0.83866	-0.12171
C	0.34726	-1.01505	-0.65693
C	0.59516	-1.37574	-1.95475
C	1.92256	-1.49212	-2.43267
C	2.98042	-1.23950	-1.60278
C	2.76103	-0.85940	-0.25127
C	1.43088	-0.74617	0.23513
C	1.22531	-0.34276	1.58144
C	2.29019	-0.08295	2.40488
C	3.61552	-0.20637	1.92397
C	3.84314	-0.58308	0.62636
H	0.11111	3.77712	-1.32026
H	1.62594	2.86763	-1.47544
H	0.18844	3.44650	1.17069
H	1.63686	4.29434	0.60151
H	1.71805	2.56359	0.99615
H	-3.84842	0.97272	-0.34642
H	-3.30349	-0.74978	-2.04815
H	-1.85305	-2.71464	-1.33775
H	-0.96830	-2.68977	1.01131
H	-1.94522	-0.88247	2.63645
H	-3.00424	-2.25551	2.36183
H	-3.80679	0.38749	2.05403
H	-4.58913	-0.95651	1.22563
H	-0.23080	-1.56171	-2.63204
H	2.09133	-1.78143	-3.46292
H	3.99999	-1.32552	-1.96260
H	0.21372	-0.21845	1.95184
H	2.11889	0.22781	3.42875
H	4.44803	0.00244	2.58528
H	4.85544	-0.67530	0.24746

TS for thermal back reaction BOD25/TCO29 rotamer 2
Gibbs free energy at 298.15 K: -962.163391 a.u.

O	1.35798	2.96982	-0.37266
C	1.03180	1.78276	-0.55370
O	-0.04044	1.47340	-1.37130
C	-0.90797	2.54373	-1.74113
C	-1.94613	2.81012	-0.66516
C	1.66778	0.65674	-0.04291
C	2.93325	0.68820	0.79502
C	2.24697	-0.13586	1.79966
C	1.83259	-1.40664	1.36953
C	2.28430	-1.78307	-0.05568
C	3.66660	-1.34673	-0.51784
C	4.12616	-0.03695	0.15003
C	1.19683	-0.79809	-0.02985
C	-0.20031	-1.20848	-0.33333
C	-0.39497	-2.06500	-1.39193
C	-1.69274	-2.44822	-1.80378
C	-2.78759	-1.97988	-1.13333
C	-2.63391	-1.10994	-0.01941
C	-1.33222	-0.70781	0.39089
C	-1.22085	0.15687	1.51015
C	-2.33434	0.58878	2.18519
C	-3.62588	0.19226	1.77202
C	-3.76872	-0.63679	0.69034
H	-1.38559	2.21466	-2.66531
H	-0.31978	3.43775	-1.94976
H	-2.54202	1.91361	-0.47765
H	-2.61295	3.61738	-0.97800
H	-1.46046	3.10100	0.26854
H	3.17328	1.68448	1.15513
H	1.94398	0.26196	2.76473
H	1.22992	-2.06773	1.97189
H	1.99946	-2.80336	-0.28856
H	3.61507	-1.22323	-1.60202
H	4.37570	-2.15116	-0.32007
H	4.57595	0.63202	-0.58475
H	4.87960	-0.23954	0.91618
H	0.46120	-2.43246	-1.94550
H	-1.80765	-3.11165	-2.65228
H	-3.78894	-2.26649	-1.43616
H	-0.24658	0.50423	1.82095
H	-2.22370	1.25140	3.03559
H	-4.49744	0.54609	2.30969
H	-4.75328	-0.95077	0.36039

TS for byproduct reaction BOD25/TCO29 rotamer 1
Gibbs free energy at 298.15 K: -962.219191 a.u.

O	-2.79791	1.95390	0.26682
C	-1.80380	1.39348	-0.13955
O	-0.68603	2.03229	-0.48808
C	-0.64476	3.45683	-0.26870
C	-1.29269	4.20378	-1.41754
C	-1.71261	-0.07600	-0.32424
C	-0.60391	-0.82654	-0.03624
C	0.63681	-0.29658	0.58643
C	0.56864	0.31763	1.81541
C	1.73179	0.79109	2.46473
C	2.95810	0.64112	1.87457
C	3.07437	0.01748	0.60432
C	1.90504	-0.45823	-0.05352
C	2.04041	-1.04895	-1.33983
C	3.27225	-1.17761	-1.92748
C	4.43603	-0.72028	-1.26382
C	4.33650	-0.13423	-0.02982
C	-0.78040	-2.25002	-0.11866
H	0.06029	-2.86897	0.17767
C	-1.70039	-2.76283	-1.08747
C	-2.80479	-2.01381	-1.33913
C	-2.92810	-0.78549	-0.61405
H	-3.82286	-0.18861	-0.74957
C	-3.19699	-1.59611	1.30403
C	-2.06689	-2.36922	1.58679
H	0.41691	3.68634	-0.19192
H	-1.13056	3.67992	0.68143
H	-0.80394	3.95104	-2.35980
H	-1.19406	5.27872	-1.25537
H	-2.35288	3.95951	-1.48873
H	-0.39712	0.43795	2.29679
H	1.64450	1.26733	3.43400
H	3.85730	0.99654	2.36616
H	1.15343	-1.39290	-1.85920
H	3.35848	-1.62837	-2.90909
H	5.40332	-0.83010	-1.73954
H	5.22138	0.22797	0.48263
H	-1.57215	-3.76289	-1.48361
H	-3.62481	-2.37154	-1.94966
H	-4.12871	-2.09454	1.06583
H	-3.30725	-0.62852	1.78018
H	-2.13689	-3.44880	1.53732
H	-1.32058	-1.99276	2.27717

TS for byproduct reaction BOD25/TCO29 rotamer 2
Gibbs free energy at 298.15 K: -962.219554 a.u.

O	-2.56137	2.26420	-0.45206
C	-1.66665	1.49075	-0.71273
O	-0.50364	1.85735	-1.25500
C	-0.26242	3.27148	-1.40385
C	0.23387	3.86807	-0.10197
C	-1.76502	0.02486	-0.50430
C	-0.75898	-0.74731	0.00994
C	0.52437	-0.21026	0.53163
C	0.50442	0.69964	1.56278
C	1.70249	1.19590	2.12720
C	2.91372	0.77475	1.64901
C	2.98115	-0.15864	0.58022
C	1.77750	-0.66154	0.01110
C	1.86638	-1.57240	-1.07650
C	3.08577	-1.97469	-1.55731
C	4.28198	-1.48615	-0.97963
C	4.22848	-0.59742	0.06161
C	-1.10702	-2.11228	0.29207
H	-0.35031	-2.72616	0.76985
C	-2.07682	-2.74869	-0.54644
C	-3.08014	-1.96782	-1.02357
C	-3.05713	-0.58680	-0.64528
H	-3.87185	0.05384	-0.96369
C	-3.43144	-0.83393	1.40742
C	-2.40768	-1.63721	1.91923
H	-1.17822	3.75180	-1.74740
H	0.49232	3.33626	-2.18543
H	-0.51928	3.76763	0.68170
H	0.44658	4.92937	-0.24377
H	1.14802	3.36751	0.22151
H	-0.44984	1.04046	1.95332
H	1.65195	1.91042	2.94033
H	3.83876	1.14701	2.07603
H	0.95457	-1.94360	-1.53009
H	3.13735	-2.66838	-2.38815
H	5.23871	-1.81396	-1.36852
H	5.13985	-0.21114	0.50543
H	-2.07008	-3.82528	-0.66648
H	-3.93402	-2.37432	-1.55160
H	-4.41728	-1.26160	1.27151
H	-3.42160	0.23002	1.61469
H	-2.61239	-2.67651	2.14473
H	-1.62428	-1.18754	2.51875

Byproduct 33 rotamer 1
Gibbs free energy at 298.15 K: -883.758293 a.u.

O	-4.57088	-1.20853	0.14383
C	-3.58426	-0.51074	0.11326
O	-3.59685	0.78768	0.41143
C	-4.87126	1.34962	0.78562
C	-4.64831	2.81500	1.07701
C	-2.22653	-1.00570	-0.26043
C	-2.08765	-2.35025	-0.59955
C	-0.83724	-2.84450	-0.95018
C	0.26854	-2.00113	-0.95703
C	0.14143	-0.65013	-0.61597
C	1.31283	0.26627	-0.66584
C	1.25291	1.38372	-1.46679
C	2.34964	2.26801	-1.58209
C	3.50517	2.02218	-0.89168
C	3.60606	0.89337	-0.03662
C	2.49977	0.00517	0.09345
C	2.61229	-1.08274	1.00317
C	3.76632	-1.29184	1.71319
C	4.87408	-0.42555	1.55772
C	4.79055	0.64393	0.70667
C	-1.11752	-0.15939	-0.27020
H	-5.24355	0.81011	1.65805
H	-5.57084	1.19713	-0.03786
H	-3.93785	2.94218	1.89492
H	-5.59411	3.27601	1.36543
H	-4.26717	3.33050	0.19444
H	-2.95893	-2.99320	-0.58698
H	-0.72311	-3.88626	-1.22364
H	1.24113	-2.38794	-1.24151
H	0.35276	1.57520	-2.04056
H	2.27074	3.13245	-2.23036
H	4.35762	2.68682	-0.98098
H	1.76883	-1.74644	1.14607
H	3.83012	-2.12346	2.40492
H	5.78120	-0.60576	2.12212
H	5.62714	1.32463	0.59071
H	-1.23312	0.88290	0.00083

Byproduct 33 rotamer 2
Gibbs free energy at 298.15 K: -883.757687 a.u.

O	-2.66295	-1.49107	-1.15129
C	-2.95927	-0.53979	-0.46681
O	-4.21149	-0.25410	-0.11336
C	-5.23547	-1.14998	-0.59155
C	-6.55907	-0.63579	-0.07623
C	-1.96990	0.43874	0.07369
C	-2.36120	1.51949	0.86536
C	-1.39845	2.40439	1.33430
C	-0.05677	2.21198	1.01837
C	0.34584	1.12984	0.22974
C	1.77700	0.95966	-0.13964
C	2.43124	1.99697	-0.76426
C	3.77928	1.87967	-1.17277
C	4.46427	0.71579	-0.95252
C	3.83770	-0.37399	-0.29280
C	2.48299	-0.25747	0.13293
C	1.90442	-1.34577	0.84347
C	2.61939	-2.49039	1.08507
C	3.95427	-2.61430	0.63302
C	4.54817	-1.57661	-0.03446
C	-0.62665	0.24665	-0.24015
H	-5.01476	-2.15366	-0.22450
H	-5.19824	-1.16600	-1.68204
H	-6.56860	-0.61936	1.01445
H	-7.36147	-1.29043	-0.41946
H	-6.75229	0.37184	-0.44676
H	-3.40631	1.66148	1.10714
H	-1.69152	3.24532	1.95088
H	0.69169	2.90181	1.39249
H	1.89149	2.91493	-0.96961
H	4.25891	2.71323	-1.67160
H	5.49535	0.60936	-1.27179
H	0.88891	-1.26294	1.20967
H	2.16163	-3.30606	1.63210
H	4.50542	-3.52677	0.82674
H	5.57615	-1.65297	-0.37227
H	-0.34770	-0.59302	-0.86673

Ethene biproduct in vacuum
Gibbs free energy at 298.15 K: -78.533814 a.u.

C	-0.00000	0.66289	0.00000
H	0.92359	1.23080	0.00000
H	-0.92355	1.23084	0.00000
C	-0.00000	-0.66289	0.00000
H	-0.92359	-1.23080	0.00000
H	0.92355	-1.23084	0.00000

NBD34

Gibbs free energy at 298.15 K: -883.874839 a.u.

O	-0.99865	1.90027	0.31360
C	-1.79005	1.03164	0.01920
O	-3.03907	1.29582	-0.40006
C	-3.43035	2.68080	-0.46157
C	-3.84065	3.19217	0.90569
C	-1.55083	-0.41686	0.08218
C	-0.37848	-1.07196	0.20120
C	1.01155	-0.60351	0.19051
C	1.44134	0.40780	-0.66995
C	2.76866	0.82289	-0.69911
C	3.69558	0.22534	0.15733
O	5.00744	0.56060	0.21499
C	5.46559	1.59608	-0.64222
C	3.28354	-0.79374	1.02239
C	1.96433	-1.20833	1.02551
C	-0.73276	-2.57352	0.23215
H	0.05100	-3.22886	0.60303
C	-1.28081	-2.91048	-1.16684
C	-2.43586	-2.26260	-1.30005
C	-2.66414	-1.47731	0.00209
H	-3.66972	-1.10744	0.17569
C	-2.07239	-2.49898	1.00603
H	-4.26703	2.69467	-1.15787
H	-2.60742	3.26251	-0.87684
H	-4.65340	2.58704	1.31067
H	-4.18795	4.22351	0.82018
H	-2.99805	3.16703	1.59722
H	0.72978	0.87623	-1.33817
H	3.06385	1.60181	-1.38876
H	6.52594	1.71165	-0.43335
H	4.94625	2.53463	-0.43265
H	5.32688	1.32470	-1.69184
H	4.01578	-1.24873	1.67833
H	1.66260	-1.99975	1.70233
H	-0.76260	-3.52190	-1.89249
H	-3.08628	-2.22358	-2.16310
H	-2.60777	-3.44839	1.01818
H	-1.96519	-2.09528	2.01393

QC38
Gibbs free energy at 298.15 K: -883.852971 a.u.

O	2.89099	2.28697	0.18527
C	1.99597	1.48428	0.02516
O	0.72289	1.83123	-0.17564
C	0.41814	3.23776	-0.17389
C	-1.07223	3.36303	-0.39398
C	2.17444	0.03069	0.02907
C	3.43815	-0.62731	-0.51267
C	3.11497	-0.75127	0.93860
C	2.06921	-1.87779	1.02137
C	1.87493	-2.31052	-0.41735
C	3.05768	-1.88640	-1.26038
C	1.12917	-1.11219	0.11716
C	-0.34314	-0.96033	0.18903
C	-1.13707	-1.12854	-0.94020
C	-2.51782	-0.93121	-0.89854
C	-3.11792	-0.55636	0.30298
O	-4.45048	-0.33546	0.45715
C	-5.27828	-0.47714	-0.68647
C	-2.33363	-0.39342	1.45024
C	-0.96559	-0.59387	1.38795
H	0.99202	3.72261	-0.96577
H	0.72756	3.66113	0.78344
H	-1.35601	2.93178	-1.35536
H	-1.35887	4.41573	-0.38759
H	-1.62035	2.84730	0.39649
H	4.24097	0.02811	-0.82288
H	3.70970	-0.34103	1.74123
H	1.83016	-2.47988	1.88546
H	1.31414	-3.21439	-0.61579
H	2.77682	-1.67991	-2.29541
H	3.86407	-2.62326	-1.24510
H	-0.67193	-1.41648	-1.87731
H	-3.10223	-1.07214	-1.79769
H	-6.28937	-0.25192	-0.35667
H	-5.23951	-1.49841	-1.07456
H	-4.98466	0.22475	-1.47158
H	-2.81973	-0.11006	2.37637
H	-0.36198	-0.46242	2.28006

TS for the thermal back reaction of NBD34/QC38

Gibbs free energy at 298.15 K: -883.785639 a.u.

O	3.23159	1.58130	-0.73691
C	2.12831	1.04844	-0.55795
O	1.05531	1.80308	-0.15962
C	1.27158	3.20675	0.00862
C	1.85979	3.51506	1.37379
C	1.84645	-0.32194	-0.75976
C	3.00806	-1.31260	-0.51241
C	2.70050	-1.20624	0.93825
C	1.56187	-1.95487	1.24491
C	1.15377	-2.56943	-0.08980
C	2.43806	-2.67728	-0.90284
C	0.74251	-1.14455	-0.12847
C	-0.66709	-0.78029	0.04356
C	-1.22336	0.20772	-0.77105
C	-2.58995	0.47254	-0.75465
C	-3.41892	-0.23693	0.11339
O	-4.75129	-0.04220	0.22647
C	-5.35105	0.95109	-0.59471
C	-2.87295	-1.22833	0.94167
C	-1.52323	-1.50375	0.89321
H	0.28362	3.65629	-0.09944
H	1.91705	3.57455	-0.78933
H	1.20589	3.13957	2.16347
H	1.96764	4.59459	1.50044
H	2.84193	3.05207	1.47617
H	3.98822	-1.00626	-0.85287
H	3.19594	-0.51017	1.60636
H	1.02722	-1.97971	2.17862
H	0.39090	-3.33628	-0.10019
H	2.24322	-2.74301	-1.97316
H	3.05791	-3.51293	-0.57432
H	-0.58406	0.76788	-1.43802
H	-2.98644	1.23422	-1.41171
H	-6.40938	0.94402	-0.34815
H	-5.21895	0.71271	-1.65287
H	-4.93082	1.93712	-0.38235
H	-3.53186	-1.77088	1.60828
H	-1.12909	-2.28352	1.53490

NBD35
Gibbs free energy at 298.15 K: -883.873309 a.u.

O	-0.65759	2.90699	0.28019
C	-0.52149	1.81099	-0.21746
O	0.36292	1.53965	-1.18229
C	1.26027	2.59560	-1.57383
C	2.45943	2.65086	-0.64999
C	-1.35054	0.64380	0.12650
C	-1.01246	-0.65302	0.14937
C	0.27548	-1.31791	-0.06878
C	0.32138	-2.49340	-0.81926
C	1.51737	-3.16796	-1.04415
C	2.68774	-2.67418	-0.48318
C	2.66822	-1.52013	0.29822
C	1.47102	-0.83654	0.50691
O	1.37700	0.27947	1.27241
C	2.47984	0.61274	2.10298
C	-2.31243	-1.42894	0.46317
H	-2.16692	-2.45711	0.78576
C	-3.26610	-1.20689	-0.72078
C	-3.58876	0.08528	-0.72901
C	-2.85026	0.74259	0.44894
H	-3.19556	1.72693	0.75363
C	-2.93908	-0.42487	1.46745
H	0.71485	3.53909	-1.58506
H	1.55403	2.33907	-2.59032
H	2.15116	2.88938	0.36892
H	3.15370	3.41937	-0.99521
H	2.97673	1.68923	-0.64949
H	-0.60246	-2.87518	-1.24165
H	1.53042	-4.07036	-1.64229
H	3.62838	-3.18870	-0.64002
H	3.58840	-1.16398	0.74136
H	2.15568	1.46027	2.70260
H	3.35252	0.90194	1.51290
H	2.73877	-0.22526	2.75537
H	-3.55009	-1.97352	-1.42885
H	-4.19754	0.61827	-1.44649
H	-3.96335	-0.68219	1.73912
H	-2.32503	-0.26430	2.35459

QC39

Gibbs free energy at 298.15 K: -883.852549 a.u.

O	-0.98329	-1.19159	-1.08467
C	-1.50155	-0.41968	-0.30982
O	-2.69893	-0.63358	0.25951
C	-3.41364	-1.81595	-0.14916
C	-4.12121	-1.59679	-1.47188
C	-0.92415	0.85842	0.11104
C	-1.68241	2.18132	0.04867
C	-1.17270	1.67735	1.36218
C	0.31427	2.06966	1.37650
C	0.54288	2.76380	0.05112
C	-0.77319	3.25176	-0.51530
C	0.56885	1.25826	0.11814
C	1.70432	0.37139	-0.20068
C	2.68900	0.71951	-1.11773
C	3.73870	-0.14979	-1.41709
C	3.79181	-1.39007	-0.79722
C	2.81593	-1.76595	0.12775
C	1.78327	-0.88361	0.43316
O	0.80674	-1.14159	1.34055
C	0.67478	-2.47416	1.80870
H	-4.12364	-1.99500	0.65647
H	-2.71310	-2.64963	-0.20108
H	-4.80398	-0.74826	-1.40258
H	-4.70063	-2.48549	-1.72896
H	-3.40134	-1.41071	-2.26957
H	-2.74339	2.13917	-0.15803
H	-1.79301	1.40707	2.20327
H	0.95569	2.16186	2.23991
H	1.47199	3.29712	-0.10113
H	-0.77704	3.26057	-1.60736
H	-1.04039	4.24441	-0.14530
H	2.62220	1.68276	-1.61164
H	4.49668	0.13851	-2.13490
H	4.59543	-2.08035	-1.02561
H	2.87635	-2.73533	0.60421
H	-0.22544	-2.49117	2.41902
H	1.53274	-2.76675	2.41957
H	0.56496	-3.16757	0.97038

TS for the thermal back reaction of NBD35/QC39
Gibbs free energy at 298.15 K: -883.785372 a.u.

O	-1.74990	1.58963	0.52819
C	-2.05726	0.57614	-0.11348
O	-3.38269	0.32375	-0.41826
C	-4.35101	1.20012	0.15827
C	-4.65785	0.82127	1.59691
C	-1.19428	-0.39054	-0.65037
C	-1.58165	-1.85234	-0.81266
C	-0.43928	-2.04490	-1.75111
C	0.73523	-1.99506	-1.01611
C	0.32126	-1.93588	0.47733
C	-1.09212	-2.51076	0.49214
C	0.24643	-0.60105	-0.15840
C	1.27347	0.45247	-0.19048
C	0.95502	1.67569	-0.79346
C	1.87942	2.70826	-0.87957
C	3.15434	2.52647	-0.35868
C	3.51165	1.31831	0.23296
C	2.58553	0.27933	0.31233
O	2.88419	-0.93063	0.83648
C	4.19916	-1.15475	1.33001
H	-5.23716	1.09089	-0.46808
H	-3.99985	2.23045	0.09256
H	-5.00955	-0.21106	1.65222
H	-5.43668	1.47252	2.00001
H	-3.76502	0.92391	2.21514
H	-2.57778	-2.06303	-1.18047
H	-0.51937	-2.06602	-2.83139
H	1.74828	-1.99542	-1.38192
H	1.07273	-2.19215	1.20518
H	-1.67002	-2.16442	1.35049
H	-1.07722	-3.60221	0.45992
H	-0.04494	1.80717	-1.18109
H	1.60394	3.64573	-1.34572
H	3.88636	3.32382	-0.41138
H	4.51207	1.19228	0.62187
H	4.20833	-2.18006	1.69051
H	4.42679	-0.47375	2.15302
H	4.93797	-1.03748	0.53403

NBD36

Gibbs free energy at 298.15 K: -922.965815 a.u.

O	-3.70709	1.65815	-0.16811
C	-2.58428	1.20725	-0.08046
O	-1.48930	1.94247	-0.28358
C	-1.68555	3.34000	-0.56725
C	-0.31377	3.96608	-0.67167
C	-2.30261	-0.20282	0.22546
C	-1.13996	-0.86850	0.37754
C	0.26112	-0.43273	0.42914
C	0.66305	0.71510	1.17203
C	1.97620	1.08855	1.22295
C	2.97458	0.35130	0.52894
C	4.34307	0.72453	0.55259
C	5.27988	-0.01473	-0.12306
C	4.89332	-1.16539	-0.85299
C	3.57869	-1.55128	-0.89099
C	2.58632	-0.80369	-0.20247
C	1.21963	-1.18173	-0.21773
C	-1.51511	-2.36581	0.44552
H	-0.73506	-3.01287	0.83892
C	-2.09503	-2.74337	-0.92766
C	-3.23977	-2.07757	-1.06545
C	-3.43219	-1.24483	0.21293
H	-4.42669	-0.84454	0.38663
C	-2.83901	-2.23937	1.24160
H	-2.27589	3.78090	0.23805
H	-2.25051	3.43180	-1.49686
H	0.23179	3.85296	0.26651
H	-0.41216	5.03007	-0.89221
H	0.26331	3.49744	-1.47044
H	-0.08759	1.28218	1.70692
H	2.27511	1.95764	1.79946
H	4.63494	1.60562	1.11402
H	6.32278	0.27846	-0.10110
H	5.64434	-1.74004	-1.38186
H	3.27739	-2.43216	-1.44776
H	0.93445	-2.07278	-0.76928
H	-1.60136	-3.38931	-1.64086
H	-3.90178	-2.05342	-1.92016
H	-3.38719	-3.17996	1.29728
H	-2.70627	-1.80294	2.23249

QC40

Gibbs free energy at 298.15 K: -922.941971 a.u.

O	-3.66028	1.59378	-0.03836
C	-2.57069	1.07271	0.07029
O	-1.43697	1.75507	0.25688
C	-1.53124	3.19286	0.30121
C	-1.57093	3.77694	-1.09698
C	-2.34225	-0.37211	-0.00482
C	-3.44947	-1.38109	0.25760
C	-2.90461	-1.24934	-1.12516
C	-1.59942	-2.06419	-1.13417
C	-1.50719	-2.60890	0.27112
C	-2.86003	-2.59138	0.94708
C	-1.03262	-1.19833	-0.03525
C	0.34808	-0.72881	0.20632
C	0.66639	-0.12728	1.45794
C	1.93939	0.28411	1.73469
C	2.98158	0.11804	0.77976
C	4.31342	0.53342	1.03670
C	5.29467	0.35961	0.09394
C	4.98789	-0.23819	-1.15205
C	3.70845	-0.64914	-1.42692
C	2.67317	-0.48320	-0.46997
C	1.33678	-0.89372	-0.72993
H	-0.63638	3.50428	0.83764
H	-2.41261	3.47114	0.87886
H	-0.68913	3.47156	-1.66255
H	-1.58230	4.86683	-1.03739
H	-2.46593	3.45011	-1.62741
H	-4.43304	-0.99011	0.47941
H	-3.45996	-0.88881	-1.97809
H	-1.10812	-2.48837	-1.99734
H	-0.75190	-3.35117	0.49251
H	-2.78165	-2.45135	2.02725
H	-3.43757	-3.49542	0.74065
H	-0.12356	0.00388	2.18958
H	2.17453	0.74193	2.68983
H	4.54222	0.99143	1.99324
H	6.30939	0.67971	0.29884
H	5.77103	-0.36963	-1.88939
H	3.46948	-1.10733	-2.38088
H	1.10518	-1.34139	-1.69131

TS for the thermal back reaction of NBD36/QC40
Gibbs free energy at 298.15 K: -922.877236 a.u.

O	3.05598	2.32614	-0.00260
C	2.19523	1.45043	-0.18658
O	1.05873	1.73962	-0.92013
C	0.76184	3.12184	-1.12282
C	0.09985	3.72029	0.10615
C	2.22422	0.14053	0.29029
C	3.49511	-0.61653	0.63805
C	2.73846	-1.39113	1.66178
C	1.88642	-2.26703	1.00368
C	2.23439	-2.16308	-0.50325
C	3.66156	-1.62201	-0.52178
C	1.35600	-1.05235	-0.09511
C	-0.10946	-1.04440	-0.21774
C	-0.79134	-2.03795	-0.97683
C	-2.15563	-2.03323	-1.06358
C	-2.92511	-1.04528	-0.39166
C	-4.34170	-1.01763	-0.45938
C	-5.05194	-0.05137	0.20541
C	-4.38151	0.93408	0.97175
C	-3.01407	0.93338	1.05365
C	-2.25053	-0.05347	0.37284
C	-0.83732	-0.07324	0.43572
H	0.08678	3.14416	-1.97896
H	1.67430	3.66075	-1.37890
H	-0.83117	3.19507	0.32981
H	-0.13040	4.77488	-0.06047
H	0.76459	3.64376	0.96912
H	4.34373	-0.03200	0.97102
H	2.74764	-1.18526	2.72552
H	1.11881	-2.89029	1.43058
H	1.90626	-2.97150	-1.14149
H	3.89581	-1.11334	-1.45748
H	4.39236	-2.40713	-0.32072
H	-0.22687	-2.79715	-1.50398
H	-2.66903	-2.78562	-1.65209
H	-4.85274	-1.77440	-1.04464
H	-6.13391	-0.03784	0.14892
H	-4.95790	1.68828	1.49391
H	-2.49478	1.68170	1.64270
H	-0.31786	0.68878	1.00539

NBD37 rotamer 1
Gibbs free energy at 298.15 K: -922.964090 a.u.

O	1.50649	2.75305	-0.07869
C	1.13795	1.70861	-0.57104
O	0.08766	1.59979	-1.38875
C	-0.72293	2.77447	-1.59320
C	-1.73442	2.92695	-0.47492
C	1.80954	0.42184	-0.33486
C	1.26832	-0.80494	-0.30016
C	-0.13084	-1.22280	-0.50078
C	-0.43485	-2.10280	-1.51304
C	-1.77183	-2.47782	-1.77857
C	-2.79203	-1.97292	-1.01828
C	-2.51859	-1.09409	0.06350
C	-1.17378	-0.72312	0.34617
C	-0.92158	0.10912	1.47095
C	-1.95092	0.56789	2.25124
C	-3.29029	0.22971	1.94608
C	-3.56439	-0.58527	0.87956
C	2.38733	-1.75653	0.16590
H	2.20690	-2.81077	-0.02960
C	2.68669	-1.37323	1.62852
C	3.21106	-0.14969	1.61330
C	3.26301	0.30340	0.14230
H	3.89863	1.15566	-0.08128
C	3.59569	-1.06190	-0.51466
H	-1.21072	2.60181	-2.55096
H	-0.07199	3.64494	-1.67318
H	-2.35047	2.02852	-0.39186
H	-2.38281	3.78023	-0.68384
H	-1.23424	3.09449	0.48014
H	0.36289	-2.49048	-2.13759
H	-1.98160	-3.15835	-2.59504
H	-3.82219	-2.24443	-1.22206
H	0.10023	0.37270	1.71894
H	-1.73953	1.19850	3.10683
H	-4.09530	0.60903	2.56428
H	-4.58638	-0.86375	0.64519
H	2.44958	-1.99357	2.48164
H	3.50827	0.46278	2.45342
H	4.55347	-1.46854	-0.19061
H	3.52345	-1.04289	-1.60294

QC41 rotamer1
Gibbs free energy at 298.15 K: -922.944433 a.u.

O	-2.26127	2.66946	-0.30600
C	-1.55644	1.68404	-0.35184
O	-0.26976	1.70742	-0.69614
C	0.35071	2.98517	-0.91534
C	1.83341	2.71878	-1.05148
C	-1.99839	0.32141	-0.03994
C	-3.20097	0.02801	0.84399
C	-3.24586	-0.35820	-0.59772
C	-2.46988	-1.68518	-0.68840
C	-2.04067	-1.95333	0.73585
C	-2.89827	-1.17564	1.70944
C	-1.22926	-1.02026	-0.14406
C	0.19466	-1.21065	-0.50309
C	0.54819	-1.84586	-1.66796
C	1.90802	-2.01851	-2.02681
C	2.90168	-1.53386	-1.22104
C	2.58064	-0.86651	-0.00714
C	1.21834	-0.71466	0.36623
C	0.91506	-0.05511	1.58645
C	1.91348	0.43909	2.38631
C	3.27014	0.29703	2.00774
C	3.59362	-0.34393	0.84035
H	0.12750	3.63281	-0.06544
H	-0.07486	3.43558	-1.81442
H	2.21473	2.23790	-0.14746
H	2.36759	3.65749	-1.20647
H	2.02792	2.06165	-1.90142
H	-3.78275	0.87387	1.18437
H	-3.91829	0.06236	-1.33038
H	-2.56257	-2.43744	-1.45769
H	-1.61123	-2.91696	0.97570
H	-2.35649	-0.90502	2.61867
H	-3.80754	-1.71617	1.98227
H	-0.22871	-2.21050	-2.33041
H	2.15336	-2.52732	-2.95162
H	3.94504	-1.64984	-1.49436
H	-0.12226	0.06050	1.88175
H	1.66636	0.94229	3.31375
H	4.05033	0.69405	2.64644
H	4.63086	-0.46126	0.54444

TS for the thermal back reaction of NBD37/QC41 rotamer1
Gibbs free energy at 298.15 K: -922.958609 a.u.

O	1.83961	2.75420	0.35319
C	1.23571	1.70443	0.42432
O	0.05919	1.57887	1.04196
C	-0.52953	2.78955	1.55612
C	-1.06714	3.68808	0.43836
C	1.73186	0.44348	-0.14485
C	3.17760	0.29674	-0.64245
C	3.89902	-0.59986	0.37712
C	3.33410	-1.80340	0.30660
C	2.23411	-1.72529	-0.76545
C	2.92942	-0.75423	-1.75485
C	1.14899	-0.76628	-0.22545
C	-0.18271	-1.26049	0.16257
C	-0.23901	-2.34185	1.01541
C	-1.47311	-2.86450	1.46030
C	-2.64810	-2.31544	1.02173
C	-2.63864	-1.23108	0.10552
C	-1.39853	-0.69510	-0.34248
C	-1.42279	0.36158	-1.29148
C	-2.60921	0.87250	-1.74801
C	-3.84204	0.35629	-1.28087
C	-3.85312	-0.67424	-0.37979
H	0.21671	3.30263	2.16238
H	-1.32520	2.43178	2.20543
H	-0.83545	3.25513	-0.53584
H	-0.61915	4.68076	0.47821
H	-2.15034	3.79210	0.50418
H	3.66979	1.22853	-0.90548
H	4.67583	-0.26305	1.04973
H	3.53960	-2.67996	0.90571
H	1.85523	-2.67183	-1.14277
H	2.26636	-0.39464	-2.54332
H	3.84877	-1.16533	-2.17220
H	0.68479	-2.78584	1.37193
H	-1.48047	-3.69973	2.15015
H	-3.60236	-2.70616	1.35823
H	-0.48554	0.76149	-1.65938
H	-2.60643	1.67863	-2.47246
H	-4.77304	0.77324	-1.64613
H	-4.79080	-1.08757	-0.02385

NBD37 rotamer2
Gibbs free energy at 298.15 K: -922.963055 a.u.

O	-1.54068	-1.89302	0.00537
C	-2.13076	-0.84153	-0.09713
O	-3.44953	-0.71691	0.11975
C	-4.16194	-1.90415	0.51838
C	-3.99672	-2.16602	2.00261
C	-1.52951	0.45321	-0.45716
C	-0.22661	0.71606	-0.65363
C	0.92938	-0.18982	-0.77337
C	0.95821	-1.10753	-1.79875
C	2.09191	-1.92132	-2.01996
C	3.19153	-1.80845	-1.21148
C	3.19889	-0.88571	-0.13283
C	2.05667	-0.06904	0.09769
C	2.06424	0.81502	1.20946
C	3.16104	0.90650	2.02741
C	4.30644	0.11220	1.78203
C	4.32013	-0.76622	0.73064
C	-0.12152	2.24551	-0.86579
H	0.81741	2.57948	-1.30104
C	-0.55744	2.93388	0.43829
C	-1.84608	2.65495	0.62081
C	-2.29113	1.78507	-0.56448
H	-3.36119	1.69498	-0.72296
C	-1.43182	2.43958	-1.67869
H	-3.80817	-2.74442	-0.07909
H	-5.19965	-1.69466	0.26490
H	-2.95336	-2.36792	2.24641
H	-4.59344	-3.03431	2.28813
H	-4.33913	-1.30684	2.58165
H	0.09847	-1.19484	-2.45291
H	2.08563	-2.62933	-2.84003
H	4.06919	-2.42327	-1.37909
H	1.18111	1.40581	1.42203
H	3.15043	1.58402	2.87299
H	5.16672	0.19457	2.43551
H	5.18744	-1.39040	0.54363
H	0.10544	3.49527	1.08271
H	-2.47858	2.92943	1.45385
H	-1.67094	3.48979	-1.84737
H	-1.44508	1.87886	-2.61452

QC41 rotamer2
Gibbs free energy at 298.15 K: -922.942200 a.u.

O	1.62164	2.77918	-0.03909
C	1.29449	1.66988	-0.39977
O	0.36065	1.42762	-1.32391
C	-0.39038	2.55083	-1.82662
C	-1.54310	2.87202	-0.89654
C	1.88759	0.42348	0.09999
C	3.39578	0.19771	0.11807
C	2.63110	0.19458	1.40276
C	2.01438	-1.21498	1.48382
C	2.47249	-1.90574	0.21681
C	3.69393	-1.21135	-0.34487
C	1.27106	-0.99615	0.17836
C	-0.11170	-1.36157	-0.20426
C	-0.34395	-2.28758	-1.19179
C	-1.65940	-2.60564	-1.60873
C	-2.73302	-1.97720	-1.03937
C	-2.54105	-1.01657	-0.00897
C	-1.22202	-0.71351	0.42795
C	-1.05499	0.22860	1.47847
C	-2.13871	0.84312	2.05241
C	-3.44842	0.55384	1.60412
C	-3.64246	-0.35714	0.59830
H	-0.74456	2.22505	-2.80316
H	0.28074	3.40019	-1.95228
H	-2.17792	1.99343	-0.75975
H	-2.14360	3.67872	-1.32161
H	-1.17463	3.18948	0.08036
H	4.02581	1.04501	-0.11816
H	2.77134	0.90479	2.20436
H	1.66586	-1.72367	2.37029
H	2.31956	-2.97308	0.12612
H	3.74859	-1.28302	-1.43314
H	4.62199	-1.59020	0.08926
H	0.49989	-2.77280	-1.66984
H	-1.80713	-3.34130	-2.39051
H	-3.74400	-2.20336	-1.36091
H	-0.05777	0.45933	1.83535
H	-1.99276	1.55897	2.85293
H	-4.29582	1.05052	2.06172
H	-4.64352	-0.59239	0.25223

TS for the thermal back reaction of NBD37/QC41 rotamer2
Gibbs free energy at 298.15 K: -922.876984

O	2.04639	2.76564	-0.41427
C	1.47302	1.66637	-0.53178
O	0.29556	1.56819	-1.25795
C	-0.39063	2.78348	-1.55056
C	-1.29315	3.19497	-0.40073
C	1.88905	0.44807	-0.02573
C	3.21921	0.07559	0.60812
C	2.49685	-0.50297	1.77800
C	1.84905	-1.66341	1.37319
C	2.34016	-1.93171	-0.07725
C	3.66197	-1.17593	-0.18080
C	1.26574	-0.93041	-0.07505
C	-0.13914	-1.24734	-0.39621
C	-0.38756	-2.09537	-1.45160
C	-1.70839	-2.37606	-1.86994
C	-2.76724	-1.82069	-1.20579
C	-2.55675	-0.96356	-0.09133
C	-1.23134	-0.66068	0.32394
C	-1.05167	0.18145	1.45092
C	-2.13135	0.68804	2.12858
C	-3.44889	0.39560	1.70826
C	-3.65431	-0.40984	0.61889
H	-0.97825	2.57162	-2.44527
H	0.33190	3.56599	-1.78354
H	-2.03079	2.41587	-0.19636
H	-1.81715	4.12224	-0.64492
H	-0.70446	3.35667	0.50533
H	3.92421	0.87615	0.79498
H	2.37814	-0.00532	2.73435
H	1.15968	-2.27180	1.93383
H	2.18942	-2.93300	-0.45835
H	3.91236	-0.92792	-1.21253
H	4.48059	-1.72011	0.29199
H	0.44289	-2.53190	-1.99379
H	-1.87128	-3.03155	-2.71651
H	-3.78551	-2.03105	-1.51492
H	-0.04993	0.44405	1.76325
H	-1.97415	1.33150	2.98638
H	-4.29158	0.80976	2.24882
H	-4.65949	-0.64550	0.28618

10. References

1. H. Hofmeister, K. Annen, H. Laurent and R. Wiechert, *Angew. Chem., Int. Ed.,* 1984, **23**, 727.
2. T. J. Korn and P. Knochel, *Angew. Chem., Int. Ed.,* 2005, **44**, 2947-2951.
3. M. Miura, T. Koike, T. Ishihara, F. Hirayama, S. Sakamoto, M. Okada, M. Ohta and S.-i. Tsukamoto, *Synth. Commun.,* 2006, **36**, 3809.
4. C. Wang, S. Rakshit and F and Glorius, *J. Am. Chem. Soc.,* 2010, **132**, 14006.
5. C. G. Hatchard and C. A. Parker, *Proc. R. Soc. London, Ser. A,* 1956, **235**, 518.
6. K. Stranius and K. Börjesson, *Sci. Rep.,* 2017, **7**, 41145.
7. R. Krishnan, J. S. Binkley, R. Seeger and J. A. Pople, *J. Chem. Phys.* 1980, **72**, 650.
8. Y.Zhao and DG. Truhlar, *Theor. Chem. Acc.* 2008,**120**, 215.
9. A. Erbs Hillers-Bendtsen, M. Quant, K. Moth-Poulsen and K. V. Mikkelsen. Benchmark investigation of the structural and thermochemical properties of a series of [2.2.2]-Bicyclooctadienes photoswitches, *submitted.*
10. N. M. O'Boyle, M. Banck, C. A. James, C. Morley, T. Vandermeersch and G. R. Hutchison, *J. Cheminf.* 2011, **3**, 33.
11. J. J. P. Stewart, *J. Mol. Model.,* 2007, **13**, 1173.
12. Gaussian 16, Revision C.01, M. J. Frisch et al., Gaussian, Inc., Wallingford CT, 2016.
13. E. Cances, B. Mennucci, J. Tomasi, *J. Chem. Phys.* 1997, **107**, 3032.
14. W. Moormann, T. Tellkamp, E. Stadler, F. Roehricht, C. Naether, R. Puttreddy, K. Rissanen, G. Gescheidt, R. Herges, *Angew. Chem., Int. Ed.,* 2020, **59**, 15081.
